# Graphitic Carbon Nitride/Zinc Oxide-Based Z-Scheme and S-Scheme Heterojunction Photocatalysts for the Photodegradation of Organic Pollutants

**DOI:** 10.3390/ijms241915021

**Published:** 2023-10-09

**Authors:** Gopal Panthi, Mira Park

**Affiliations:** 1 Carbon Composite Energy Nanomaterials Research Center, Woosuk University, Wanju 55338, Republic of Korea; 2 Woosuk Institute of Smart Convergence Life Care (WSCLC), Woosuk University, Wanju 55338, Republic of Korea

**Keywords:** electrospinning, water pollution, photocatalysis, g-C_3_N_4_, ZnO, Z-scheme and S-scheme photocatalysts, heterojunctions

## Abstract

Graphitic carbon nitride (g-C_3_N_4_), a metal-free polymer semiconductor, has been recognized as an attractive photocatalytic material for environmental remediation because of its low band gap, high thermal and photostability, chemical inertness, non-toxicity, low cost, biocompatibility, and optical and electrical efficiency. However, g-C_3_N_4_ has been reported to suffer from many difficulties in photocatalytic applications, such as a low specific surface area, inadequate visible-light utilization, and a high charge recombination rate. To overcome these difficulties, the formation of g-C_3_N_4_ heterojunctions by coupling with metal oxides has triggered tremendous interest in recent years. In this regard, zinc oxide (ZnO) is being largely explored as a self-driven semiconductor photocatalyst to form heterojunctions with g-C_3_N_4_, as ZnO possesses unique and fascinating properties, including high quantum efficiency, high electron mobility, cost-effectiveness, environmental friendliness, and a simple synthetic procedure. The synergistic effect of its properties, such as adsorption and photogenerated charge separation, was found to enhance the photocatalytic activity of heterojunctions. Hence, this review aims to compile the strategies for fabricating g-C_3_N_4_/ZnO-based Z-scheme and S-scheme heterojunction photocatalytic systems with enhanced performance and overall stability for the photodegradation of organic pollutants. Furthermore, with reference to the reported system, the photocatalytic mechanism of g-C_3_N_4_/ZnO-based heterojunction photocatalysts and their charge-transfer pathways on the interface surface are highlighted.

## 1. Introduction

The increased growth in industrialization, urbanization, and unlimited human activities up to the twenty-first century has led to significant water pollution problems. Various organic and toxic pollutants (dyes, pharmaceuticals, pesticides, etc.) generated from these activities are discharged into natural water bodies. Due to their carcinogenic nature and poor biodegradability, these organic pollutants serve as the main cause of the pollution of freshwater systems, creating a serious threat to all living beings around the globe [1,2]. Chercuiyot et al. reported that more than 50,000 tons of organic dye is discharged into the environment per year from different dyeing industries worldwide [3]. The presence of these pollutants in water, even at very low concentrations, decreases water quality and its aesthetic value, and the consumption of such contaminated water has adverse effects on aquatic as well as terrestrial lives. Therefore, water pollution has become an increasingly prominent problem for society and the economy, and proper management of these organic pollutants before discharging into natural water bodies seems to be prioritized for environmental remediation [4]. Considering these issues, various attempts, including oxidation, coagulation, ultrafiltration, ozonation, biological degradation, and photodegradation, have been made for their removal. However, certain disadvantages, including high operating costs, time consumption, and the inefficient removal of pollutants, have made the removal process more tedious [5]. 

In order to overcome these challenges, photocatalysis, a promising “green technique”, is being continuously explored for the removal of organic pollutants from aqueous media via photochemical reactions using semiconductor material/s [6]. Moreover, photocatalysis is regarded as a renewable technique for energy conversion and environmental restoration that involves oxidation and reduction reactions initiated by photogenerated charge pairs, i.e., electrons (e^−^) and holes (h^+^), under light irradiation [7,8]. The first study related to the photodegradation of organic pollutants was reported by Carey et al. in the presence of a TiO_2_ semiconductor photocatalyst in an aqueous solution [9]. Since then, the design and fabrication of highly efficient semiconductor-based photocatalysts have gained massive attention for this purpose, especially if the photocatalyst can show higher performance under visible-light irradiation than under ultraviolet light (UV light). Currently, the direct conversion of solar energy into chemical energy is accepted as one of the sustainable ways to solve the growing energy and environmental crises in the future [10,11]. Therefore, the utilization of sunlight in the form of visible light could be the best alternative to carry out the photocatalytic process in large-scale applications since sunlight is the cheapest source of energy and can make the process economically viable. For a photocatalyst to work under visible-light irradiation, the visible-light energy absorbed by the photocatalyst should be stronger than its band-gap energy to excite the valence-band (VB) electrons to the conduction band (CB). The desirable properties of visible-light-driven photocatalysts include (i) a narrow band gap of semiconductor materials to absorb visible light effectively, (ii) a low recombination rate and high migration of photogenerated electrons and holes, and (iii) good structural and chemical stability of the semiconductor. To obtain these properties, various attempts have been made, such as the doping of semiconductor materials with metals or non-metals, the deposition of noble metals on the surface of semiconductor materials, the use of supportive materials, the surface modification of photocatalysts via different synthesis techniques, and the formation of heterojunctions. The utilization of these strategies can provide a feasible means for fabricating visible-light-responsive photocatalysts with enhanced performance [12,13,14,15].

The general photocatalytic reactions that take place in a photocatalyst during the degradation of organic pollutants are explained in the literature. When light irradiates a photocatalyst, electrons and holes are generated in the CB and VB, respectively, as follows:

(1) $$Photocatalyst\overset{h\nu }{\to }h^{+}\;(VB)+e^{-}\;(CB)$$

Here, hν is the light energy required to excite electrons from the VB to the CB of the photocatalyst. These photogenerated electrons and holes act as powerful reducing and oxidizing agents, respectively. During the photocatalytic process, the photogenerated electrons in the CB of the photocatalyst react with dissolved oxygen (electron acceptor) in the solution to generate oxygen peroxide (O_2_^•−^) radicals, while the photogenerated holes in the VB of the photocatalyst react with adsorbed water (H_2_O) or hydroxyl ions (OH^−^) to generate hydroxyl (OH^•^) radicals as follows:

(2) $$\left(e_{CB}^{-}\right)+O_{2}\overset{}{\to }O_{2}^{•-}\;(\mathrm{Reduction}\;\mathrm{reaction})$$

(3) $$\left(h_{VB}^{+}\right)+H_{2}O\overset{}{\to }{OH}^{•}\;(\mathrm{Oxidation}\;\mathrm{reaction})$$

(4) $$\left(h_{VB}^{+}\right)+{OH}^{-}\;\;\overset{}{\to }{OH}^{•}\;(\mathrm{Oxidation}\;\mathrm{reaction})$$

The O_2_^•−^ and OH^•^ radicals are known as reactive oxygen species (ROS) and are responsible for the photodegradation of organic pollutants, but in some cases, photogenerated holes are directly involved in the oxidation reaction rather than forming OH^•^ radicals. Utilizing these ROS or holes, the photocatalytic process proceeds for the degradation of organic pollutants as follows:

(5) $$O_{2}^{•-}/{OH}^{•}/h^{+}+Organic\;pollutants\overset{}{\to }{CO}_{2}+H_{2}O+harmless\;chemicals$$

It is noteworthy that the oxidation and reduction reactions may not occur simultaneously in photocatalysis. So, the possibility of the recombination of the photogenerated electrons and holes remains, which requires the efficient consumption of these charge carriers through their effective separation. Considering this issue, various strategies regarding the design and synthesis of Z-scheme and S-scheme heterojunction photocatalysts have been intensively reported [8].

In the realm of fabrication strategies for heterojunction photocatalysts with enhanced photocatalytic activities for the photodegradation of organic pollutants, several reports focused on g-C_3_N_4_- and ZnO-based heterojunction photocatalysts have been published so far [16,17,18]. g-C_3_N_4_ is a typical organic semiconductor material having a low band gap (approximately 2.7 eV) with CB and VB positions at −1.1 eV and +1.6 eV vs. normal hydrogen electrodes (NHEs), respectively. Therefore, it has attracted more attention from researchers aiming to study its visible-light-assisted photocatalysis in the last decade. The photocatalytic activity of g-C_3_N_4_ for water splitting was first reported by Wang et al. [19]. Since then, numerous efforts have been made to fabricate superior and visible-light-driven g-C_3_N_4_ photocatalysts due to their low cost, high stability, non-toxicity, facile synthesis, and suitable band structure [20,21,22]. Unfortunately, the high recombination of photogenerated charge carriers due to the strong coulombic force of attraction, inadequate visible-light response, and weak oxidation ability of photogenerated holes still hinder the photocatalytic performance of pristine g-C_3_N_4_ [23,24,25]. Therefore, the design and synthesis of g-C_3_N_4_ heterojunctions coupled with ZnO for achieving higher photocatalytic performance has become a new research hotspot and drawn wide interdisciplinary attention as a visible-light-responsive photocatalyst for solar energy conversion and environmental remediation. Moreover, ZnO is a well-known promising semiconductor possessing high quantum efficiency, high electron mobility, low cost, and environmental friendliness [26,27].

In a heterojunction photocatalyst, photogenerated charge carriers are impelled to transfer toward opposite directions due to band bending at the interface, so the redox abilities of heterojunctions become stronger by utilizing the more negative CB of one component and the more positive VB of another component [28,29]. In addition, some defects may occur due to a non-matching lattice at the interface, which also promotes the diffusion of photogenerated charge carriers [30]. Hence, this review provides a brief introduction, along with a comprehensive fabrication strategy, on visible-light-responsive g-C_3_N_4_/ZnO-based Z-scheme and S-scheme heterojunction photocatalysts for the photodegradation of organic pollutants. Moreover, the photocatalytic mechanism and charge-transfer pathways involved in the photodegradation process utilizing Z-scheme and S-scheme heterojunctions of g-C_3_N_4_ and ZnO are summarized from the recently published reports.

## 2. Structure of g-C_3_N_4_

Usually, g-C_3_N_4_ appears as a yellow powder under visible light. It is a metal-free polymeric semiconductor with a general formula of (C_3_N_3_H)_n_ that consists of a carbon lattice partially substituted with nitrogen atoms in a regular fashion resembling the layered structure of graphene. In the g-C_3_N_4_ structure, carbon atoms are bonded through sp^2^ hybridization, and each sp^2^-bonded carbon atom is attached to a nitrogen atom (N-C=N). Also, the sp^2^-bonded nitrogen atom is directly bonded to sp^2^-bonded carbon (C-N=C) [31]. This material exhibits the properties of an n-type semiconductor, high chemical and photostability, and high photocatalytic stability under visible-light irradiation [32]. According to the first-principles density functional theory (DFT) calculations, seven phases of carbon nitride are predicted, namely, α-C_3_N_4_, β-C_3_N_4_, cubic C_3_N_4_, pseudocubic C_3_N_4_, g-h-triazine, g-o-triazine, and g-h-heptazine [33]. Among these, g-C_3_N_4_ is regarded as a unique phase due to its relatively low band gap due to the presence of sp^2^-hybridized carbon and nitrogen to form a π-conjugated electronic structure [34]. Hence, it is considered a suitable candidate for visible light activation at around 450–460 nm [35].

**Figure 1:**
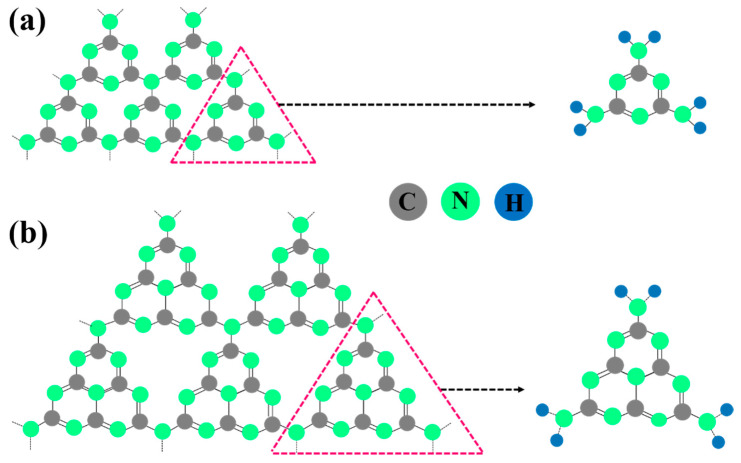
Structures of (**a**) triazine and (**b**) heptazine.

The basic units of g-C_3_N_4_ are triazine (C_3_N_3_) and tri-s-tirazine/heptazine (C_6_N_7_). Compared to triazine, heptazine is the energetically favorable and most stable phase at ambient conditions. Subsequently, heptazine phase (also known as melem)-based g-C_3_N_4_ has the highest thermal stability, in which layers formed by heptazine rings linked by a π-conjugated system are bonded through van der Waals forces of attraction [36]. In addition, thermogravimetric analysis (TGA) has evidenced its excellent thermal stability, as it can withstand heat without damage even at 600 °C in air, which can be attributed to the aromatic C-N heterocycles [21]. In addition, experimental results have revealed the good chemical stability of g-C_3_N_4_ toward common solvents. It is insoluble in water, alcohols, toluene, tetrahydrofuran, diethylether, and dimethyl formamide [37]. Importantly, the structural features of g-C_3_N_4_ can be controlled through different synthetic routes, including varying condensation temperature, altering the ratio of precursors, introducing porosity by applying hard/soft templates, and doping and exfoliation. Figure 1 shows the structures of triazine and heptazine, the basic units of g-C_3_N_4_.

## 3. Synthesis of g-C_3_N_4_

**Figure 2:**
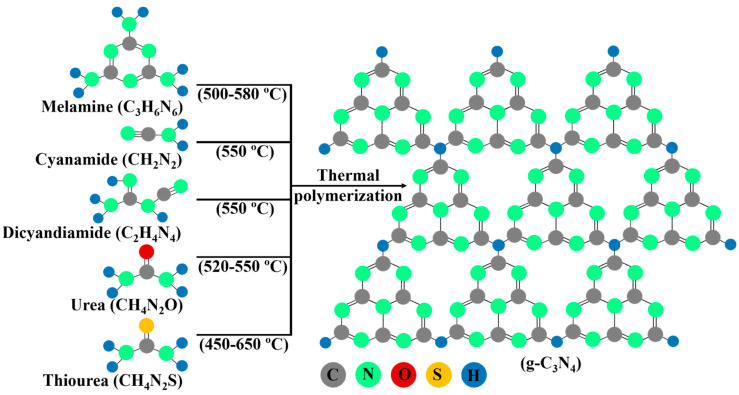
Schematic representation of synthesis process of g-C_3_N_4_ by thermal polymerization of different nitrogen-rich precursors.

Nitrogen-rich precursors containing pre-bonded C-N core structures, including melamine [38,39,40], cyanamide [41,42,43], dicyandiamine [44,45,46], urea [47,48,49], and thiourea [50,51,52], are widely used for the facile synthesis of g-C_3_N_4_ by the heat treatment process between 450 and 650 °C. The schematic representation of the synthesis process of g-C_3_N_4_ by thermal polymerization of different nitrogen-rich precursors is shown in Figure 2. Other precursors, such as guanidinium chloride [53,54,55], guanidine thiocyanate [56], thiourea oxide [57], ammonium thiocyanate, carbamide powder with thiourea, sulfur-mixed melamine, and sulfuric acid-mixed melamine [31,58,59], are also used for synthesizing g-C_3_N_4_. It is noteworthy that many points that strongly influence the physicochemical properties of g-C_3_N_4_ have to be considered during its synthesis. Many research findings have shown that different types of precursors and synthesis conditions can alter the surface area, porosity, adsorption behavior, photoluminescence, and carbon-to-nitrogen (C/N) ratio [60]. A lower C/N ratio causes a negative impact on charge separation and migration due to the occurrence of a large number of defects in g-C_3_N_4_, which ultimately lowers its photocatalytic activity [21,31]. The band gap of g-C_3_N_4_ can be tuned, creating small defects in it, by adjusting the C/N ratio. However, synthesizing g-C_3_N_4_ with the exact theoretical ratio of C/N is not easy.

### Synthetic Pathways of g-C_3_N_4_

**Figure 3:**
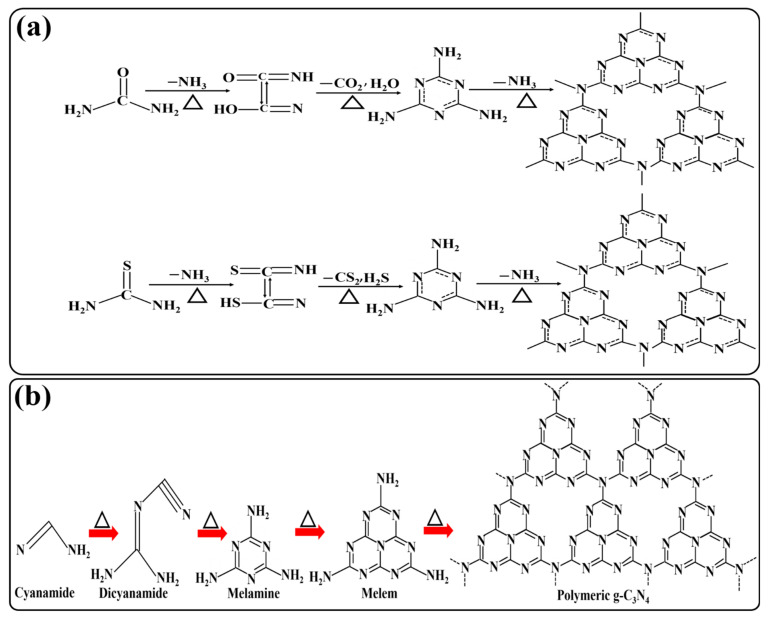
(**a**) High-temperature self-polymerization of urea and thiourea in air to form graphitic carbon nitride and (**b**) graphitic carbon nitride synthesis pathway using cyanamide as a precursor. Reprinted from Ref. [35] with permission from Elsevier.

The synthesis of g-C_3_N_4_ by urea self-polymerization is similar to that of the thiourea precursor. In both synthetic routes, ammonia gas is liberated during the polymerization process, along with the generation of different intermediates. Also, urea polymerization eliminates carbon dioxide gas and water, while thiourea involves the elimination of carbon disulfide and hydrogen sulfide gases. The gases, namely, ammonia liberated at 200 °C and carbon dioxide liberated at higher temperatures, play important roles in the formation of porous g-C_3_N_4_ [35] (Figure 3a). In another pioneer work reported by Wang et al., g-C_3_N_4_ was prepared using cyanamide as a precursor. In this process, the condensation reaction occurred at 203 and 234 °C, resulting in the formation of an amide and melamine, respectively [35]. On increasing the temperature to 335 °C, the condensation process led to the formation of melamine-based products, along with the removal of ammonia. On further heating at 390 °C, the heptazine unit was formed as a result of melamine rearrangement. Finally, thus-obtained heptazine was converted to the polymeric form of g-C_3_N_4_, which was decomposed, forming cyano and nitrogen fragments at 700 °C without leaving any residue. The synthetic pathway of g-C_3_N_4_ from the cyanamide precursor is shown in Figure 3b. 

**Figure 4:**
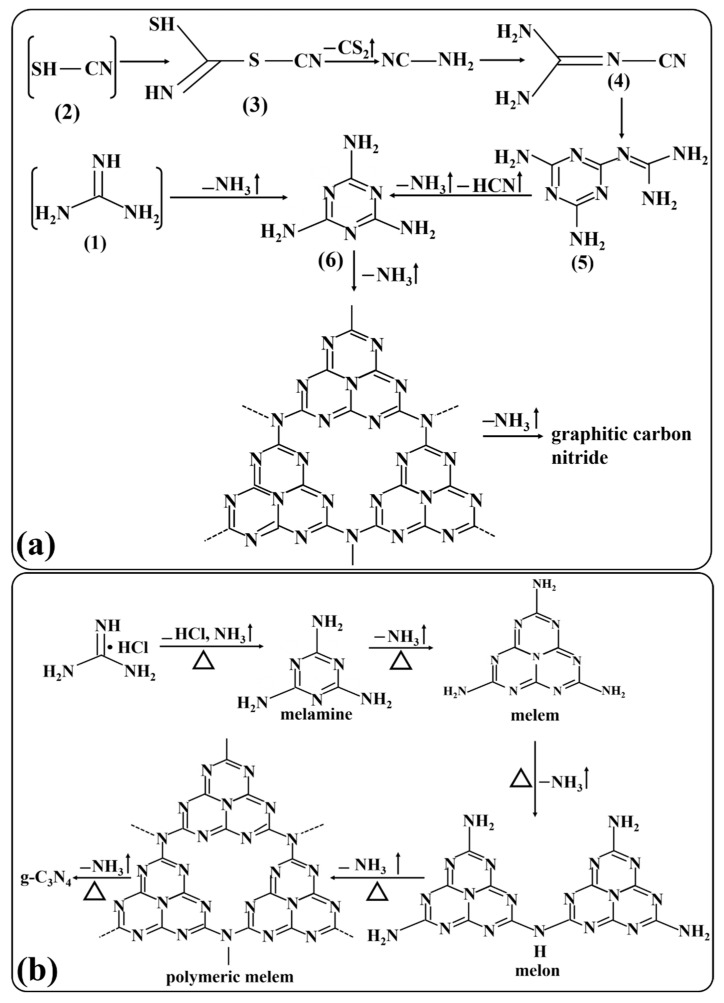
Thermal graphitic carbon nitride self-polymerization synthesis route for (**a**) guanidine thiocyanate and (**b**) guanidine hydrochloride. Reprinted from Ref. [35] with permission from Elsevier.

In another synthetic process of g-C_3_N_4_, guanidinium-based precursors are used as novel precursors. As reported by Schnick and coworkers, guanidinium dicyanamide and guanidinium tricyanomelaminate were used for the heat condensation synthesis of g-C_3_N_4_ [61]. It was confirmed that melamine was one of the important intermediates, and guanidium-based chemicals are the building blocks of g-C_3_N_4_. Similarly, Long et al. [62] synthesized g-C_3_N_4_ using the desulfurization of guanidium thiocyanate. Thiocyanic acid and guanidine, produced as the first products, reacted at the same time to obtain the final products (Figure 4a). Likewise, mesoporous g-C_3_N_4_ was prepared from guanidine hydrochloride using a thermal condensation process at 390 °C. The intermediate products melon, melem, and melamine were polymerized to form the final products [63] (Figure 4b).

## 4. Morphologies of g-C_3_N_4_

**Figure 5:**
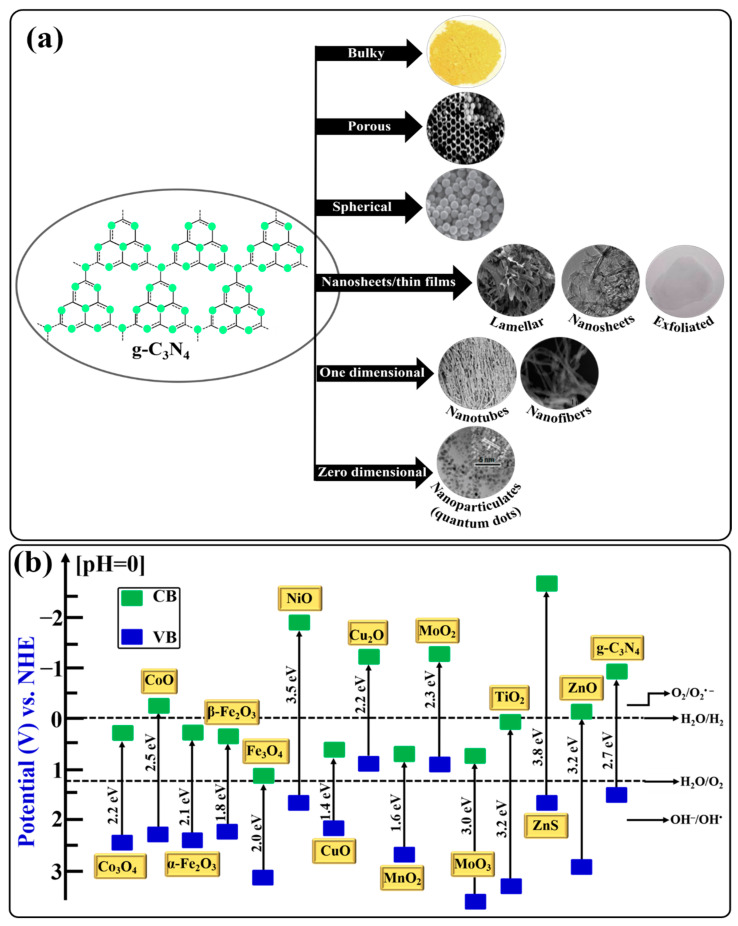
(**a**) Different morphological structures of g-C_3_N_4_ and (**b**) different band structures of some representative photocatalysts.

Depending upon the synthetic routes, condensation temperatures, and material compositions, different morphologies of g-C_3_N_4_, such as bulky, porous, spherical, nanosheets/nanofilms, one-dimensional, and zero-dimensional, have been reported, and such studies are still ongoing day after day. Different morphological structures of g-C_3_N_4_ strongly relate to its properties and applications. For the convenience of the reader, different morphological structures of g-C_3_N_4_ in terms of decreasing dimensions are shown in Figure 5a and discussed below.

### 4.1. Bulky g-C_3_N_4_

The bulk phase of g-C_3_N_4_ is regarded as the starting material for comparison. As discussed earlier in Section 3, it can be synthesized by the thermal condensation process of different nitrogen-rich precursors. Unfortunately, bulky g-C_3_N_4_ synthesized by this process possesses a very low surface area (10 m^2^g^−1^), has poor water solubility, and exhibits poor photocatalytic performance. On that note, Jun and Shalom adopted a new method to synthesize bulk g-C_3_N_4_ with a relatively high surface area (60–80 m^2^g^−1^) by utilizing the pre-organized supramolecular complex of melamine and cyanuric acid via topotactic transformation [64,65]. It is also reported that an increase in calcination temperature with a slower heating rate can marginally improve the surface area and crystallinity of g-C_3_N_4_ [66].

### 4.2. Porous g-C_3_N_4_

The presence of continuous porosity as an active center and a high surface area are important requirements for a photocatalyst to achieve enhanced photocatalytic performance. Therefore, the introduction of porosity into g-C_3_N_4_ can also significantly enhance its photocatalytic efficiency. The exposed surface area and accessible channels are increased in porous g-C_3_N_4_, thereby promoting light harvesting, the separation/migration of photogenerated charge carriers, molecular mass transfer, and surface reactions [67]. Hence, the formation of porous g-C_3_N_4_ as a photocatalyst material with a controlled pore size has attracted more interest for the photodegradation of organic pollutants. Generally, soft- and hard-templating techniques are applied for the formation of porous g-C_3_N_4_. Different templates could be used for this purpose. For instance, KIT-6 (a type of mesoporous silica), KCC-1 (high-surface-area silica nanosphere with fibrous surface), and SBA-15 (stable mesoporous silica sieve) are usually employed as hard templates in order to obtain nanostructured g-C_3_N_4_ [68]. In addition, bubble-forming templates (like urea and thiourea) and plunoric P123 are used as soft templates [69]. Moreover, metal–organic frameworks (MOFs) and calcium salts have also been used as templates for the fabrication of g-C_3_N_4_ porous nanostructures with enhanced surface properties [70,71].

A three-dimensional porous g-C_3_N_4_ was prepared by Zhang and coworkers via a facile bottom-up supramolecular self-assembly route using melamine and cyanuric acid heated at 550 °C in a muffle furnace [72]. Similarly, uniformly distributed mesoporous carbon nitride particles could be prepared by etching the silica template present in synthesized spheres [73,74,75]. Interestingly, porous g-C_3_N_4_ was successfully synthesized by Gu et al. without the use of templates [76]. The prepared sample showed a similar composition to that of bulk g-C_3_N_4_ but with a nanoporous surface, lower resistivity, and a narrow band gap. These properties accelerated the separation and migration of photogenerated charge carriers and the light-harvesting capacity in the visible region. 

### 4.3. Spherical g-C_3_N_4_

Photocatalysts with a spherical shape are regarded as more suitable candidates for photocatalytic applications. A photocatalyst is considered even better if it has a hollow spherical shape because a hollow sphere allows better light absorption and the enhanced mobility of photogenerated electron–hole pairs. However, a major drawback associated with hollow spheres is the possibility of their collapse during the synthesis process. In the early days, spherical silica core–shell structures were being used as hard templates for synthesizing hollow spheres of g-C_3_N_4_, but currently, triazine molecules are used for their synthesis. In this synthesis process, triazine molecules gather to form a supramolecular network through hydrogen bonding. The precursor material commonly employed in this process is a complex of melamine and cyanuric acid. Different forms of hollow spheres exhibiting good photocatalytic activity have been reported in previous research; these include hollow boxes, mesoporous hollow spheres, and hollow 3D assemblies [77]. Recent studies have reported that hollow spheres of g-C_3_N_4_ can be prepared through non-templating methods. For example, Cui et al. synthesized g-C_3_N_4_ hollow spheres by a one-step solvothermal process without using a template [78].

### 4.4. Nanosheets and Nanofilms of g-C_3_N_4_

The fabrication of g-C_3_N_4_ in the form of nanosheets or nanofilms can improve the photoactivated transformation efficiency due to the high specific surface area and high porosity with respect to the bulk material. However, the fabrication processes of g-C_3_N_4_ in these forms are more tedious. Generally, the sonication–exfoliation method [79], the bottom-top method [80], or separate steps [81] are applied for fabricating g-C_3_N_4_ nanosheets. A facile template-free method for the preparation of ultrathin nanosheets of g-C_3_N_4_ via the thermal polycondensation of a urea precursor was reported by Zhang et al. [82]. Based on the exfoliation technique, significant works were reported by Zhang et al. and Yang et al. [83,84], including the fabrication of exfoliated thin-layered g-C_3_N_4_ nanosheets from bulk g-C_3_N_4_ by a sonication-assisted liquid-exfoliation top-down method. Similarly, Niu et al. reported another protocol for a thermal-oxidation route to exfoliate dicyandiamide-derived bulky g-C_3_N_4_ into nanosheets exhibiting a high surface area [85]. g-C_3_N_4_ nanofilms have also been suggested as the most promising candidates for the development of flat devices possessing a high surface area and high charge carrier mobility. For instance, g-C_3_N_4_ films were developed on conductive substrates by Liu et al., who applied a nanoconfinement method [86]. Similarly, employing dual templates, Jia and coworkers developed a hierarchical macro-/mesoporous g-C_3_N_4_ film for use in sensing applications [87]. 

### 4.5. One-Dimensional g-C_3_N_4_ Nanostructures

One-dimensional g-C_3_N_4_ nanostructures have received tremendous attention in recent years for their use in photocatalytic applications due to their high surface area, potential electrical/optoelectrical properties, and high light-harvesting capability. Taking advantage of one-dimensional g-C_3_N_4_ nanostructures, different forms of g-C_3_N_4_ (nanowires, nanorods, nanotubes, and nanofibers) have been reported so far. Liu et al. reported the diatom-frustule-mediated direct growth of g-C_3_N_4_ nanowires with a larger length-aspect ratio for photocatalytic applications [88]. In another work, Zheng et al. introduced a new technique to fabricate twisted hexagonal rod-like C_3_N_4_ following a nanocasting technique using chiral silicon dioxide as a template [89]. Likewise, Li and coworkers fabricated g-C_3_N_4_ nanorods via the confined thermal condensation of cyanamide inside the nanochannels of an anodic alumni oxide membrane template [90]. The fabrication of another nanostructural form of g-C_3_N_4_ (i.e., nanotubes) for photocatalytic applications is also holding considerable interest. A facile route to obtain g-C_3_N_4_ nanotubes was reported by Wang’s group by directly heating melamine without a template. The thus-obtained nanotubes exhibited blue fluorescence and good photocatalytic properties [91]. A sulfur-mediated self-templating method was introduced by Liu and coworkers for the fabrication of one-dimensional hollow g-C_3_N_4_ nanostructures [92]. Furthermore, Tong et al. synthesized a tubular g-C_3_N_4_ isotype heterojunction photocatalyst using combined hydrothermal and condensation techniques [93]. In a different work, Tahir et al. fabricated g-C_3_N_4_ nanofibers without using any hard or soft templates by a green and facile method. The as-prepared g-C_3_N_4_ nanofibers exhibited excellent photocatalytic activity for the photodegradation of Rh B [94].

### 4.6. Zero-Dimensional g-C_3_N_4_ Nanostructures

Nanostructured materials having a size of less than 10 nm and containing a few thousand atoms show a significant quantum confinement effect. Moreover, these nanostructures possess excellent properties, like bright fluorescence, good stability, water solubility, biocompatibility, and non-toxicity. However, semiconductor quantum dots of some essential elements, like CdSe, PbTe, and CdTe, have long-term toxicity and potential environmental health hazards. Therefore, the synthesis of quantum dots of metal-free semiconductor materials, i.e., g-C_3_N_4_, has attracted keen interest in the past decades. Groenewolt et al. synthesized carbon nitride quantum dots by sacrificial templating using mesoporous silica host matrices. The as-prepared 5 nm sized quantum dots showed blue-shifted photoluminescence [95]. In another approach, a microwave-assisted method was applied to synthesize g-C_3_N_4_ quantum dots. Barman et al. reported a simple microwave-mediated method to fabricate highly blue fluorescent g-C_3_N_4_ quantum dots from formaldehyde [96]. Similarly, Cao et al. developed a method for the facile microwave-assisted fabrication of g-C_3_N_4_ quantum dots [97]. A solid-phase method was adopted by Zhou et al. to synthesize blue fluorescent g-C_3_N_4_ quantum dots [98]. Hydrothermal treatment is commonly utilized as a simple and green technique for synthesizing g-C_3_N_4_ quantum dots. On that note, Zhang et al. synthesized blue fluorescent g-C_3_N_4_ quantum dots from bulk g-C_3_N_4_ [99]. Likewise, g-C_3_N_4_ quantum dots with strong blue photoluminescence were synthesized by Lu et al. following the hydrothermal treatment of citric acid and thiourea [100]. In addition, g-C_3_N_4_ quantum dots have also been synthesized by top-down and bottom-up approaches. For the first time, Wang et al. prepared g-C_3_N_4_ quantum dots by using a thermal–chemical etching process utilizing a top-down approach [101]. An exfoliation strategy was applied by Xie and coworkers to obtain single-layered g-C_3_N_4_ quantum dots exhibiting excellent two-photon absorption behavior [102].

## 5. Heterojunction Photocatalysts

As mentioned in the introduction section, a heterojunction photocatalyst can be a potential candidate to utilize sunlight to obtain enhanced photocatalytic activities. Heterojunctions provide an internal electric field upon light irradiation that facilitates the separation of electrons and holes and induces faster carrier migration to prevent their recombination [103]. Generally, a heterojunction involves the combination of two or more different semiconductors having a close contact interface, different band energy levels, matching crystal lattices, and similar thermal expansion coefficients. Furthermore, the dynamics of photogenerated electrons and holes in the heterojunction of combined semiconductors can be governed by their band-gap energy, the lowest potential of the CB, and the highest potential of the VB [104]. According to the migration of photogenerated electrons and holes, heterojunctions can be divided into conventional-type (I, II, and III) heterojunction, p-n heterojunction, Schottky junction, Z-scheme heterojunction, and step-scheme (S-scheme) heterojunction photocatalysts. In the past decades, conventional type II heterojunction photocatalysts have been widely studied, and several reports have been published regarding their enhanced photocatalytic performance. Even so, some noticeable issues are regarded as limiting factors for the large-scale applications of such photocatalysts. For instance, the redox ability of type II heterojunctions is greatly reduced since redox reactions in these photocatalysts occur for semiconductors with lower reduction and oxidation potential. Moreover, the migration of photogenerated electrons to the electron-rich CB and holes to the hole-rich VB experiences more difficulties due to electrostatic repulsions [8]. In order to overcome these limitations and respond to current demands, the fabrication of new types of heterojunction photocatalytic systems, namely, Z-scheme heterojunctions and S-scheme heterojunctions, have been introduced. For a comprehensive understanding of g-C_3_N_4_/ZnO-based Z-scheme and S-scheme heterojunction photocatalytic systems, it is important to discuss the introduction and development of Z-scheme (first to third generations and double Z-scheme) and S-scheme heterojunctions.

### 5.1. Z-Scheme Heterojunction Photocatalysts

Mimicking the natural photosynthesis process that takes place in chlorophyll in green plants for the formation of carbohydrates from H_2_O and CO_2_ in the presence of solar radiation via a two-step photoexcitation process, Bard, in 1979, originally introduced the concept of the Z-scheme heterojunction photocatalytic system [105]. In a Z-scheme heterojunction photocatalytic system, two semiconductor photocatalysts are coupled together. Based on their band structures, semiconductor photocatalysts are classified into oxidation photocatalysts (OPs) and reduction photocatalysts (RPs). An OP possesses a low VB position and exhibits a strong oxidation ability, while the RP possesses a higher CB position and exhibits a strong reduction ability. Some of the representative OPs and RPs with their band structures are illustrated in Figure 5b. Furthermore, several advantages, including an extended light-harvesting range, the ability to separate photogenerated electrons–holes, the simultaneous preservation of strong oxidation and reduction ability, and the spatial separation of reductive and oxidative sites, are enough to attract attention toward Z-scheme heterojunction photocatalysts [106,107]. 

**Figure 6:**
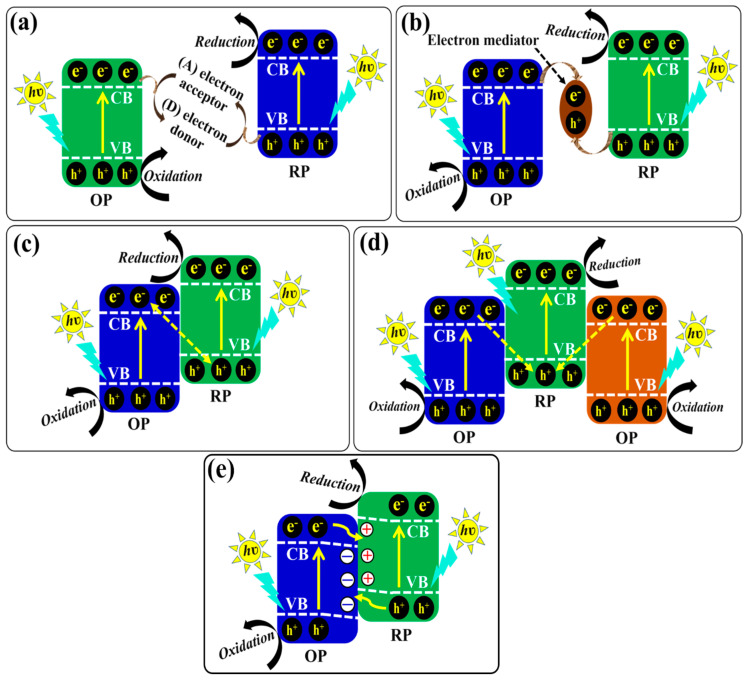
Schematic illustrations of different types of heterojunction photocatalysts along with charge-transfer processes; (**a**–**d**) Z-scheme heterojunctions and (**e**) S-scheme heterojunction.

Depending on the presence/absence of a charge carrier and the type of charge carrier mediator used, Z-scheme heterojunction photocatalytic systems are divided into three types: (1) traditional Z-scheme heterojunction photocatalysts (first generation); all-solid-state Z-scheme heterojunction photocatalysts (second generation); and (3) direct Z-scheme heterojunction photocatalysts (third generation). In addition, another new type, i.e., a double Z-scheme heterojunction photocatalyst formed by coupling two Ops and one RP, has also been introduced. Schematic illustrations of different types of Z-scheme heterojunction photocatalysts, along with their charge-transfer processes, are shown in Figure 6. 

#### 5.1.1. Traditional Z-Scheme Heterojunction Photocatalysts

The traditional Z-scheme heterojunction photocatalyst consists of two different semiconductor photocatalysts (OP and RP) coupled through a reversible redox ion pair (such as Fe^3+^/Fe^2+^, IO^3−^/I^−^, NO^3−^/NO^2−^, I^3−^/I^−^, [Co(phen)]^3+/2+^) as an electron-transfer medium. There is no physical contact between these two photocatalysts [8]. When the heterojunction is irradiated with light, electrons and holes are generated in the CB and VB, respectively, of each semiconductor photocatalyst. Then, with the support of an electron acceptor (A) and an electron donor (D) (A/D pairs), the photogenerated electrons are indirectly transferred from the CB of the OP to the VB of the RP, as shown in the following redox reactions of A/D pairs.

(6) $$A+e^{-}\overset{}{\to }D\;(CB\;of\;OP)$$

(7) $$D+h^{+}\overset{}{\to }A\;(VB\;of\;RP)$$

Finally, the photogenerated holes in the VB of the OP and the photogenerated electrons in the CB of the RP, having a strong redox ability, are significantly separated to participate in oxidation and reduction reactions, respectively. Therefore, the oxidative and reductive sites are separated, which in turn enhances the photocatalytic performance. Contrastingly, backward reactions may also occur in this system: i.e., A and D can react with the photogenerated electrons in the CB of the RP and holes in the VB of OP, respectively, as shown in the following reactions.

(8) $$A+e^{-}\overset{}{\to }D\;(CB\;of\;RP)$$

(9) $$D+h^{+}\overset{}{\to }A\;(VB\;of\;OP)$$

Unfortunately, traditional Z-scheme heterojunction photocatalysts have certain drawbacks, like backward reactions that arise due to the use of redox mediation; the application of the photocatalyst in the liquid phase, which greatly limits the application in gas and solid phases; and deactivation and a decrease in the reaction rate due to the instability of the redox mediator [108,109]. These drawbacks are responsible for the sharp decrease in the effective number of photogenerated electrons and holes and the reduction in performance. A schematic of the charge-transfer process in a traditional Z-scheme heterojunction photocatalyst is shown in Figure 6a.

#### 5.1.2. All-Solid-State Z-Scheme Heterojunction Photocatalysts

In order to overcome the aforementioned problems related to traditional Z-scheme heterojunction photocatalysts, Tada et al. introduced the concept of the all-solid-state Z-scheme photocatalyst for the first time [110]. All-solid-state Z-scheme heterojunction photocatalysts are constructed by coupling the OP and RP through solid electron conductor materials (electron mediator), such as Ag, Au, Cu nanoparticles, CNTs, and graphene [109,111,112,113]. These electron mediators not only effectively transfer the photogenerated electrons but also widen the visible-light absorption and improve the stability of photocatalysts [114]. Moreover, all-solid-state Z-scheme heterojunction photocatalysts can be applied in liquid as well as gas phases. Under light irradiation, the transfer of photogenerated charge carriers between two photocatalysts takes place through the solid electron mediator used as a charge carrier transfer bridge. Unfortunately, the synthesis process of this photocatalyst may not be cost-effective due to the use of expensive noble metals, and problems like the shielding effect may be caused by noble metal nanoparticles. Figure 6b depicts a schematic of the charge-transfer process in an all-solid-state Z-scheme heterojunction photocatalyst.

#### 5.1.3. Direct Z-Scheme Heterojunction Photocatalysts

The concept of direct Z-scheme heterojunction photocatalysts was first reported by Yu et al. in 2013 [115]. In this system, two semiconductor photocatalysts (OP and RP) are in close contact without a charge carrier transfer mediator. The migration pathway of photogenerated charge carriers in a direct Z-scheme heterojunction resembles the letter “Z”, from which it gets its name. Upon light irradiation, the photogenerated electrons in the CB of the OP recombine with the photogenerated holes in the VB of the RP. Therefore, the photogenerated electrons in the CB of the RP and the photogenerated holes in the VB of the OP become spatially separated and can be involved in reduction and oxidation processes, respectively. Also, it is noteworthy that the induced electric field significantly enhances the separation and transfer processes of photogenerated charge carriers [116,117]. As a result, the redox ability of a direct Z-scheme heterojunction photocatalyst is improved, and its photocatalytic performance is accelerated [118]. A schematic of the charge-transfer process in a direct Z-scheme heterojunction photocatalyst is shown in Figure 6c.

#### 5.1.4. Double Z-Scheme Heterojunction Photocatalysts

However, the fabrication of a direct Z-scheme heterojunction photocatalytic system is considered a promising strategy to obtain enhanced photocatalytic performance; still, there are some drawbacks in this system, which are regarded as hindering factors for further improvements in photocatalytic activity. As is known, all Z-scheme heterojunction photocatalysts offer oxidation and reduction surfaces with a fixed ratio of 1:1. Therefore, some of the photogenerated electrons and holes may be wasted during their migration to a higher VB and a lower CB. For the purpose of preventing this problem, the concept of the double Z-scheme heterojunction photocatalytic system was introduced. Double Z-scheme heterojunction photocatalysts can be fabricated by combining different semiconductor photocatalysts (generally two OPs and one RP) and possess some additional advantages compared to other Z-scheme heterojunctions, which include enhanced light-harvesting ability; utilization of the whole solar spectrum; the low transfer resistance of photogenerated charge carriers to minimize their recombination; expanded oxidation and reduction surfaces; and appropriate band-gap positions for enhancing interfacial electron transfer and increasing their lifetime [119]. In a double Z-scheme heterojunction photocatalyst, when subjected to light irradiation, the photogenerated electrons in the CB of both OPs move to the VB of the RP and recombine with the photogenerated holes. Thus, the photogenerated electrons in the CB of the RP and holes in the VB of both OPs are effectively separated and take part in reduction and oxidation reactions, respectively. A schematic of the charge-transfer process in a double Z-scheme heterojunction photocatalyst is shown in Figure 6d.

### 5.2. Step-Scheme (S-Scheme) Heterojunction Photocatalysts

Since Z-scheme heterojunctions are a big family of heterojunction photocatalysts, numerous efforts have been made to design and fabricate high-performance Z-scheme heterojunction photocatalysts. As a result, remarkable achievements have also been made, but direct Z-scheme photocatalysts suffer the consequences of the shortcomings of traditional and all-solid-state heterojunction photocatalysts [120,121]. In order to eliminate these shortcomings, a brand-new concept of the S-scheme heterojunction photocatalytic system was introduced in 2019. The credit for introducing this concept, which can describe the photocatalytic mechanism more clearly, goes to Yu and coworkers [122]. Since then, massive efforts have been dedicated to the development of noble and even more efficient S-scheme heterojunction photocatalysts. An S-scheme heterojunction is composed of two different n-type semiconductor photocatalysts, namely, an OP and an RP. Due to the difference in Fermi levels, when the OP comes in contact with the RP, the electrons in the RP spontaneously diffuse to the OP until the Fermi levels are aligned to the same level, which leads to the upward and downward shifts of the Fermi levels of the OP and RP, respectively. As a result, an internal electric field is generated, and band bending occurs at the interface, which enhances the separation of photogenerated charge carriers [123,124]. 

Under light irradiation, the charge transfer in an S-scheme heterojunction resembles a “step”, hence its name, at the macroscopic level since the photogenerated electrons and holes move one step to the more negative CB and the more positive VB, respectively. Moreover, during light irradiation, the electrons are excited in both the OP and RP, and then the electrons in the CB of the OP move toward the VB of the RP and recombine with holes due to the coulombic force of attraction. Consequently, the useless electrons and holes are eliminated, while the powerful photogenerated electrons in the CB of the RP and holes in the VB of the OP remain well separated. This type of movement of charge carriers is similar to the letter “N” at the microscopic level. In this way, the resulting S-scheme heterojunction photocatalyst promotes the separation of photogenerated charge carriers through an internal electric field and band bending and engages them in photocatalytic redox reactions [125,126]. In summary, three factors, i.e., the internal electric field, band bending, and the coulombic force of attraction, play important roles as driving forces of charge carriers in an S-scheme heterojunction photocatalyst for the recombination of electrons in the CB of the OP and holes in the VB of the RP and the preservation of separated electrons in the CB of the RP and holes in the VB of the OP. Figure 6e displays a schematic illustration of an S-scheme heterojunction photocatalyst, along with the charge-transfer process.

## 6. Formation of g-C_3_N_4_/ZnO-Based Z-Scheme Heterojunction Photocatalysts

Motivated by the various aforementioned advantages of different types of Z-scheme heterojunctions, spectacular attention has been drawn to the design and fabrication of g-C_3_N_4_/ZnO-based Z-scheme heterojunction photocatalysts to obtain excellent photocatalytic results.

### 6.1. Formation of g-C_3_N_4_/ZnO-Based All-Solid-State Z-Scheme Heterojunction Photocatalysts

Many studies have demonstrated that ZnO and g-C_3_N_4_ are decent candidates for the fabrication of heterojunction photocatalysts, having an improved separation efficiency of electron–hole pairs and an increased redox ability of charge carriers. Unfortunately, the construction of a ZnO-and-g-C_3_N_4_ heterojunction with an intimate interface is still difficult due to their different crystal structures and lattice parameters. Therefore, Di et al. introduced a novel strategy to synthesize a highly efficient heterojunction photocatalyst of g-C_3_N_4_ and ZnO by adopting amorphous Fe_2_O_3_ as an electron mediator and connecting bridge between g-C_3_N_4_ and ZnO. Different heterojunction photocatalysts were prepared with varying mass ratios of ZnO/Fe_2_O_3_ to g-C_3_N_4_ (x, given in wt%) in a hydrothermal and low-temperature calcination process [127]. The photocatalytic activity of as-prepared samples was evaluated by degrading sulfonamides in an aqueous solution under visible-light irradiation at different pH values. Experimental results clearly demonstrated the degradation order as ZnO < ZnO/Fe_2_O_3_ < g-C_3_N_4_ < ZnO/g-C_3_N_4_-10% < ZnO/Fe_2_O_3_/g-C_3_N_4_-1% < ZnO/Fe_2_O_3_/g-C_3_N_4_-20% < ZnO/Fe_2_O_3_/g-C_3_N_4_-5% < ZnO/Fe_2_O_3_/g-C_3_N_4_-10%. Interestingly, ZnO/Fe_2_O_3_/g-C_3_N_4_-5% and ZnO/Fe_2_O_3_/g-C_3_N_4_-10% samples showed almost equal efficiency, which might be ascribed to the competition of ZnO/Fe_2_O_3_ and g-C_3_N_4_ to absorb visible light, with the excess loading of ZnO/Fe_2_O_3_ restraining the generation of ROS. Hence, a ZnO/Fe_2_O_3_/g-C_3_N_4_-10% heterojunction was screened out as the best-performing photocatalyst for subsequent experiments at pH 7.2. The higher photocatalytic performance of ZnO/Fe_2_O_3_/g-C_3_N_4_ heterojunctions compared to that of ZnO, ZnO/Fe_2_O_3_, g-C_3_N_4_, and ZnO/g-C_3_N_4_-10% is ascribed to the major role of amorphous Fe_2_O_3_. Amorphous Fe_2_O_3_ used as an electron mediator and connecting bridge between ZnO and g-C_3_N_4_ could increase the charge transfer, accelerate the charge separation, and modulate the band energy structures to contribute to the photocatalytic performance. The reusability of the ZnO/Fe_2_O_3_/g-C_3_N_4_-10% heterojunction was evaluated by conducting cycling tests under similar conditions, where it was capable of efficiently degrading sulfonamides in five cycles. Moreover, when the degradation process was conducted at higher pH (10.97), electrostatic repulsion between negatively charged sulfonamide (SMZ^−^), exhibiting a zwitterion structure, and the negatively charged surface of the ZnO/Fe_2_O_3_/g-C_3_N_4_-10% heterojunction hindered their contact, leading to a decrease in degradation rate. On the other hand, at low pH (3.03), the increased concentration of H^−^ ions consumed O_2_^•−^ radicals, thereby decreasing the degradation rate of sulfonamides.

**Figure 7:**
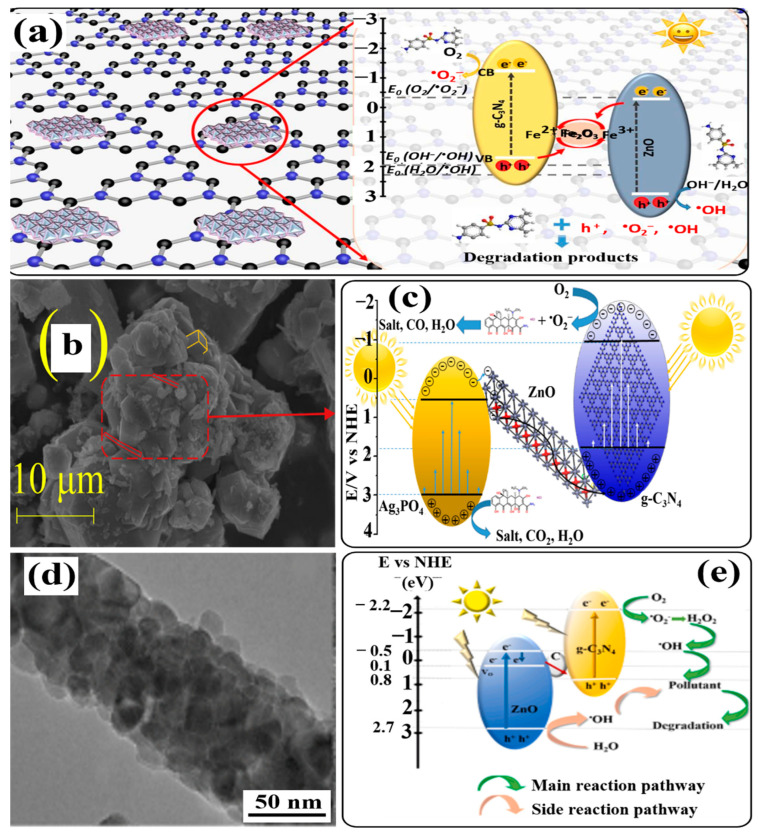
(**a**) Schematic illustration of the plausible photocatalytic mechanism of ZnO/Fe_2_O_3_/g-C_3_N_4_ heterojunction under visible-light irradiation, (**b**) SEM image of ACZ, (**c**) photocatalytic reaction mechanism diagram of Ag_3_PO_4_/g-C_3_N_4_/ZnO composite, (**d**) TEM image of typical ZnO/C/g-C_3_N_4_ nanofiber at 120,000 magnification, and (**e**) schematic diagram of the two possible proposed mechanisms of charge transfer, as well as the intermediate reactions for degradation of the pollutant over the ZnO/C/g-C_3_N_4_ Z-scheme system with carbon at the interface between ZnO and g-C_3_N_4_. Reprinted from Refs. [127,130,136] with permission from Elsevier.

Based on the analysis, a Z-scheme charge-transfer mechanism has been proposed for the ZnO/Fe_2_O_3_/g-C_3_N_4_-10% heterojunction. During light irradiation, both ZnO/Fe_2_O_3_ and g-C_3_N_4_ undergo excitation and generate electron–hole pairs. The photogenerated electrons in the CB of ZnO are captured by Fe^3+^ in amorphous Fe_2_O_3_ and reduce it to Fe^2+^. Meanwhile, the photogenerated holes in the VB of g-C_3_N_4_ oxidize Fe^2+^ back to Fe^3+^. Also, Fe^2+^ can be oxidized by dissolved O_2_ to produce Fe^3+^ and O_2_^•−^ radicals. In this way, amorphous Fe_2_O_3_ acts as a redox center and shows dual functions: first, as an electron mediator to promote the separation of electrons and holes effectively, and second, as a generator of O_2_^•−^ radicals for subsequent redox reactions. The photogenerated electrons accumulated in the CB of g-C_3_N_4_ (RP) react with dissolved O_2_ to generate O_2_^•−^ and OH^•^ radicals since the conduction band potential (E_CB_) (−1.19 eV) of g-C_3_N_4_ is more negative than the standard redox potential (E^°^) ((O_2_/O_2_^•−^), −0.33 eV vs. NHE). Likewise, the photogenerated holes in the VB of ZnO/Fe_2_O_3_ (E_VB_, +2.27 eV) enable the oxidation of H_2_O/OH^−^ to OH^•^ radicals. Furthermore, radical-quenching experiments in the presence of different scavengers indicated that holes and O_2_^•−^ radicals were the main ROS responsible for sulfonamide degradation, but the OH^•^ radical was not the dominant ROS, although it contributed to the photocatalytic process. A schematic illustration of the plausible photocatalytic mechanism in the ZnO/Fe_2_O_3_/g-C_3_N_4_ heterojunction is shown in Figure 7a. In another work, Zhang et al. first reported the facile synthesis of a Z-scheme g-C_3_N_4_/ZnO@graphene aerogel (g-C_3_N_4_/ZnO@GA) heterojunction by hydrothermal assembly combined with freeze-drying [128]. The thus-obtained g-C_3_N_4_/ZnO@GA heterojunction photocatalyst showed excellent performance in the photodegradation of various pollutants, like rhodamine B (Rh B), methyl orange (MO), methyl violet (MV), and methylene blue (MB), under UV-light and visible-light irradiation. In this process, GA functioned as the electron mediator and accelerator of the charge carriers’ transportation. Bai and coworkers also reported a facile and feasible method to construct a carbon nitride-nested tubular Z-scheme ZnO/g-C_3_N_4_/RGO system by direct heating method [129]. The photodegradation properties of the ZnO/g-C_3_N_4_/RGO system were investigated by degrading deoxynivalenol under visible- and UV-light irradiation. The findings of this work indicated that RGO could serve as a dual electron mediator as well as facilitate the localization of graphene in the photocatalytic process.

Many studies have reported that coupling g-C_3_N_4_ with Ag_3_PO_4_ could be the best alternative for the construction of heterojunction photocatalysts with a higher charge separation ability at the heterojunction interface since both the semiconductor photocatalysts possess suitable CB and VB levels. Owing to this, Zhu et al. prepared a ternary Ag_3_PO_4_/g-C_3_N_4_/ZnO photocatalyst by a facile ultrasound-assisted precipitation method [130]. Different samples were prepared for coupling g-C_3_N_4_/ZnO units (with different mass fraction %) and different % of Ag_3_PO_4_ units. Scanning electron microscopic images (SEM) showed the existence of cubic Ag_3_PO_4_, dendritic and petal-like g-C_3_N_4_, and variously sized ZnO particles dispersed on the composite (Figure 7b). The photocatalytic properties of the prepared samples were evaluated by the removal of tetracycline hydrochloride (TC) under visible and sunlight irradiation. The data obtained from the degradation experiments indicated that the sample 80-ACZ (obtained from g-C_3_N_4_/ZnO units with a 90% mass fraction (90-CZ) and 80% Ag_3_PO_4_ units) maintained higher photocatalytic activity with good photocatalytic reproducibility for up to four successive cycles. This is attributed to the synergistic effect of Ag_3_PO_4_, g-C_3_N_4_, and ZnO. An increase in ZnO content to 90% leads to an increase in light absorption capacity due to the attraction of Zn to highly electronegative N and O of TC. With the addition of g-C_3_N_4_, the light response range is also extended because of its narrow band gap and the strong conductivity of ZnO. In addition, with an increase in the Ag_3_PO_4_ content to 80%, the degradation efficiency of the 80-ACZ photocatalyst was increased, which might be attributed to the narrow band gap of Ag_3_PO_4_ and the overall increase in the visible-light response of the photocatalyst.

An investigation of the effective role of ROS involved in the degradation process was performed in the presence of different scavengers. The experimental findings suggested that holes and O_2_^•−^ radicals played a major role, while OH^−^ radicals showed comparatively little effect. The probable photocatalytic mechanism of the 80-ZCN photocatalyst was deduced on the basis of the calculated band positions of Ag_3_PO_4_, g-C_3_N_4_, and ZnO. According to this study, the band gaps of Ag_3_PO_4_, g-C_3_N_4_, and ZnO are 2.45, 2.76, and 3.12 eV, respectively, and their VB positions are +0.5, −0.99, and −0.52 eV (vs. NHE), respectively. Similarly, their CB positions were calculated to be +2.95, +1.75, and +2.6 eV for Ag_3_PO_4_, g-C_3_N_4_, and ZnO, respectively. When photocatalysts are subjected to light irradiation, electrons are excited to the CB, leaving holes in their respective VB. The oxidation potential of O_2_^•−^ is −0.33 eV, which is more positive than the CBs of g-C_3_N_4_ and ZnO and more negative than the CB of Ag_3_PO_4_. Therefore, photogenerated electrons accumulated in the CB of Ag_3_PO_4_ recombine with the photogenerated holes in the VB of g-C_3_N_4_ through ZnO, which acts as an electronic conductor. The photogenerated holes accumulated in the VB of Ag_3_PO_4_ cannot oxidize H_2_O or OH^−^ since the VB is higher than the oxidation potential of OH^•^. Instead, holes are directly involved in the degradation process of TC. On the other hand, photogenerated electrons accumulated in the CB of g-C_3_N_4_ react with dissolved oxygen to generate O_2_^•−^ radicals. Finally, a Z-scheme heterojunction photocatalytic mechanism is proposed in Figure 7c.

Electrospinning is a versatile and cost-effective method for the fabrication of nanofibers as one-dimensional materials. It is well known that electrospun nanofibers have various advantages, including continuous morphology, a large aspect ratio, high porosity, flexibility, and interconnectivity [131,132]. Hence fabrication of different types of electrospun nanofibers (like polymeric fibers, composites, carbon, ceramics) has attracted intense attention as new-generation materials for emerging applications [6,133,134,135]. Considering the benefits of electrospun nanofibers, Ramkrishna and coworkers prepared semiconductor nanofibers with carbonaceous materials in the recent past. In this work, they synthesized composite nanofibers of ZnO/C/g-C_3_N_4_ (with different wt.% of g-C_3_N_4_) using the electrospinning method, followed by annealing under a N_2_ atmosphere at 460 °C [136]. From transmission electron microscopy (TEM) analyses, the diameter of as-prepared nanofibers was estimated to be 70 ± 6 nm (Figure 7d). Additionally, the electron spin resonance (ESR) technique and X-ray photoelectron spectroscopy (XPS) indicated the presence of oxygen vacancies in the ZnO lattice and carbonaceous species in the ZnO/C/g-C_3_N_4_ nanofibers. It was reported that the annealing of precursor nanofibers under an inert gas like N_2_ could generate oxygen vacancies in the ZnO lattice.

The photocatalytic activity of ZnO/C/(x wt%) g-C_3_N_4_ nanofibers was evaluated by the photodegradation of MB dye in an aqueous solution under simulated sunlight illumination. The experimental results revealed that carbonaceous ZnO/C/g-C_3_N_4_ nanofibers exhibited higher photocatalytic activity than ZnO and g-C_3_N_4_, which is attributed to the efficient electron–hole separation of the heterojunctions. However, the higher efficiency of ZnO/C/(25 wt%) g-C_3_N_4_ nanofibers compared to other nanofibers containing different wt% of g-C_3_N_4_ was not explained. On the basis of calculated band positions, the charge-transfer mechanism in the ZnO/C/(25 wt%) g-C_3_N_4_ heterojunction has been proposed. The VB and CB positions of g-C_3_N_4_ were calculated to be about +0.8 and −2.2 eV, respectively, while the CB energy level of ZnO was −0.5 eV, and the oxygen vacancy state was +0.1 eV. Based on these data, the charge-transfer mechanism in the ZnO/C/(25 wt%) g-C_3_N_4_ heterojunction is shown in Figure 7e. Under light irradiation, the holes in the VB of g-C_3_N_4_ cannot oxidize H_2_O to OH^•^ radicals since the VB potential of g-C_3_N_4_ (+0.8 eV vs. NHE) is less positive than the redox potential (E°) of (OH^•^/OH^−^) (+1.99 eV vs. NHE, pH = 7). On the other hand, the trapped electrons in oxygen vacancies of ZnO cannot reduce O_2_ to O_2_^•−^ [E° (O_2_/O_2_^•−^)] (−0.33 eV vs. NHE, pH = 7). Hence, solid-state Z-scheme charge transfer occurs in the heterojunction, in which carbonaceous species act as electron mediators to facilitate the recombination of photogenerated electrons in the CB of ZnO and holes in the VB of g-C_3_N_4_. As a result, the photogenerated electrons and holes accumulate in the CB of g-C_3_N_4_ (RP) and the VB of ZnO (OP), respectively. The thus-separated electrons and holes with high redox potential reacted with dissolved O_2_ and H_2_O or OH^−^ ions, respectively, to produce ROS (O_2_^•−^ and OH^•^), which participated in the MB dye degradation process utilizing ZnO/C/g-C_3_N_4_ nanofibers. However, holes in the VB of ZnO did not play an active role in photodegradation, and OH^•^ radicals produced from holes remained inactive. But active-species-trapping experiments in the presence of various scavengers showed that both O_2_^•−^ and OH^•^ radicals were vital ROS. Therefore, it is supposed that O_2_^•−^ radicals could produce OH^•^ radicals from adsorbed H^+^ ions. The corresponding reactions are shown below:

(10) $$O_{2}^{•-}+e^{-}+{2H}^{+}\overset{}{\to }H_{2}O_{2}$$

(11) $$H_{2}O_{2}+e^{-}+H^{+}\overset{}{\to }{OH}^{•}+H_{2}O$$

To enhance the photocatalytic activity of a Z-scheme heterojunction photocatalyst via metal doping, Bajiri et al. prepared a Cu-doped ZnO/Cu/g-C_3_N_4_ heterostructure using a calcination–hydrothermal method in which Cu plays three roles: as a dopant, it reduces the band gap of ZnO; as an electron mediator, it facilitates charge transport; and as a co-catalyst, it enhances the catalytic activity [137]. The as-prepared Cu-doped ZnO/Cu/g-C_3_N_4_ heterostructure degraded MB and Rh B dyes more effectively than single and binary photocatalysts under sunlight irradiation.

**Figure 8:**
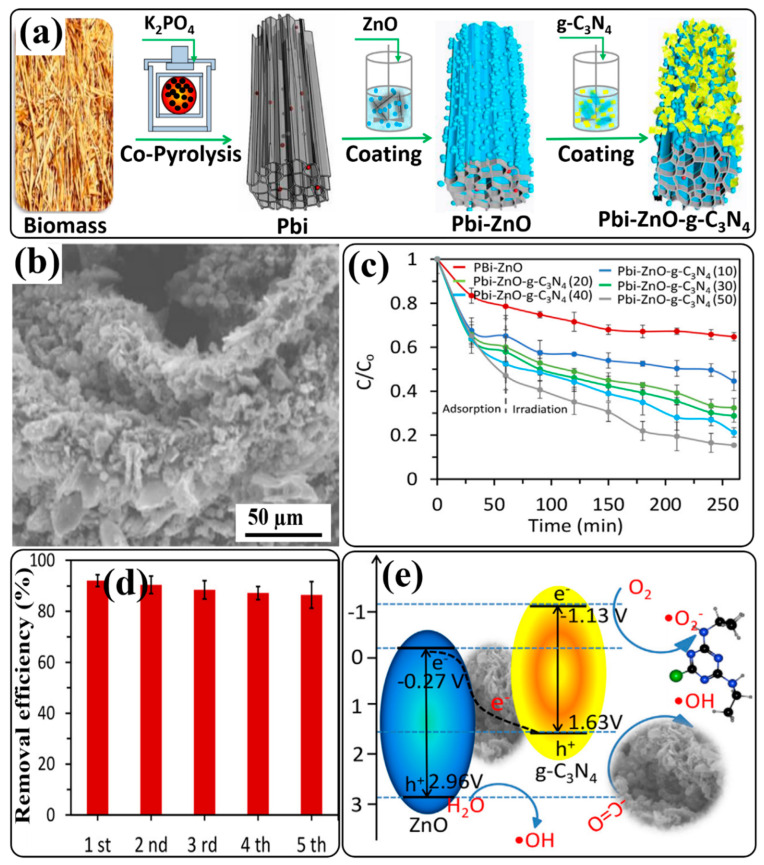
Schematic illustration of the synthetic processes of Pbi-ZnO-g-C_3_N_4_ (**a**), SEM image of Pbi-ZnO-g-C_3_N_4_ (**b**), the photocatalytic degradation efficiency of atrazine by Pbi-ZnO and Pbi-ZnO-g-C_3_N_4_ under simulated sunlight (**c**), atrazine degradation over Pbi-ZnO-g-C_3_N_4_ during five recycling tests (**d**), and proposed mechanisms for the formation of reactive oxidative species during biochar- and Pbi-ZnO-g-C_3_N_4_-based photocatalytic processes (**e**). Reprinted from Ref. [138] with permission from Elsevier.

Recently, an inspiring work was reported by Yu and coworkers. They synthesized biomass-derived core–shell P-laden biochar/ZnO/g-C_3_N_4_ (Pbi-ZnO-g-C_3_N_4_) heterojunction composites with varied mass percentages of g-C_3_N_4_ to Pbi-ZnO by thermal polymerization, copyrolysis, and annealing under a N_2_ atmosphere [138]. The synthesis process of the Pbi-ZnO-g-C_3_N_4_ heterojunction composite is shown schematically in Figure 8a. SEM images revealed the distribution of ZnO nanoparticles and curved-edge g-C_3_N_4_ nanosheets on the surface of tubular carbon walls (Figure 8b). Photodegradation experiments under simulated sunlight showed that the Pbi-ZnO-g-C_3_N_4_ composite with 50 mass% of g-C_3_N_4_ to ZnO [(Pbi-ZnO-g-C_3_N_4_)50] can achieve enhanced atrazine degradation efficiency (Figure 8c), which is attributed to the higher percentage of g-C_3_N_4_, which can lead to the higher utilization of visible light and the formation of ROS. Figure 8d shows that the atrazine degradation efficiency of Pbi-ZnO-g-C_3_N_4_ was very stable for five cycles. The degradation efficiency was decreased by only 5.6% after five cycles. Radical-trapping experiments suggested that both O_2_^•−^ and OH^•^ radicals are responsible for atrazine degradation. The proposed mechanism for the formation of ROS and the photodegradation of atrazine is shown in Figure 8e. During light irradiation of the Pbi-ZnO-g-C_3_N_4_ composite, electrons are transferred to the CBs of ZnO and g-C_3_N_4_ from their respective VBs, along with the generation of holes. The presence of biochar in the Pbi-ZnO-g-C_3_N_4_ heterojunction not only improved the light absorption but also served as the charge transmission bridge between ZnO and g-C_3_N_4_ due to its excellent electrical conductivity. Therefore, the photogenerated electrons in the CB of ZnO and holes in the VB of g-C_3_N_4_ are recombined via the biochar bridge, which enhanced the separation of photogenerated holes in the VB of ZnO and electrons in the CB of g-C_3_N_4_. A Z-scheme heterojunction charge-transfer process occurred between ZnO and g-C_3_N_4_. Since the CB position of g-C_3_N_4_ is lower than the calculated reduction potential of O_2_^•−^/O_2_ (−0.33 eV), the dissolved oxygen can be reduced to O_2_^•−^ radicals, while the calculated VB position of ZnO is greater than the oxidation potential of H_2_O/OH^−^ (+2.40 eV), so water can be oxidized to OH^•^ radicals. Also, photogenerated holes in the VB of ZnO can oxidize atrazine directly. Finally, it is believed that this synthesis strategy may open a new avenue for the utilization of biomass in a meaningful way to alleviate environmental issues emerging from agricultural use.

Similarly, another recent work regarding an all-solid-state Z-scheme heterojunction photocatalyst was reported by Wu et al. in 2022 [139]. They synthesized a NiCo alloy/ZnO/g-C_3_N_4_ magnetic nanocomposite for the photodegradation of oxytetracycline and tetracycline under visible-light irradiation. In the NiCo/ZnO/g-C_3_N_4_ magnetic nanocomposite, NiCo alloy acted as an electron conduction bridge between ZnO and g-C_3_N_4_ to further enhance the separation efficiency, thereby enhancing the photodegradation rate. Moreover, Santhini et al. utilized a simple and cost-effective calcination method to prepare highly efficient g-C_3_N_4_/ZnO/Fe_2_O_3_ ternary composite photocatalysts [140]. The g-C_3_N_4_/ZnO/Fe_2_O_3_ composite was revealed to have enhanced MB dye degradation efficiency (94% in 120 min) and photostability under visible-light irradiation. There was no significant change in the photodegradation of MB dye even after five consecutive cycles. It is inferred that the superior photocatalytic performance of the composite is associated with the effective charge separation ability via an all-solid-state Z-scheme mechanism in which Fe_2_O_3_ significantly acts as the redox center to promote photogenerated electron–hole pair separation and the generation of ROS (mainly O_2_^•−^ and OH^•^ radicals). For the convenience of readers, different reports based on all-solid-state Z-scheme heterojunction photocatalysts of g-C_3_N_4_ coupled with ZnO are listed in Table 1.

### 6.2. Formation of g-C_3_N_4_/ZnO-Based Direct Z-Scheme Heterojunction Photocatalysts

Various reports, including recent processes, on the fabrication of g-C_3_N_4_/ZnO-based direct Z-scheme heterojunction photocatalysts in different categories are discussed in the following sub-sections.

#### 6.2.1. Formation of Binary g-C_3_N_4_/ZnO-Based Direct Z-Scheme Heterojunction Photocatalysts

In binary g-C_3_N_4_/ZnO-based direct Z-scheme heterojunction photocatalysts, g-C_3_N_4_ and ZnO semiconductor photocatalysts in their different morphological forms are coupled together via different strategies. In these photocatalysts, oxidation and reduction reactions occur at ZnO and g-C_3_N_4_, respectively. The migration of photogenerated charge carriers during the photodegradation process in such photocatalysts proceeds on the basis of the CB and VB band positions of g-C_3_N_4_ and ZnO. In particular, the photogenerated electrons in the CB of ZnO directly recombine with the photogenerated holes in the VB of g-C_3_N_4_, resulting in the effective separation of photogenerated electrons and holes in the CB of g-C_3_N_4_ and the VB of ZnO, respectively. A few years ago, Kang’s group presented a synthesis strategy for a binary g-C_3_N_4_/ZnO-based direct Z-scheme heterojunction photocatalyst. They synthesized mesoporous ZnO nano-triangles@ g-C_3_N_4_ nanofoils (ZnO-nt@g-C_3_N_4_) with different concentrations of g-C_3_N_4_. The photocatalyst ZnO-nt@g-C_3_N_4_ (20%) exhibited 100% degradation of Rh B dye in 60 min under solar-light irradiation [141]. Next, Wang et al. proposed an oxygen-defect-mediated Z-scheme mechanism in exfoliated g-C_3_N_4_ nanosheets coupled with oxygen-defective ZnO (OD-ZnO) nanorods, i.e., CN/OD-ZnO heterojunctions having different wt% of g-C_3_N_4_ [142]. The heterojunction containing 10 wt% g-C_3_N_4_ (CN-10/OD-ZnO) exhibited an increased visible-light absorption ability and enhanced (90%) photodegradation of 4-chlorophenol (4-CP) in 60 min. Radical-trapping experiments confirmed that OH^•^ radicals played the most critical role in the final complete mineralization.

**Figure 9:**
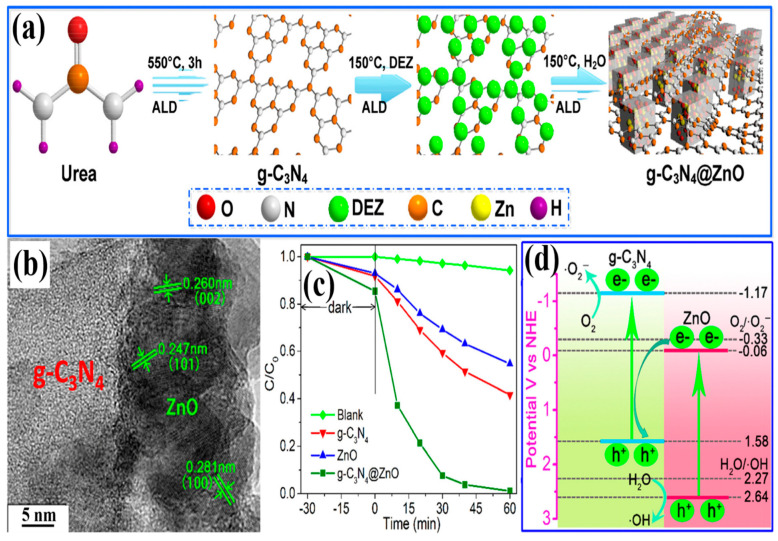
(**a**) Schematic illustration of fabrication process of ALD-based g-C_3_N_4_@ZnO photocatalyst, (**b**) HRTEM image of a randomly selected position from g-C_3_N_4_@ZnO sample, (**c**) photocatalytic degradation of cephalexin by g-C_3_N_4_, g-C_3_N_4_@ZnO, and ZnO under simulated sunlight irradiation, and (**d**) schematic illustration of the mechanism of enhanced photocatalytic activity. Reprinted from Ref. [143] with permission from Elsevier.

A newly designed 2D/3D direct Z-scheme heterostructure was reported by Tian and coworkers. For the first time, they synthesized a binary direct Z-scheme heterostructure of 2D g-C_3_N_4_ and 3D ZnO nanosheets in a controlled manner using atomic layer deposition (ALD) [143]. The schematic illustration of the fabrication process of the ALD-based g-C_3_N_4_@ZnO photocatalyst is shown in Figure 9a. HRTEM image of the novel 2D/3D g-C_3_N_4_@ZnO nanosheet heterostructure clearly displayed the interface between g-C_3_N_4_ nanosheets and ZnO nanolayers, revealing the firm connection between the two components. The lattice fringe with d-spacing of 0.260, 0.247, and 0.281 nm was attributed to the (002), (101), and (100) planes of hexagonal wurtzite ZnO, respectively, indicating good crystallinity (Figure 9b). To estimate the photocatalytic performance of the 2D/3D g-C_3_N_4_@ZnO nanosheet heterostructure, photodegradation experiments with cephalexin were carried out under simulated sunlight (Figure 9c). Compared to g-C_3_N_4_ and ZnO, the 2D/3D g-C_3_N_4_@ZnO nanosheet heterostructure exhibited enhanced light absorption properties and photodegradation capability (98.8% in 60 min), which might have benefited from the efficient charge transfer and increased surface area, which could provide more adsorption sites for cephalexin adhesion. Additionally, the higher zeta potential of the heterostructure surface contributed to the strong attraction of cephalexin molecules for photocatalysts. Consecutive photocatalytic degradation of cephalexin with 2D/3D g-C_3_N_4_@ZnO exhibited a similar trend over five cycles. However, only a slight decline (1.9%) in removal efficiency was observed after five degradation cycles.

Based on the generation and transformation behavior of photogenerated charge carriers in the 2D/3D g-C_3_N_4_@ZnO heterostructure, a photodegradation mechanism was proposed. When simulated sunlight was irradiated on the photocatalyst, excited electrons from the VBs of g-C_3_N_4_ and ZnO were transferred to their respective CBs, leaving behind holes. The photogenerated holes in the VB of g-C_3_N_4_ cannot generate OH^•^ radicals from H_2_O or OH^−^ ions since the VB edge potential of g-C_3_N_4_ is less positive (+1.58 V) than the standard redox potential of H_2_O/OH^•^ (+2.27 V), but due to the more positive VB edge potential (+ 2.64 V) of ZnO, OH^•^ radicals were generated by holes in the VB of ZnO. In addition, dissolved O_2_ can be oxidized to O_2_^•−^ radicals by photogenerated electrons only in the CB of g-C_3_N_4_ due to its more negative CB edge potential (−1.17 V) compared to the standard redox potential of O_2_/O_2_^•−^ (−0.33 V). Instead, the photogenerated electrons and holes in the CB of ZnO and the VB of g-C_3_N_4_, respectively, recombined with the potential difference (+1.64 V) as a driving force, resulting in the effective separation of photogenerated electrons in the CB of g-C_3_N_4_ and holes in the VB of ZnO, respectively. Hence, a direct Z-scheme charge-transfer system was determined in the 2D/3D g-C_3_N_4_@ZnO nanosheet heterostructure photocatalyst (Figure 9d). Furthermore, radical-trapping experiments using different scavengers confirmed that holes and OH^•^ radicals were the dominant ROS for cephalexin photodegradation.

To understand the effect of the annealing temperature on the interaction between g-C_3_N_4_ and ZnO in the g-C_3_N_4_/ZnO binary composite, Jung et al. prepared g-C_3_N_4_/ZnO composites at various annealing temperatures [144]. HRTEM images depicted a strong interaction between g-C_3_N_4_ nanosheets and ZnO nanoparticles. Experimental findings suggested that the composite prepared at a higher (500 °C) annealing temperature (g-C_3_N_4_/ZnO-500) could show higher photocatalytic ability for degrading MB dye under visible-light irradiation. It is believed that the g-C_3_N_4_/ZnO-500 composite contains strong interactions between the two components, which not only decreases the electron density of ZnO nanoparticles but also significantly decreases the recombination of photogenerated electrons and holes. Similarly, other binary g-C_3_N_4_/ZnO Z-scheme heterojunction photocatalysts were prepared via different synthesis approaches for the investigation of the enhanced photodegradation performance for MB dye under visible-light/simulated sunlight irradiation [145,146]. Fang et al. fabricated a 0D/2D hybrid to utilize the synergistic coupling effect of different dimensionalities in the spatial separation of photogenerated charge carriers. They immobilized 0D g-C_3_N_4_ quantum dots (QDs) onto 2D ZnO nanosheets with oxygen vacancies (OVs) [147]. The as-prepared CNQDs/OV-ZnO heterojunction exhibited higher photodegradation activity for degrading MB dye and bisphenol A (BPA) under visible-light irradiation.

**Figure 10:**
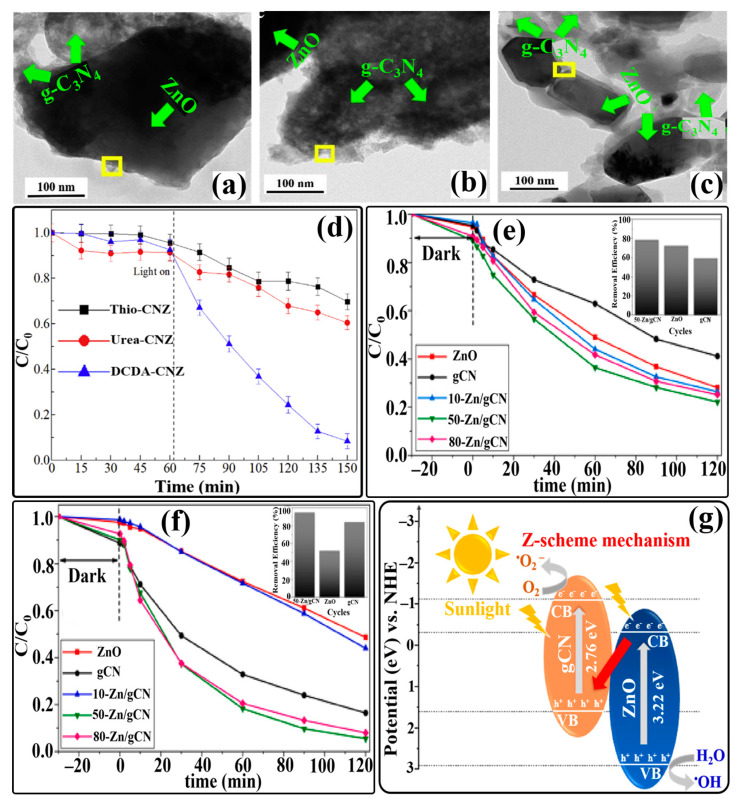
HRTEM images of (**a**) Thio-CNZ, (**b**) Urea-CNZ, and (**c**) DCDA-CNZ; (**d**) adsorption in the dark and photocatalytic degradation in the visible-light irradiation of MB over CNZ composite photocatalysts. Photocatalytic degradation curves of (**e**) CFZ and (**f**) RB5 by ZnO, gCN, and their heterostructures. Diagram illustrating the possible charge separation on 50-Zn/gCN through the Z-scheme mechanism (**g**). Reprinted from Refs. [148,157] with permission from Elsevier.

As introduced in Section 3, the major precursors of g-C_3_N_4_ are urea, thiourea, and dicyandiamide (DCDA). However, the morphological structures of the resultant heterojunctions of g-C_3_N_4_ derived from these precursors remain different. Hence, to understand the interactions between g-C_3_N_4_ precursors and ZnO and the morphological structures of the resultant g-C_3_N_4_/ZnO (CNZ) composites, namely, Urea-CNZ, Thio-CNZ, and DCDA-CNZ composites, Jung et al. introduced a synthesis strategy [148]. HRTEM analysis revealed different interfaces between g-C_3_N_4_ and ZnO in Urea-CNZ, Thio-CNZ, and DCDA-CNZ (Figure 10a–c). A thin layer of amorphous g-C_3_N_4_ surrounding ZnO nanoparticles was observed in the DCDA-CNZ composite, thereby forming a core–shell structure, while g-C_3_N_4_ and ZnO in Urea-CNZ and Thio-CNZ composites were segregated from each other, resulting in weak interactions between g-C_3_N_4_ and ZnO nanoparticles. Furthermore, the morphologies of Urea/Thio-ZnO composites were different from that of the DCDA-CNZ composite. These composites exhibited porous structures. The weak interaction between ZnO and Urea/Thio caused the self-polymerization of the precursors in thermal polymerization steps with the release of gas molecules, such as NH_3_, H_2_O, CO_2_, CS_2_, and H_2_S, resulting in porous g-C_3_N_4_. But the release of these gas molecules did not occur in the thermal polymerization process, which did not interrupt the interaction between g-C_3_N_4_ and CNZ. To examine the photocatalytic performance of as-prepared Urea-CNZ, Thio-CNZ, and DCDA-CNZ composites, photocatalytic degradation tests of MB dye were performed under visible-light irradiation. Among these composites, DCDA-CNZ exhibited the highest degradation efficiency, as shown in Figure 10d. It is claimed that the highest efficiency of the DCDA-CNZ composite is attributed to its core–shell morphology owing to the interaction between g-C_3_N_4_ and ZnO nanoparticles. Finally, based on the band gap measurement, a direct Z-scheme charge-transfer mechanism has been proposed in which charges activated by photons are transferred from ZnO to g-C_3_N_4_. It would have been even better if a detailed explanation of ROS generation and a schematic illustration of the Z-scheme charge-transfer pathway, including band-gap positions in the DCDA-CNZ composite, had been included.

A binary g-C_3_N_4_/ZnO heterojunction photocatalyst was obtained through low-cost in situ fabrication, co-melting crystallization, and a calcination process for the removal of organic pollutants present in wastewater [149,150,151]. Due to their unique properties, like a large surface area and good electrical and optical properties, exfoliated g-C_3_N_4_ nanosheets have attracted intense attention from researchers for the construction of g-C_3_N_4_-based heterojunction photocatalysts. So, inspired by exfoliated graphene from bulk graphite, Pai and coworkers synthesized a binary g-C_3_N_4_/ZnO direct Z-scheme heterojunction nanocomposite using an exfoliation process [152]. The resultant composite showed enhanced electron mobility, high redox potential, and excellent semiconducting properties. The photocatalytic performance of this composite was evaluated by the photodegradation of malachite green (MG) under visible-light irradiation, where it demonstrated outstanding degradation ability, stability, and recyclability. Experimental observations suggested that efficient visible-light harvesting and photogenerated charge separation properties could contribute to the outstanding photocatalytic performance of this heterostructure. The calculated VB and CB band edge positions of g-C_3_N_4_ and ZnO indicated a direct Z-scheme charge-transfer mechanism in the photodegradation process, which was driven by O_2_^•−^ radicals as ROS. Similarly, Arunachalam’s group made efforts to fabricate a binary g-C_3_N_4_/ZnO direct Z-scheme heterojunction as a visible-light-driven photocatalyst for the photodegradation of organic pollutants [153]. Some other heterojunctions were synthesized to improve the photocatalytic efficiency of g-C_3_N_4_ by coupling it with ZnO via sol–gel or hydrothermal methods [154,155,156].

As a method for designing and synthesizing new forms of g-C_3_N_4_-based heterojunction photocatalysts having even better performance, Brasileiro et al. performed a comparative study by synthesizing two different heterojunctions of g-C_3_N_4_ with ZnO and ZnFe_2_O_4_ (ZnO/g-C_3_N_4_ (Zn/gCN) and ZnFe_2_O_4_ (ZF/gCN)) in different mass proportions [157]. A comparison of the photocatalytic performance of these two heterojunctions in the degradation of cefazolin (CFZ) and reactive black 5 (RB5) proved that the Zn/gCN heterojunction with a 50% mass ratio (50-Zn/gCN) could have a higher performance than that of ZF/gCN heterojunctions (Figure 10e,f).

It is believed that the higher performance of 50-Zn/gCN is directly related to the synergistic effect of ZnO and g-C_3_N_4_ and the existence of a direct Z-scheme charge-transfer pathway (Figure 10g), which promotes the interaction of photogenerated electrons in the CB of ZnO and photogenerated holes in the VB of g-C_3_N_4_. As a result, well-separated photogenerated electrons and holes remained in the CB of g-C_3_N_4_ and the VB of ZnO, respectively, to react with pollutants. On the other hand, the existence of a Z-scheme charge-transfer pathway was not possible in ZF/gCN heterojunctions. In addition, the CB position of ZnFe_2_O_4_ is not sufficiently positive (+2.17 eV) to oxidize water molecules through photogenerated holes since the standard redox potential of (H_2_O/OH^•^) is +2.27 eV. In addition, the reason behind the higher performance of the 50-Zn/gCN heterojunction is related to the greater mass proportion of g-C_3_N_4_, which could increase its visible-light absorption property. However, the slightly lower performance of the 80-Zn/gCN heterojunction might be associated with the increased clustering of ZnO and g-C_3_N_4_ particles.

Within the context of synthesizing superior g-C_3_N_4_/ZnO direct Z-scheme heterojunctions, another effort has recently been put forward by Silva and coworkers. They prepared different g-C_3_N_4_/ZnO heterojunctions to investigate the influence of the mass ratio on the photocatalytic performance [158]. SEM/TEM analyses revealed the formation of heterojunction interfaces in all samples. The photocatalytic activity for degrading Rh B dye in an aqueous solution showed the higher performance of the g-C_3_N_4_/ZnO ≥ 1 heterojunction with the mass ratio g-C_3_N_4_ (1.0) and ZnO (0.5), which is attributed to the increased amount of g-C_3_N_4_, since the presence of carbonaceous materials in a heterojunction can enhance visible-light absorbance and transfer energy to ZnO. However, the greater amount of g-C_3_N_4_ in the heterojunction led to a noticeable decrease in catalytic performance. Additionally, the formation of the direct Z-scheme charge-transfer pathway could bring about the effective separation of photogenerated charge carriers. Through the use of different radical scavengers, O_2_^•−^ radicals were identified as the main ROS in the photodegradation of Rh B dye. In another work recently, Girish et al. generated a direct Z-scheme heterojunction of ZnO and g-C_3_N_4_ using a simple solution-mixing method [159]. Under visible-light irradiation, the ZnO/g-C_3_N_4_ heterojunction hybrid demonstrated excellent degradation of MB dye and cyclic stability. The quenching effects of different scavengers indicated that holes and O_2_^•−^ radicals played crucial roles in the dye degradation. Likewise, other g-C_3_N_4_/ZnO-based heterojunctions were also prepared recently for the treatment of different organic pollutants [160,161,162]. There are a large number of studies related to binary g-C_3_N_4_/ZnO-based direct Z-scheme heterojunction photocatalysts, which cannot be described one by one. So, their photocatalytic activities are summarized in Table 2.

#### 6.2.2. Formation of Ternary g-C_3_N_4_/ZnO-Based Direct Z-Scheme Heterojunction Photocatalysts

**Figure 11:**
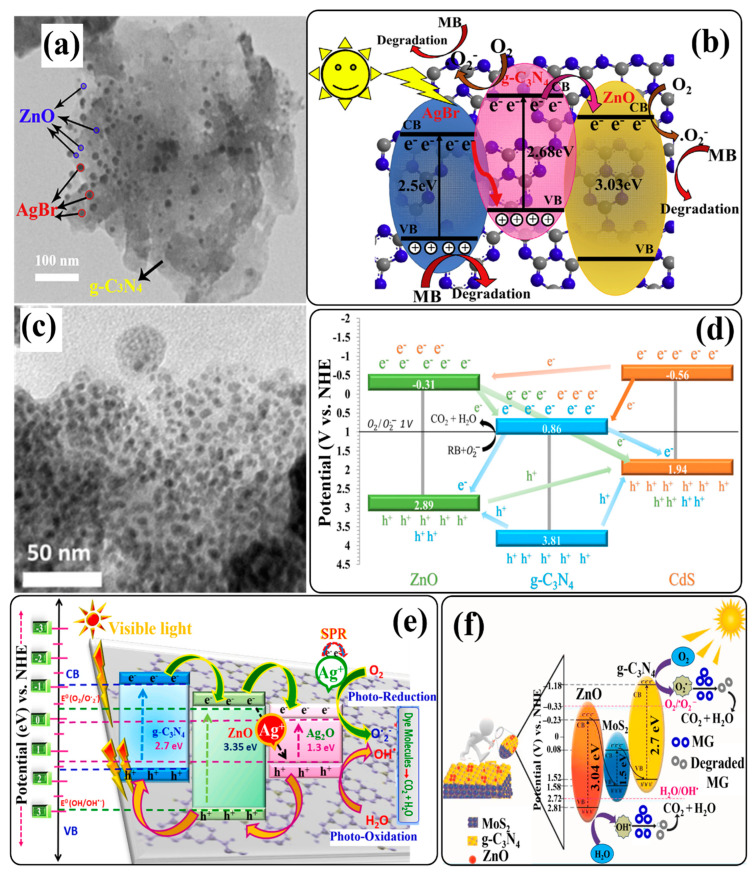
(**a**) TEM image of CN/AB/ZO 30 and (**b**) schematic illustration of the proposed mechanism for the photocatalytic degradation of MB under irradiation by visible light with g-C_3_N_4_/AgBr/ZnO. (**c**) HR-TEM image of the as-fabricated CdS@ZnO/g-C_3_N_4_ nanocomposite and (**d**) the general mechanism of the photocatalytic performance of the ternary composite CdS@ZnO/g-C_3_N_4_. (**e**) Schematic illustration of plausible Z-scheme mechanism exposing energy-band diagram with charge separation/transfer pathway in a g-C_3_N_4_/ZnO-Ag_2_O ternary PCs for photodegradation of organic pollutants under visible-light exposure. (**f**) Charge transfer based on the Z-scheme mechanism in MoS_2_/g-C_3_N_4_/ZnO. Reprinted from Refs. [163,166,167,169] with permission from Elsevier.

Although binary Z-scheme heterojunction composites are regarded as the most popular photocatalysts, the introduction of a new semiconductor material into the binary heterojunction system significantly changes its photocatalytic properties. According to the outcomes from previous research works, ternary heterojunctions have proven to be potential photocatalysts that can provide enhanced visible-light absorption properties, the efficient generation of charge carriers, and improved transformation and separation of photogenerated charge carriers. Therefore, researchers are focusing on the development of new ternary systems from binary systems. Nevertheless, research in this area is still insufficient, and more studies are needed. A ternary Z-scheme heterojunction photocatalyst consists of three types of semiconductor materials. Therefore, the selection of suitable semiconductor materials for the construction of ternary Z-scheme heterojunction photocatalysts is very crucial. As a consequence, Azimi et al. made an effort to design and construct a ternary direct Z-scheme heterojunction photocatalyst via a sonication-assisted deposition technique. They synthesized g-C_3_N_4_/AgBr/ZnO heterostructure nanocomposites by introducing AgBr into g-C_3_N_4_/ZnO heterostructures with different weight ratios of g-C_3_N_4_ relative to AgBr and ZnO in equal amounts, since AgBr is a fascinating semiconductor with a band gap of about 2.5 eV, and its VB/CB locations make it suitable for coupling with g-C_3_N_4_ and ZnO [163]. TEM images showed well-scattered anchoring of AgBr and ZnO nanoparticles on g-C_3_N_4_ in the heterojunction (Figure 11a). MB dye was selected to examine the photocatalytic performance of the as-prepared photocatalyst under visible-light irradiation. Among different samples, the nanocomposite with a 30% weight ratio of g-C_3_N_4_ (g-C_3_N_4_/AgBr/ZnO 30) was able to show higher performance, which was attributed to the effective separation and transformation of photogenerated charge carriers at the interface across the heterojunction of ternary g-C_3_N_4_/AgBr/ZnO 30 nanocomposite.

Also, it is supposed that another factor that could enhance the photodegradation process is the surface charge on the ternary photocatalyst, which was determined using zeta potential measurement. The g-C_3_N_4_/AgBr/ZnO 30 nanocomposite was much more negatively charged (−1037 mV) compared to g-C_3_N_4_ (−532 mV). This negatively charged surface of g-C_3_N_4_/AgBr/ZnO 30 caused an electronic attraction between photocatalyst and cationic MB dye molecules, thereby increasing the photodegradation process. According to the evaluated potentials of CBs and VBs of the three components, a Z-scheme mechanism was proposed for the photodegradation of MB dye using the g-C_3_N_4_/AgBr/ZnO 30 nanocomposite, as shown in Figure 11b. The CB/VB potentials for g-C_3_N_4_, AgBr, and ZnO were estimated at −1.10/+1.58 eV, +0.06/+2.56 eV, and −0.415/+2.615 eV, respectively. Therefore, during light irradiation, the photoexcited electrons in the CB of AgBr join the holes in the VB of g-C_3_N_4_ for recombination, resulting in the effective separation of photogenerated electrons in the CB of g-C_3_N_4_. Then, these electrons reduce O_2_ to O_2_^•−^ radicals. In addition, some of the photogenerated electrons are transferred from the CB of g-C_3_N_4_ to the CB of ZnO, which also reduces O_2_ to O_2_^•−^ radicals because of its negative potential (−0.33 eV). By contrast, the photogenerated holes in the VB of g-C_3_N_4_ oxidize MB dye directly rather than reacting with H_2_O to generate OH^•^ radicals. The identification of active ROS involved in the photodegradation process of MB dye using different scavengers suggested that holes and O_2_^•−^ radicals played major roles, while OH^•^ radicals played no significant role in the degradation process.

An environmentally friendly strategy was reported by a group (Thang et al.) to synthesize a Ag/g-C_3_N_4_/ZnO nanorod composite for the degradation of commercial drugs in the presence of visible light [164]. XRD patterns of the Ag/g-C_3_N_4_/ZnO nanorod composite showed that the positions and shapes of the peaks remained almost the same as those of the individual g-C_3_N_4_ and ZnO. These results indicated that no secondary phase was formed in the composite, and the Ag nanoparticles were just deposited on g-C_3_N_4_ nanosheets rather than doped. Compared to g-C_3_N_4_, ZnO, and g-C_3_N_4_/ZnO, the Ag/g-C_3_N_4_/ZnO nanorod composite showed substantially enhanced photodegradation activity and excellent reusability for up to five consecutive cycles. The enhanced performance of the Ag/g-C_3_N_4_/ZnO nanorod composite is explained in terms of Z-scheme heterojunction formation and the LSPR effect of Ag nanoparticles. The heterojunction formed between g-C_3_N_4_ and ZnO facilitated the charge separation/migration following the Z-scheme charge-transfer mode in the Ag/g-C_3_N_4_/ZnO nanorod composite. Additionally, the localized surface-plasmon resonance (LSPR) effect of Ag nanoparticles could increase the electron density on the surface of Ag nanoparticles and extend the absorption ability of the composite. In addition, Ag nanoparticles functioned as an electron sink, which also promoted the separation of photogenerated electrons/holes.

Due to its narrow band gap and high conductivity, magnetic α-Fe_2_O_3_ is widely used as a visible-light-driven photocatalyst. It can act as an electron-trapping material to prevent the recombination of photogenerated charge carriers. Yang and coworkers utilized the advantages of magnetic α-Fe_2_O_3_ through the fabrication of a ternary heterojunction. They synthesized a ternary g-C_3_N_4_/ZnO@α-Fe_2_O_3_ nanocomposite by direct pyrolysis and the sol–gel method [165]. The photocatalytic activity of the as-prepared nanocomposite was studied by degrading tetrazine dye under visible-light irradiation. Compared to other samples (g-C_3_N_4_, ZnO, Zno@α-Fe_2_O_3_), the ternary g-C_3_N_4_/ZnO@α-Fe_2_O_3_ nanocomposite showed higher photodegradation efficiency, along with excellent cyclic stability. Overall, the lower band gap (2.6 eV) of the ternary nanocomposite helped to achieve enhanced photodegradation activity. A Z-scheme photocatalytic reaction mechanism of tetrazine oxidation using the ternary g-C_3_N_4_/ZnO@α-Fe_2_O_3_ nanocomposite was proposed. When visible light irradiates the nanocomposite, the photogenerated electrons from the CB of ZnO@α-Fe_2_O_3_ can easily transfer to the VB of g-C_3_N_4_ via Z-scheme heterojunction and combine with the photogenerated holes. Since the CB of g-C_3_N_4_ is more negative (−1.1 eV vs. NHE) than those of ZnO (−0.1 eV) and α-Fe_2_O_3_ (+0.3 eV), the photogenerated electrons in the CB of g-C_3_N_4_ react with O_2_ to produce O_2_^•−^ radicals. On the other hand, the VB position of ZnO (+3.1 eV) is more positive than those of α-Fe_2_O_3_ (+2.4 eV) and g-C_3_N_4_ (+1.57 eV). Hence, the photogenerated holes of g-C_3_N_4_/α-Fe_2_O_3_ migrated to the VB of ZnO and reacted with H_2_O to produce OH^•^ radicals. These ROS are responsible for the degradation process. However, ROS-trapping experiments confirmed that O_2_^•−^ radicals played a significant role, while holes and OH^•^ radicals showed smaller effects on photocatalytic degradation. 

A novel ternary composite of 10% CdS quantum dots over 45% ZnO nanoparticles and 45% g-C_3_N_4_ nanosheets was prepared by Hashem et al. using a green and facile one-pot room-temperature ultrasonic route [166]. The final powder was designated as a CdS@ZnO/g-C_3_N_4_ ternary nanocomposite. HRTEM images of the synthesized CdS@ZnO/g-C_3_N_4_ ternary nanocomposite revealed well-dispersed CdS quantum dots and ZnO nanoparticles over the g-C_3_N_4_ nanosheets (Figure 11c). Different light sources (UV and visible light) were used to assess the photocatalytic behavior of the CdS@ZnO/g-C_3_N_4_ ternary nanocomposite, including binary composites (CdS@ZnO and CdS@g-C_3_N_4_) and their individual counterparts (ZnO and g-C_3_N_4_), toward the photodegradation of Rh B dye at pH 6. It was found that the degradation % of Rh B dye was higher under UV-light irradiation when utilizing the CdS@ZnO/g-C_3_N_4_ ternary nanocomposite. This could be attributed to the highly energetic UV photons compared to the moderately energetic visible photons. In addition, the higher photodegradation efficiency of the ternary nanocomposite compared to binary composites and their individual counterparts is attributed to the synergistic effect of the ternary nanocomposite, which exhibited the lowest electron–hole recombination rate. Also, the photodegradation % of Rh B dye was decreased above and below the pH 6 value. At higher pH, Rh B molecules form zwitterions, leading to an increase in aggregation to form Rh B dimers, which impede the adsorption of Rh B molecules on the pores of the photocatalyst surface, as well as a decrease in degradation %. On the contrary, low pH causes repulsion between Rh B cations and the positive photocatalyst surface, leading to a decrease in degradation. Reusability and cyclability tests of the as-synthesized CdS@ZnO/g-C_3_N_4_ ternary nanocomposite revealed no significant reduction in Rh B photodegradation in four consecutive cycles.

The mechanism of the photocatalytic behavior of the CdS@ZnO/g-C_3_N_4_ ternary nanocomposite was suggested on the basis of the calculated VB/CB energies of each component. As calculated, the VB/CB energies against NHE for ZnO, g-C_3_N_4_, and CdS were +2.89/−0.31 eV, +3.81/+0.86 eV, and +1.94/−0.56 eV, respectively. With the exposure of this ternary nanocomposite to photons, the movement of photogenerated charge carriers takes place along many routes, thereby preventing their recombination. The CB of g-C_3_N_4_ is supposed to be the richest CB in electrons due to its lower energy level compared to those of ZnO and CdS, while the VB of CdS is supposed to be the richest VB in holes due to its higher energy level compared to those of ZnO and g-C_3_N_4_. Therefore, g-C_3_N_4_ may remain the central platform for the reductive degradation pathway, and the VB of CdS can act as an oxidative degradation pathway. The individual binary nanocomposite can significantly reduce the electron–hole recombination rate through the creation of type II heterojunctions. Moreover, the effective charge separation in the ternary nanocomposite was maintained through the creation of an additional Z-scheme heterojunction in which electrons transfer from the CBs of two semiconductors to the VB of the third one (from the CBs of ZnO and g-C_3_N_4_ to the VB of CdS). Hence, it is concluded that the enhanced photocatalytic activity of the CdS@ZnO/g-C_3_N_4_ ternary nanocomposite is associated with the synergistic effect of type II/Z-scheme heterojunctions, which greatly reduced the recombination of photogenerated electrons and holes (Figure 11d). Radical-trapping experiments using different scavengers proved that O_2_^•−^ radicals played a major role in the photodegradation process utilizing the CdS@ZnO/g-C_3_N_4_ ternary nanocomposite.

For improving the photocatalytic performance and hindering the recombination of photogenerated electrons and holes, the combination of binary ZnO/g-C_3_N_4_ heterojunctions with narrow-band-gap semiconductors is attracting increased attention. Hence, Vignesh et al. prepared a Ag/Ag_2_O-combined g-C_3_N_4_/ZnO (g-C_3_N_4_/ZnO-Ag_2_O) ternary composite by a calcination and hydrothermal process [167]. Ag_2_O is a p-type semiconductor having a narrow optical band gap (~1.3 eV) and can act as an efficient visible-light photocatalyst. XPS suggested that the deconvoluted doublet spectra of Ag 3d peaks of Ag_2_O located at ~369.9 and 375.3 eV were attributed to Ag 3d_5/2_ and Ag 3d_3/2_ of metallic Ag^+^, which originated in the formation of monovalent Ag/Ag_2_O. The photocatalytic activity of as-prepared g-C_3_N_4_/ZnO-Ag_2_O was evaluated by the photodegradation of MB dye and 4-CP aqueous solutions under visible-light irradiation. Compared to g-C_3_N_4_, P25 TiO_2_, g-C_3_N_4_/ZnO photocatalysts, the g-C_3_N_4_/ZnO-Ag_2_O ternary composite exhibited superior photodegradation ability of MB dye and 4-CP, which might be attributed to the successful blending of Ag/Ag_2_O NPs with the g-C_3_N_4_/ZnO composite, which could form an energetic heterostructure with an efficient interface so that the effective separation/transfer of photogenerated charge carriers was maintained. In addition, Ag NPs in Ag/Ag_2_O helped to trap and effectively separate the photogenerated electrons, thereby decreasing their recombination rate.

From the calculated band energy positions, a Z-scheme photocatalytic mechanism was proposed for the g-C_3_N_4_/ZnO-Ag_2_O ternary heterostructure (Figure 11e). The calculated CB and VB edge potentials of ZnO were −0.38 and +2.29 eV; those of g-C_3_N_4_ were −1.13 and +1.57 eV; those of Ag_2_O were +0.14 and +1.44 eV. From these data, it seems that the CB energy potential of g-C_3_N_4_ (−1.13 eV vs. NHE) is more negative than those of ZnO (−0.38 eV vs. NHE) and Ag_2_O (+0.14 eV vs. NHE). On the other hand, the VB edge potential of ZnO (+2.96 eV vs. NHE) is more positive than those of g-C_3_N_4_ (+1.57 eV vs. NHE) and Ag_2_O (+1.44 eV vs. NHE). Hence, this situation is favorable for the movement of photoexcited holes in the VB and electrons in the CB of g-C_3_N_4_ to ZnO or Ag_2_O during light irradiation. The CB of g-C_3_N_4_ is higher than that of ZnO/Ag_2_O; hence, Ag/Zn ions could function as the charge transmission link, and photoexcited electrons from the CB of Ag_2_O combined with Ag^+^ to form metallic Ag^0^, which could act as the electron sink to prevent electron–hole pair recombination and the photocorrosion of Ag_2_O by g-C_3_N_4_/ZnO. Interestingly, the surface-plasmon resonance (SPR) properties of metallic Ag could also further enhance the electron migration for identical CB edge positions of g-C_3_N_4_ and ZnO surfaces. In this way, photoexcited electrons accumulated in the CB of Ag_2_O were trapped by dissolved O_2_ to yield O_2_^•−^ radicals with an oxidation potential of (O_2_/O_2_^•−^) = 0.14 eV vs. NHE. Similarly, the photoexcited holes in the VB of g-C_3_N_4_ might have migrated to the VB of ZnO/Ag_2_O, leading to the effective separation. In addition, a Z-scheme heterojunction was established between ZnO and Ag_2_O, and photogenerated electrons in the CB of ZnO were directly transferred to the VB of Ag_2_O. Some of the photoexcited holes in the VB of Ag_2_O reacted with H_2_O/OH^−^ to produce OH^•^ radicals. Hence, O_2_^•−^ and OH^•^ radicals (ROS) were accountable for the photodegradation of MB dye and 4-CP. Radical scavenger experiments indicated the major role of these ROS in the order OH^•^ > h^+^ > O_2_^•−^.

Another synthesis approach for a novel Z-scheme ternary hybrid of a g-C_3_N_4_@Ag-ZnO nanocomposite was reported for the degradation of MB dye under visible-light irradiation [168]. MoS_2_, an important member of the chalcogenide family, possesses a layered structure with a large surface area and fascinating optical properties. Therefore, researchers have been using MoS_2_ as a co-catalyst to enhance the photocatalytic performance of binary or ternary heterojunction photocatalysts. The combination of a ZnO/g-C_3_N_4_ composite with a MoS_2_ co-catalyst may increase the lifetime and separation of photogenerated charge carriers. In the recent past, Choi and coworkers reported a strategy for the synthesis of a novel ternary Z-scheme heterojunction of a MoS_2_/g-C_3_N_4_/ZnO nanocomposite using hydrothermal and exfoliation methods [169]. The photocatalytic performance of the ternary MoS_2_/g-C_3_N_4_/ZnO nanocomposite was evaluated by degrading MG dye under visible-light irradiation. Compared to MoS_2_/g-C_3_N_4_ and MoS_2_/ZnO nanocomposites, the ternary nanocomposite showed excellent photocatalytic performance, which is attributed to three facts: (i) the heterojunction formed in the MoS_2_/g-C_3_N_4_/ZnO nanocomposite facilitated the movement and separation of photogenerated electrons and holes; (ii) MoS_2_ in the ternary nanocomposite increased the life span of charge carriers by accelerating their transference in the g-C_3_N_4_/ZnO interface; (iii) the surface charge of the ternary nanocomposite was obtained by zeta-potential measurements. At neutral pH, the surface charge of this composite was more negative (−1.43 mV), which became more favorable for the adsorption of cationic MG dye on the surface of the ternary nanocomposite, thereby facilitating the degradation process. Furthermore, the stability and reusability of the prepared ternary photocatalyst were evaluated by performing five cycles of MG dye degradation. For the first three cycles, the efficiency did not decrease significantly, but after four cycles, the efficiency decreased drastically, which might be associated with the decrease in the adsorption of MG dye molecules.

To elucidate the charge-transfer mechanism in a ternary MoS_2_/g-C_3_N_4_/ZnO nanocomposite, the band gap and band edge positions of each component were determined. The calculated band gaps of MoS_2_, g-C_3_N_4_, and ZnO were +1.5, +2.7, and +3.04 eV, respectively, while the E_CB_/E_VB_ values for MoS_2_, g-C_3_N_4_, and ZnO were calculated to be +0.08/+1.58 eV, −1.18/+1.52 eV, and −0.23/+2.81 eV, respectively. From these band structures, a dual Z-scheme charge-transfer mechanism was proposed for the ternary MoS_2_/g-C_3_N_4_/ZnO nanocomposite (Figure 11f). Under light irradiation, the photogenerated electrons in the CB of ZnO combined with photogenerated holes in the VB of MoS_2_, followed by the combination of photogenerated electrons in the CB of MoS_2_ with photogenerated holes in the VB of g-C_3_N_4_. In this way, MoS_2_ nanosheets in the ternary nanocomposite demonstrated a crucial role in increasing the lifespan of photogenerated charge carriers by enhancing their migration in the ZnO/g-C_3_N_4_ interface. Finally, this led to the effective separation of photogenerated electrons and holes in the CB of g-C_3_N_4_ and the VB of ZnO, respectively. Then, these photogenerated holes reacted with H_2_O to generate OH^•^ radicals, and electrons reacted with O_2_ to generate O_2_^•−^ radicals, which were involved in the photodegradation of MG dye. Scavenger studies showed that OH^•^ and O_2_^•−^ radicals were the active ROS responsible for degradation reactions. 

Likewise, another new multicomponent direct Z-scheme catalyst was designed and fabricated by combining g-C_3_N_4_ with Mg-doped ZnO and a zeolitic imidazolate framework (ZIF-8) via a chemical precipitation method [170]. The photocatalytic activity of the as-prepared multicomponent photocatalyst (Mg-ZnO/g-C_3_N_4_@ZIF-8) was assessed by the photodegradation of illicit drugs in the presence of visible light. NaBH_4_ was also added to the system to enhance the reduction of O_2_ and the generation of O_2_^•−^ radicals. The excellent result obtained with the multicomponent Mg-ZnO/g-C_3_N_4_@ZIF-8 photocatalyst is ascribed to the connection between Mg-ZnO, g-C_3_N_4_, and ZIF-8, exhibiting a close contact interface, and the porous structure of ZIF-8. The close contact interface was beneficial for transferring photogenerated charge carriers, and the porous structure led to a higher adsorption affinity toward organic pollutants. Recently, a ternary composite of a g-C_3_N_4_-ZnO/BiOBr Z-scheme heterojunction photocatalyst was synthesized, and its photocatalytic activity was evaluated by the photodegradation of MO dye under visible-light irradiation [171]. Reports regarding ternary g-C_3_N_4_/ZnO-based direct Z-scheme heterojunction photocatalysts are described in Table 3.

#### 6.2.3. Formation of Metal/Non-Metal-Doped g-C_3_N_4_/ZnO-Based Direct Z-Scheme Heterojunction Photocatalysts

**Figure 12:**
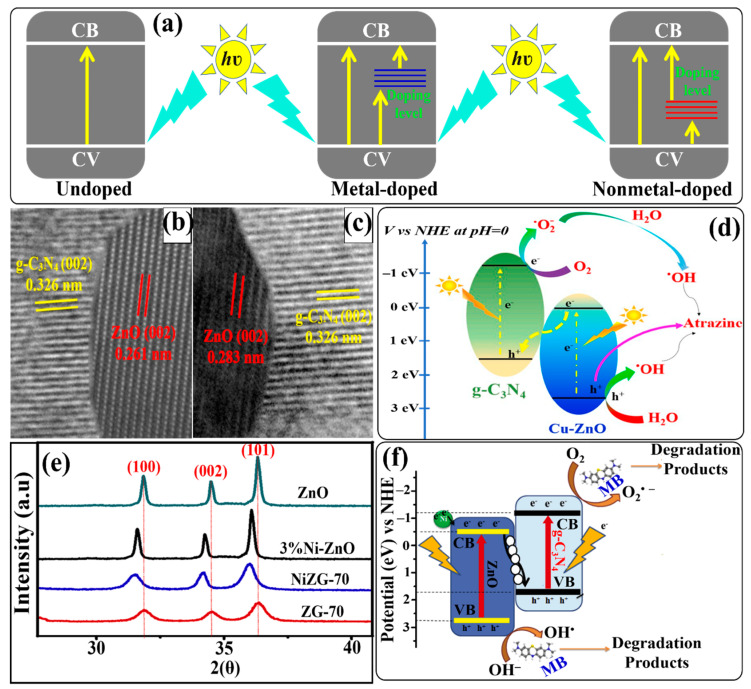
(**a**) Generation of doping level between CB and VB by metal/non-metal doping in a semiconductor photocatalyst, (**b**) HRTEM images of ZnO/g-C_3_N_4_ and (**c**) Cu-ZnO/g-C_3_N_4_. (**d**) Photocatalytic mechanism for degradation of atrazine by the synthesized Cu-ZnO/g-C_3_N_4_. (**e**) X-ray diffraction patterns of ZnO, Ni/ZnO, NiZG-70, and ZG-70 composites and (**f**) the proposed mechanism for the photocatalytic dye degradation activity of NiZG-70 nanocomposites. Reprinted from Refs. [174,177] with permission from Elsevier.

Doping a semiconductor photocatalyst with a metal or non-metal is a widely accepted strategy for enhancing the photocatalytic performance of that photocatalyst. Many reports have demonstrated that the doping process can generate a doping level between the CB and VB (Figure 12a). As a result, the photoenergy required to excite the electrons decreases; the band gap of the semiconductor decreases; the visible-light absorption ability increases; and the interfacial charge transfer and separation ability of the photocatalyst increases due to the electronic synergistic effect between the semiconductor and dopant [15,172]. The doping of g-C_3_N_4_ with non-metals (like C, S, P, O, and F) has been employed to improve its photocatalytic efficiency, since non-metal doping increases visible-light absorption and charge separation ability. Also, non-metal doping causes the tuning of the g-C_3_N_4_ structure by replacing either C or N atoms, which then affects both the VB and CB. The doping of g-C_3_N_4_ with metals (noble metals, rare-earth metals, or alkali metals) leads to band-gap narrowing and surface area improvement. Moreover, inserting metals into the g-C_3_N_4_ framework consequently enhances its light absorption ability [8]. Non-metal or metal doping brings about significant changes in the crystal lattice and optical properties of ZnO. For example, it generates impurity levels, narrows the band gap, and increases the sensitivity to visible-light absorption. In addition, metal atoms doped into the ZnO photocatalyst can act as charge traps to facilitate the charge-transfer process and to prevent the recombination of electron–hole pairs. However, each dopant has a specific effect on the optical and photocatalytic properties of ZnO [173].

##### Formation of Metal-Doped g-C_3_N_4_/ZnO-Based Direct Z-Scheme Heterojunction Photocatalysts

In view of the foregoing research, and considering the advantages of metal doping into the ZnO crystal lattice, Truc et al. proposed a synthesis strategy of a direct Z-scheme photocatalyst coupling g-C_3_N_4_ with Cu-doped ZnO. Utilizing autoclave heating and a calcination process, they successfully incorporated Cu into the ZnO crystal lattice and integrated it with g-C_3_N_4_ to create a Cu-ZnO/g-C_3_N_4_ direct Z-scheme heterojunction photocatalyst [174]. The incorporation of Cu into ZnO could reduce the ZnO band gap and extend its visible-light absorption ability. HRTEM images showed that ZnO and Cu-ZnO nanoparticles were deposited on a g-C_3_N_4_ nanolayer. The lattice distances of g-C_3_N_4_ (002) in doped and undoped samples were similar. However, the lattice distance of ZnO (002) in Cu-ZnO was wider (0.283 nm) than that of undoped ZnO (0.261 nm). This increase in lattice distance verified the partial substitution of Cu in the Zn site in the ZnO matrix (Figure 12b,c). Different samples were employed to evaluate the photodegradation activity toward atrazine under visible-light irradiation. Compared to g-C_3_N_4_ and ZnO/g-C_3_N_4_, the Cu-ZnO/g-C_3_N_4_ photocatalyst showed much better performance and great stability during a long-time degradation process. As mentioned above, the reason behind this performance is the visible-light absorption ability of Cu-ZnO/g-C_3_N_4_ to generate/separate electron–hole pairs. The visible-light absorption ability of Cu-ZnO/g-C_3_N_4_ was ascribed to the role of the Cu dopant, which decreased the band gap of Cu-ZnO to 2.87 eV and produced an intermediate band to aid electron transfer from the VB to the CB of ZnO.

From the determined CB/VB positions of g-C_3_N_4_ and Cu-ZnO, a direct Z-scheme charge-transfer mechanism in the Cu-ZnO/g-C_3_N_4_ photocatalyst was proposed (Figure 12d). The calculated CB and VB positions of g-C_3_N_4_ were −1.17 and +1.49 V (vs. NHE), respectively. Similarly, for Cu-ZnO, the CB and VB positions were +0.21 and +3.08 V (vs. NHE), respectively. Upon light irradiation, the excited electrons in the CB of Cu-ZnO combined with the holes in the VB of g-C_3_N_4_, preserving the electrons and holes in the CB of g-C_3_N_4_ and the VB of Cu-ZnO, respectively. In this way, the Cu-ZnO/g-C_3_N_4_ photocatalyst not only prevented the recombination of electron–hole pairs but also improved their redox potential. Finally, the accumulated electrons in the CB of g-C_3_N_4_ reacted with dissolved oxygen to generate O_2_^•−^ radicals, which further reacted with water to generate OH^•^ radicals for the degradation of atrazine, rather than its direct degradation by O_2_^•−^ radicals. On the other hand, the accumulated holes in the VB of Cu-ZnO reacted with water to generate OH^•^ radicals for the indirect degradation of atrazine; however, holes were also involved in the degradation process directly. The direct/indirect degradation ratios were equal. Another direct Z-scheme heterojunction of metal-doped ZnO coupled with g-C_3_N_4_ was reported by Humayun and coworkers [175]. They synthesized spherical flowers of Sr-doped ZnO and g-C_3_N_4_ by a one-pot facile method. The prepared Sr-ZnO/g-C_3_N_4_ heterojunction showed enhanced degradation activity toward methylene green (MG) dye under UV-vis irradiation. Likewise, a nanocomposite of g-C_3_N_4_/Cd-doped ZnO was synthesized for the degradation of MB dye in visible light [176].

Ni is known as a low-cost transition metal with tremendous electron conduction properties and can serve as a dynamic element in designing visible-light-responsive photocatalysts. With this belief, a novel photocatalyst of Ni-doped ZnO nanoparticles coupled with g-C_3_N_4_ was reported [177]. It was claimed that this type of composite was reported for the first time. Using a chemical co-precipitation method, a series of Ni/ZnO/g-C_3_N_4_ (NiZG) composites were fabricated. In this process, first, a series of Ni/ZnO nanoparticles were synthesized with various wt%. Among these, 3% Ni/ZnO nanoparticles exhibited maximum MB dye degradation ability. So, 3% Ni/ZnO nanoparticles were selected for the fabrication of composites and modified by coupling with different wt% (30, 40, 50, 60, 70, 80, and 85) of g-C_3_N_4_. Finally, these samples were abbreviated NiZG-30, NiZG-40, NiZG-50, NiZG-60, NiZG-70, NiZG-80, and NiZG-85. XRD analysis showed that there was a small shift in the major diffraction peaks of 3% Ni/ZnO and NiZG-70 toward the lower (2θ) angle. Such shifting validated the doping of Ni into ZnO. Also, Ni doping did not enhance or diminish the diffraction peaks of 3% Ni/ZnO and NiZG-70, which further confirmed that the wurtzite structure of ZnO was not altered (Figure 12e). The photocatalytic degradation of MB dye utilizing as-fabricated composites under sunlight irradiation showed that the NiZG-70 composite could degrade 100% of the dye in 70 min. Importantly, there was no noticeable decrease in the degradation performance of the NiZG-70 composite even after six consecutive runs. This outcome was favored by the low band gap (2.25 eV), improved charge separation, and visible-light absorption ability of the NiZG-70 composite. In contrast, the photocatalytic performance of NiZG-80 and NiZG-85 composites was low due to the higher weight ratio of g-C_3_N_4_, which served as a recombination spot for photogenerated electron–hole pairs.

Following the previously reported band positions of ZnO and g-C_3_N_4_, a reasonable mechanism regarding MB dye degradation over the NiZG-70 composite was proposed, as shown in Figure 12f. According to the presented schematic of the charge-transfer mode, it seems that a direct Z-scheme charge-transfer mechanism occurs in the NiZG-70 composite. Under solar irradiation, the photogenerated electrons in the CB of ZnO combined with the photogenerated holes in the VB of g-C_3_N_4_, resulting in the effective separation of photogenerated electrons and holes in the CB of g-C_3_N_4_ and the VB of ZnO. Additionally, the doped Ni atoms in the interface served as a mediator to transfer electrons from ZnO to g-C_3_N_4_ and controlled the recombination of photogenerated electron–hole pairs. Afterward, the photogenerated electrons in the CB of g-C_3_N_4_ reacted with oxygen to generate O_2_^•−^ radicals, and photogenerated holes in the VB of ZnO reacted with water to generate OH^•^ radicals. Then, these ROS were involved in the degradation process. Different ROS-trapping experiments indicated that O_2_^•−^ and OH^•^ radicals were the primary ROS for the MB dye degradation process, rather than its direct degradation by holes.

Another Cu-doped ZnO/g-C_3_N_4_ composite was synthesized by Shen et al. [178]. The Cu-ZnO/g-C_3_N_4_ composite synthesized by the hydrothermal method followed by calcination exhibited enhanced degradation of ciprofloxacin under visible-light irradiation. As verified by different characterization techniques, the enhanced degradation performance of the Cu-ZnO/g-C_3_N_4_ composite was attributed to the improved visible-light absorption ability and photogenerated electron–hole separation property due to a Z-scheme transfer route established at the heterojunction interface. Free-radical-trapping experiments signified the increased formation of OH^•^ radicals from photoinduced holes in the VB of ZnO and photoinduced electrons in the CB of g-C_3_N_4_ via the formation of O_2_^•−^ radicals. In order to further broaden the visible-light response of ZnO and extend the lifetime of photogenerated charge carriers, Z-scheme heterojunctions of g-C_3_N_4_ coupled with ZnO doped with one or more metals have been synthesized in recent years [179,180,181].

##### Formation of Non-Metal-Doped g-C_3_N_4_/ZnO-Based Direct Z-Scheme Heterojunction Photocatalysts

**Figure 13:**
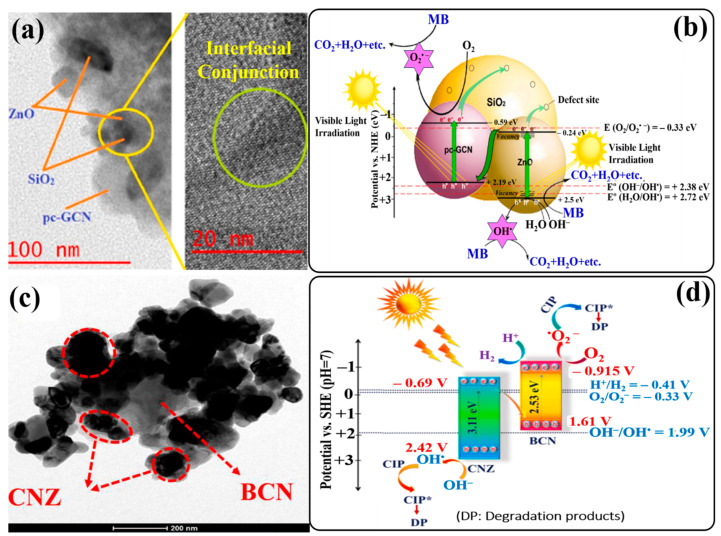
(**a**) A low-scale HR-TEM image of pc-GCN/15-SiO_2_/5-ZnO, (**b**) schematic diagram of the proposed photocatalytic mechanism of MB degradation by the pcGCN/15-SiO_2_/5-ZnO nanocomposite under WLLI. (**c**) TEM image of CNZ/BCN (1:1) and (**d**) schematic illustration of the proposed mechanism for CIP degradation by CNZ/BCN (1:1): Z-scheme-mediated pathway. Reprinted from Refs. [185,190] with permission from Elsevier.

Considering the benefits of non-metal doping into g-C_3_N_4_ and ZnO, Mohamed and coworkers synthesized a novel hierarchical heterojunction consisting of in situ C-doped g-C_3_N_4_ wrapped around C, N-codoped ZnO using a bio-templated hydrothermal method [182]. The resultant C-g-C_3_N_4_@C and N-ZnO core–shell heterojunction exhibited excellent performance in the photodegradation of bisphenol A (BPA) under simulated solar irradiation. This performance was associated with the adequate separation of photogenerated electron–hole pairs through a direct Z-scheme charge-transfer mode from C, N-codoped ZnO to in situ C-doped g-C_3_N_4_. Moreover, in situ C doping in g-C_3_N_4_ and C, N codoping in the ZnO lattice significantly reduced the band gaps of both photocatalysts. Some research works have focused on the synthesis of Z-scheme heterojunctions with higher charge separation ability by coupling ZnO with S- and F-doped g-C_3_N_4_ for the removal of organic pollutants [183,184]. To study the ideal features of amorphous nanoparticles in an amorphous/crystalline heterogeneous nanocomposite on electronic conjunction, charge-transfer performance, and photocatalytic activity, Sareshkeh et al. synthesized a heterogeneous nanocomposite of amorphous ZnO and SiO_2_ and P, C-co-modified crystalline g-C_3_N_4_ by using a calcination process [185]. The prepared nanocomposite was composed of 15 wt% SiO_2_, 5 wt% ZnO, and P, C-codoped g-C_3_N_4_ (pc-GCN/15-SiO_2_/5-ZnO). HRTEM images revealed that coupled ZnO and SiO_2_ nanoparticles were uniformly dispersed in the pc-GCN nanosheets, creating suitable physiochemical interactions. These interactions resulted in strong interfacial conjunction and improved charge transport within the photocatalyst (Figure 13a).

The photocatalytic performance of a pc-GCN/15-SiO_2_/5-ZnO nanocomposite was evaluated by degrading MB dye under visible-light irradiation from 200 W white-LED-light irradiation (WLLI). Different samples, like GCN, pc-GCN, pc-GCN/5-ZnO, pc-GCN/10-ZnO, pc-GCN/10-SiO_2_, pc-GCN/15-SiO_2_, and pc-GCN/15-SiO_2_/5-ZnO, were utilized for comparison in the degradation experiments. Among these, the pc-GCN/15-SiO_2_/5-ZnO heterogeneous nanocomposite exhibited excellent photocatalytic performance, even after the fifth run. Many factors were associated with the robust performance of pc-GCN/15-SiO_2_/5-ZnO. Specifically, the main factors were effective interfacial electronic conjunction; higher light-harvesting ability; a reduced band gap; and suppressed charge carrier recombination. Additionally, the amorphous phases of ZnO and SiO_2_ could create mid-gap states in the VB edge, decrease the band gap, and improve the photocatalytic performance of the pc-GCN/15-SiO_2_/5-ZnO nanocomposite under visible-light irradiation. The amorphous nature of SiO_2_ could also play an important role in electron trapping through defect sites, thereby preventing the recombination of photogenerated electron–hole pairs. In addition, the hydrophilic surface of the pc-GCN/15-SiO_2_/5-ZnO photocatalyst due to the presence of hydroxyl and amine functional groups had a beneficial role in the photocatalytic performance. The higher wettability of the hydrophilic pc-GCN/15-SiO_2_/5-ZnO photocatalyst could reduce the mass transfer on the photocatalyst surface and improve the contact between the photocatalyst’s surface and MB dye solution.

On the basis of the previously reported band gaps of ZnO, SiO_2_, and pc-GCN and the VB/CB potentials of ZnO and pc-GCN, a Z-scheme charge-transfer mechanism was proposed for a pc-GCN/15-SiO_2_/5-ZnO photocatalyst (Figure 13b). When it was exposed to WLLI, photogenerated electrons were moved to their CB, leaving photogenerated holes in their respective VB, while the higher band gap of SiO_2_ (9.0 eV) played a significant role in trapping the photogenerated electrons in the CB of ZnO and pc-GCN through the defect sites of SiO_2_. Therefore, the recombination of photogenerated charge carriers was significantly inhibited. Then, the photogenerated electrons in the CB of ZnO were transferred to the VB of pc-GCN to combine with the photogenerated holes, as these electrons were not able to generate O_2_^•−^ radicals from oxygen since the E_CB_ of ZnO (−0.24 eV) was more positive than E^º^ (O_2_/O_2_^•−^) = −0.33 eV. Meanwhile, the photogenerated electrons in the CB of pc-GCN could reduce oxygen to O_2_^•−^ radicals since the E_CB_ of pc-GCN (−0.59 eV) was more negative than E^º^ (O_2_/O_2_^•−^). On the other hand, the photogenerated holes in the VB of ZnO could oxidize water molecules or OH^−^ ions to generate OH^•^ radicals since the E_VB_ of ZnO (+2.8 eV) is less negative than E^º^ (H_2_O/OH^•^) = +2.72 eV and E^º^ (OH^−^/OH^•^) = +2.38 eV. The thus-generated ROS were involved in the degradation of MB dye; however, radical-trapping experiments determined that OH^•^ radicals had a more determining role in the degradation process than holes and O_2_^•−^ radicals.

Considering the ideal features of non-metal-doped ZnO in photocatalytic activity, many efforts have been made to design visible-light-active non-metal-doped ZnO/g-C_3_N_4_-based Z-scheme heterojunction photocatalysts. N is thought to be the most suitable p-type dopant for ZnO due to its atomic size and electronic structure. The atomic size and electronegativity of the N atom are very close to those of the O atom. Therefore, a small amount of energy is required to substitute the O atom in the ZnO lattice with the N atom. On that note, strategies for synthesizing visible-light-active Z-scheme heterojunction photocatalysts of N-doped ZnO coupled with g-C_3_N_4_ possessing different morphological structures have been continuously pursued [186,187,188]. Similarly, in the sequence of non-metal doping into ZnO, Hamdy’s group prepared a C-doped ZnO@g-C_3_N_4_ composite by thermally treating commercial ZnO nanoparticles with melamine [189]. The prepared C-doped ZnO@g-C_3_N_4_ composite was applied for the decolorization of methyl green dye under visible light. Compared to g-C_3_N_4_, ZnO, and C-doped ZnO samples, the C-doped ZnO@g-C_3_N_4_ composite showed superior photocatalytic activity with remarkable stability, which was ascribed to the enhanced absorbance of photons by C-doped ZnO and g-C_3_N_4_ in the visible range, the Z-scheme charge-transfer pathway, and the effective electron–hole separation ability of the C-doped ZnO@g-C_3_N_4_ composite. Very recently, a novel work related to the fabrication of a g-C_3_N_4_/ZnO-based heterojunction photocatalyst was reported in which both components, g-C_3_N_4_ and ZnO, were doped with non-metals. Parida’s group fabricated a robust nanocomposite of ZIF-8-derived C, N-codoped ZnO-modified B-doped g-C_3_N_4_ by using an in situ calcination process [190]. It is believed that this was the first report of this type of research work.

Following a similar protocol, a series of three composites were fabricated and labeled according to the precursor weight ratios of C, N-codoped ZnO (CNZ) and B-doped g-C_3_N_4_ (BCN): CNZ/BCN (1:1), CNZ/BCN (1:2), and CNZ/BCN (2:1). Powder XRD (PXRD) patterns of the CNZ/BCN nanocomposite revealed that the distinct and intense diffraction peaks of CNZ were in agreement with JCPDS file no. 00-036-1415, verifying the formation of a hexagonal wurtzite structure. On the other hand, after B-doping, the diffraction peaks of BCN were similar to the characteristic peaks of pristine g-C_3_N_4_. However, the decrease in the intensities of the (100) and (002) planes of BCN as compared to those of g-C_3_N_4_ indicated the substitution of some C atoms by B atoms. Additionally, the (002) plane of BCN was observed to be slightly shifted toward a lower angle, which also indicated the presence of substituted B atoms in the planer ring structure of g-C_3_N_4_. HRTEM characterization was conducted to study the morphological structure of the CNZ/BCN nanocomposite, which showed that CNZ nanoparticles were deposited on the BCN surface (Figure 13c). In order to emphasize the potential application of prepared nanocomposites, including CNZ and BCN, the photocatalytic performance of all samples was assessed for the photodegradation of ciprofloxacin (CIP) under simulated solar-light irradiation. After light irradiation for 60 min, the CNZ/BCN (1:1) nanocomposite showed superior performance for the photodegradation of CIP, which was ascribed to the enhanced photon absorption ability, the fast movement of photogenerated charge carriers through the Z-scheme heterojunction, and the suppressed recombination rate of electron–hole pairs.

With respect to the calculated CB and VB positions of CNZ and BCN, a viable mechanism for the depiction of the reaction route was proposed for the degradation of CIP. The CB/VB positions of CNZ and BCN were calculated to be −0.69 V/+2.42 V and −0.915 V/+1.615 V, respectively. Hence, a Z-scheme charge-transfer pathway was speculated in the CNZ/BCN (1:1) system (Figure 13d). Upon light irradiation, recombination took place between the photogenerated electrons and holes in the CB of CNZ and the VB of BCN, respectively. This resulted in an effective separation of photogenerated electrons and holes in the CB of BCN and the VB of CNZ, respectively. Afterward, the photogenerated electrons in the CB of BCN reacted with dissolved oxygen to generate O_2_^•−^ radicals since the CB potential of BCN is sufficient for the reduction of oxygen molecules (O_2_/O_2_^•−^ = −0.33 V vs. SHE), while the photogenerated holes in the VB of CNZ reacted with water to generate OH^•^ radicals since the VB potential of CNZ is sufficient to oxidize water (OH^•^/OH^−^ = +1.99 V vs. SHE). Additionally, reusability studies of the CNZ/BCN (1:1) nanocomposite showed no substantial variation even after four cycling tests, indicating its high photostability for practical applications.

A comparative analysis of metal and non-metal-doped g-C_3_N_4_/ZnO-based direct Z-scheme heterojunction photocatalysts is summarized in Table 4.

## 7. Formation of g-C_3_N_4_/ZnO-Based Double Z-Scheme Heterojunction Photocatalysts

**Figure 14:**
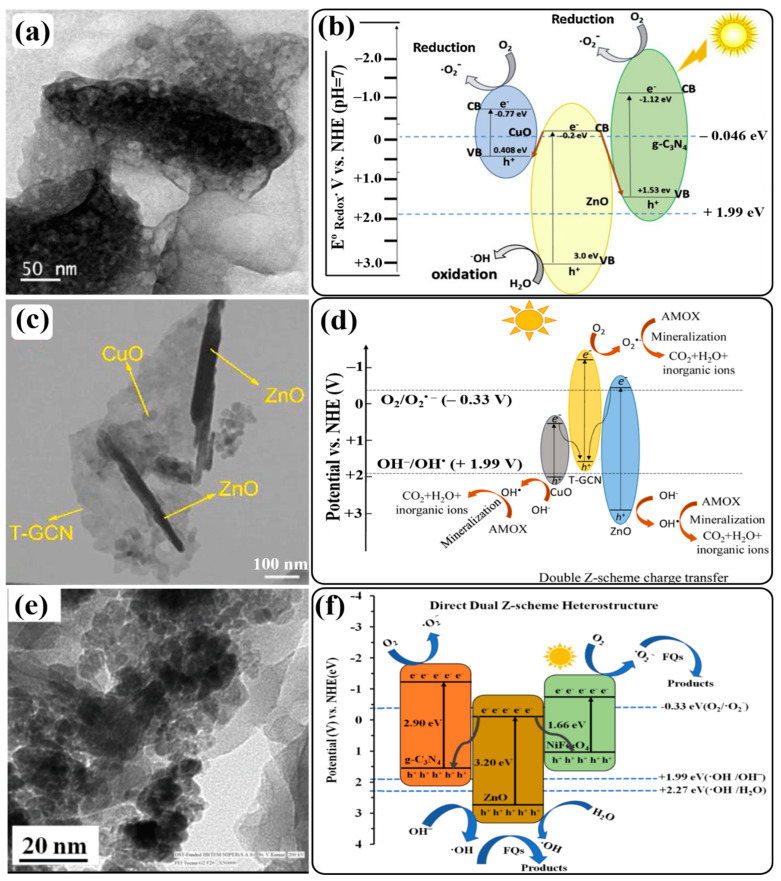
TEM image of the sample CZg (CuO/ZnO/g-C_3_N_4_) heterostructure (**a**) and schematic diagram of the charge migration pathway in CuO/ZnO/g-C_3_N_4_ heterostructure based on Z-scheme heterojunction approach (**b**). FESEM image of CZ@T-GCN (**c**) and schematic illustration for plausible charge relocation and photocatalytic mechanism of AMOX degradation over CZ@T-GCN (**d**). HRTEM image of CZN1 heterostructure (**e**), and schematic illustration of charge-transfer mechanism occurring through direct dual Z-scheme pathway in magnetic CN/ZnO/NiFe_2_O_4_ system under visible-light irradiation (**f**). Reprinted from Refs. [191,194,195] with permission from Elsevier.

To further improve the light absorption ability and charge separation capacity of g-C_3_N_4_/ZnO-based Z-scheme heterojunction photocatalysts, many researchers have assimilated new types of heterojunctions with double Z-scheme charge-transfer channels by coupling a g-C_3_N_4_/ZnO heterojunction with another suitable semiconductor photocatalyst. Hence, the formation of a g-C_3_N_4_/ZnO-based double Z-scheme heterojunction photocatalyst results in the tailoring of a suitable band gap, thereby enhancing the lifetime of photogenerated charge carriers compared to a single Z-scheme g-C_3_N_4_/ZnO photocatalytic system. Bajiri et al. prepared a novel ZnO/CuO/g-C_3_N_4_ (CZg) heterostructure using a solution combustion route [191]. TEM images of the CZg heterostructure showed single- and multilayered nanosheets decorated with very small nanoparticles of CuO and ZnO (Figure 14a). The visible-light-assisted photocatalytic activity of the heterostructure was evaluated by degrading an MB dye solution. Compared to CuO/ZnO heterostructures (with different CuO concentrations on ZnO), the CZg heterostructure demonstrated increased photocatalytic performance at pH 10. It is suggested that the higher performance of the CZg heterostructure is attributed to its light absorption properties and photogenerated charge separation ability. Additionally, at higher pH values, the zeta potential charge (ZPC) of the photocatalyst was estimated to be negative. Hence, at pH 10, adsorption between MB dye molecules on the negatively charged surface of CZg was increased due to the electrostatic force of attraction, which led to the high degradation of MB dye.

Based on the CB and VB band maxima, a double Z-scheme charge-transfer mechanism has been proposed (Figure 14b). The reported CB maxima (CBM) of ZnO, CuO, and g-C_3_N_4_ are −0.2, −0.77, and −1.12 V, respectively. Similarly, the VB maxima (VBM) are +3.0, +0.4, and +1.53 V for ZnO, CuO, and g-C_3_N_4_, respectively. Under visible-light irradiation, electrons are excited to the CB of each component of the CZg heterostructure, leaving behind holes in their respective VB. Assuming the Z-scheme heterojunction, the photogenerated electrons in the CB of ZnO combined with the photogenerated holes in the VBs of CuO and g-C_3_N_4_. Hence, the effective separation and accumulation of electrons in the CBs of CuO and g-C_3_N_4_ were maintained within the heterostructure. As a result, these photogenerated electrons reacted with dissolved O_2_ since the redox potential of CuO and g-C_3_N_4_ (−0.77 and −1.12 V) is enough to produce O_2_^•−^ radicals. On the other hand, holes in the VB of ZnO also have enough potential to produce OH^•^ radicals from water. The thus-produced ROS are involved in degrading MB dye. Scavenger-trapping studies suggested that OH^•^ radicals played an important role in the photocatalytic degradation process. The next attempt regarding a g-C_3_N_4_/ZnO-based double Z-scheme heterojunction was reported by Wang et al. [192]. Utilizing a simple UV-light irradiation method, they designed a direct double Z-scheme oxygen-doped g-C_3_N_4_/Zn_2_SnO_4_/ZnO ternary heterojunction according to the band-bending theory. The as-prepared O-g-C_3_N_4_/Zn_2_SnO_4_/ZnO ternary heterojunction exhibited enhanced photodegradation performance toward Rh B dye under visible-light irradiation. The enhanced performance is supposed to be due to the synergistic effects of the double Z-scheme interfacial charge-transfer system, built-in electric field, and impurity levels that could expand the light absorption and redox abilities of photoinduced charge carriers.

In an attempt to achieve better photocatalytic performance with a direct Z-scheme heterojunction, Khosravi-Nikou and coworkers prepared a CuO-ZnO@g-C_3_N_4_ (Cu/Zn/g) triplex heterojunction nanocomposite through an ultrasound-assisted hydrothermal method [193]. Ultrasound-assisted desulfurization of dibenzothiophene was conducted in the presence of visible light utilizing the as-prepared Cu/Zn/g triplex nanocomposite with a small amount of H_2_O_2_. Experimental results revealed the better performance and stability of the triplex nanocomposite in comparison to the Cu/Zn composite. The possible reason behind this result is explained on the basis of the increased surface area and double Z-scheme charge-transfer mechanism, which could improve the charge separation ability and redox capability of the Cu/Zn/g triplex nanocomposite. A novel double Z-scheme composite as a nano-photocatalyst was fabricated by co-anchoring CuO nanoparticles and ZnO nanorods on thermally exfoliated g-C_3_N_4_ nanosheets (denoted by CZ@T-GCN) via an isoelectric-point-mediated annealing process [194]. TEM image of the CZ@T-GCN ternary nanocomposite displayed a well-dispersed dispersion of CuO nanoparticles and ZnO nanorods on large T-GCN nanosheets with high porosity. Some interactions between CuO/ZnO and T-GCN were also observed, which has great importance for enhancing the charge-transfer process (Figure 14c). The photocatalytic activity of the as-prepared nano-photocatalyst was evaluated by degrading amoxicillin (AMOX) under simulated sunlight. From the findings, the CZ@T-GCN ternary nanocomposite was confirmed to have substantially improved degradation efficiency, while ZnO, CuO, T-GCN, CuO@T-GCN, and CZ resulted in less degradation of AMOX. The improved photodegradation efficiency of the ternary nanocomposite was attributed to the faster transfer and effective separation of photogenerated charge carriers via double Z-scheme channels due to the formation of heterojunctions within the nanocomposite.

The stability and reusability of the CZ@T-GCN ternary nanocomposite were examined by observing five consecutive cycle tests. Compared to the first cycle, only an 8% reduction in the AMOX degradation rate was observed after the fifth cycle, suggesting outstanding stability. However, the slight reduction of 8% might be related to the loss of the photocatalyst during the recovery process and the clogging of pores by AMOX molecules. Based on the positions of the CB minima (CBM) and VB maxima (VBM) of semiconductors, a double Z-scheme charge-transfer mechanism is proposed for the CZ@T-GCN nanocomposite, as shown in Figure 14d. The calculated CBM/VBM for ZnO, T-GCN, and CuO are −0.37/+2.95 V vs. NHE, −1.18/+1.64 V vs. NHE, and +0.59/+2.03 V vs. HHE, respectively. Under simulated solar-light irradiation, electron–hole pairs are produced in each semiconductor. Then, electrons in the CB of CuO and ZnO (electrons with less negative potential) combine with the holes in the VB of T-GCN (holes with less oxidizing potential). Hence, the VBs of CuO and ZnO and the CB of T-GCN actively participated in the generation of ROS due to their approx. redox energy levels. As a result, the photogenerated holes accumulated in the VBs of ZnO and CuO could oxidize OH^−^ ions to produce OH^•^ radicals due to their more positive band potential compared to OH^−^/OH^•^ (+1.99 V vs. NHE). Also, the photogenerated electrons accumulated in the CB of T-GCN could trap O_2_ to produce O_2_^•−^ radicals owing to the more negative CB potential of T-GCN compared to O_2_/O_2_^•−^ (−0.33 V vs. NHE). The as-produced ROS played an effective role in the photodegradation of AMOX according to the following reactions.

(12) $$CZ@T-GCN+h\nu \overset{}{\to }CZ@T-GCN\;(h^{+}+e^{-})$$

(13) $$CZ@T-GCN\;\left(h^{+}+e^{-}\right)\overset{Z-scheme}{\to }T-GCN\;\left(e^{-}\right)+CuO\;\left(h_{vB}^{+}\right)+ZnO\;\left(h_{vB}^{+}\right)\;\;$$

(14) $$T-GCN\;\left(e_{CB}^{-}\right)+O_{2}\;\overset{}{\to }T-GCN+O_{2}^{•-}$$

(15) $$O_{2}^{•-}+H_{2}O\overset{}{\to }H_{2}O_{2}+2{OH}^{-}+O_{2}$$

(16) $$H_{2}O_{2}+{2O}_{2}^{•-}\overset{}{\to }{OH}^{•}+{OH}^{-}+O_{2}$$

(17) $$CuO\;\left(h_{vB}^{+}\right)+{OH}^{-}\overset{}{\to }CuO+{OH}^{•}$$

(18) $$ZnO\;\left(h_{vB}^{+}\right)+{OH}^{-}\;or\;H_{2}O\;\overset{}{\to }ZnO+{OH}^{•}$$

(19) $$Active\;oxidizing\;radicals+AMOX\overset{}{\to }Intermediate\overset{}{\to }\;H_{2}O+{CO}_{2}$$

In the recent past, an innovative double Z-scheme heterojunction photocatalyst was introduced. Singhal and coworkers prepared ternary heterostructures of g-C_3_N_4_/ZnO/NiFe_2_O_4_ (CZN) via a simple sonication–calcination process [195]. Different samples were prepared with different weight ratios of the g-C_3_N_4_/ZnO composite and NiFe_2_O_4_ nanoparticles (g-C_3_N_4_/ZnO: NiFe_2_O_4_ = 2:1, 1:1, and 1:2; namely, CZN1, CZN2, and CZN3, respectively). For comparison, another sample, PM1 (mixture of g-C_3_N_4_/ZnO and NiFe_2_O_4_ in 2:1 ratio), was also prepared. TEM images of the CZN1 ternary heterostructure revealed the uniform dispersion of ZnO and NiFe_2_O_4_ nanoparticles over the surface of g-C_3_N_4_ flaky sheets (Figure 14e). The photocatalytic performance of different synthesized photocatalysts (g-C_3_N_4_, ZnO, NiFe_2_O_4_, g-C_3_N_4_/ZnO, PM1, CZN1, CZN2, and CZN3) was investigated by studying the degradation of fluoroquinolone (FQ) antibiotics under visible-light irradiation. Among these, CZN ternary heterostructures showed enhanced photocatalytic activity, which was ascribed to the increased visible-light absorption capability, double Z-scheme charge-transfer mechanism, and reduced photoinduced electron–hole recombination rate. In addition, the superior photocatalytic performance of CZN1 to that of CZN2 and CZN3 could be ascribed to the increased amount of NiFe_2_O_3_. It is suggested that the increased amount of NiFe_2_O_3_ caused the inhibition of the photoexcitation of g-C_3_N_4_ and ZnO. Additionally, the magnetic properties of CZN ternary heterostructures were helpful for recovering the photocatalysts after their use with the help of an external magnet. A reusability test of CZN1 was carried out by degrading FQs for up to five cycles, which signified the substantial stability of the synthesized ternary composite. 

In accordance with the band structure of the CZN1 heterostructure, a direct dual Z-scheme charge-transfer mechanism has been proposed, as shown in Figure 14f. The reported CB/VB potentials of g-C_3_N_4_, ZnO, and NiFe_2_O_4_ are −1.12/+1.68 eV, −0.31/+2.89 eV, and −0.58/+1.08 eV, respectively. When light irradiates the CZN heterostructure, photogenerated electrons in the CB of ZnO migrate to the VBs of g-C_3_N_4_ and NiFe_2_O_4_ and combine with the photogenerated holes. This dual charge-transfer channel results in the effective separation of photogenerated electrons in the CBs of g-C_3_N_4_ and NiFe_2_O_4_ and photogenerated holes in the VB of ZnO. Further, these photogenerated electrons in the CBs of g-C_3_N_4_ and NiFe_2_O_4_ react with dissolved oxygen to generate O_2_^•−^ radicals, since the redox potential of O_2_/O_2_^•−^ is −0.33 eV. Similarly, the photogenerated holes in the VB of ZnO react with H_2_O or OH^−^ to generate OH^•^ radicals because the redox potentials of OH^•^/H_2_O and OH^•^/OH^−^ are +2.27 eV and +1.99 eV, respectively. Free-radical-trapping experiments using different scavengers validated that all three ROS were accountable for the photodegradation of FQs in the order O_2_^•−^ > h^+^ > OH^•^. Finally, it is believed that the fabricated heterostructure can provide a new avenue for designing multifarious components of innocuous systems for environmental remediation.

Recently, a nebula-like composite of oxygen-doped g-C_3_N_4_, ZnO, and TiO_2_@halloysite nanotubes (O-g-C_3_N_4_/ZnO/TiO_2_@HNTs) was prepared by Aghababaei et al. using simple calcination and sol–gel methods [196]. HNTs are natural clay silicate minerals with a unique shape, large surface area, and non-toxic nature and are easily disposed of and reused. These are promising materials for the support of nanoparticles to avoid their agglomeration. The photodegradation of diclofenac was evaluated utilizing the prepared photocatalyst under UV-light irradiation at pH 6.83. The significant photocatalytic activity was ascribed to the formation of a double Z-scheme heterojunction in which a more significant number of electrons and holes were generated due to the electrostatic interaction of O-g-C_3_N_4_/ZnO/TiO_2_ on HNTs. A slight decrease observed in the performance of the O-g-C_3_N_4_/ZnO/TiO_2_@HNTs composite over five successive cycles indicated its good stability. Moreover, the findings of photocatalytic scavenger experiments confirmed that holes and OH^•^ radicals were necessary ROS for the redox reactions.

## 8. Formation of g-C_3_N_4_/ZnO-Based S-Scheme Heterojunction Photocatalysts

**Figure 15:**
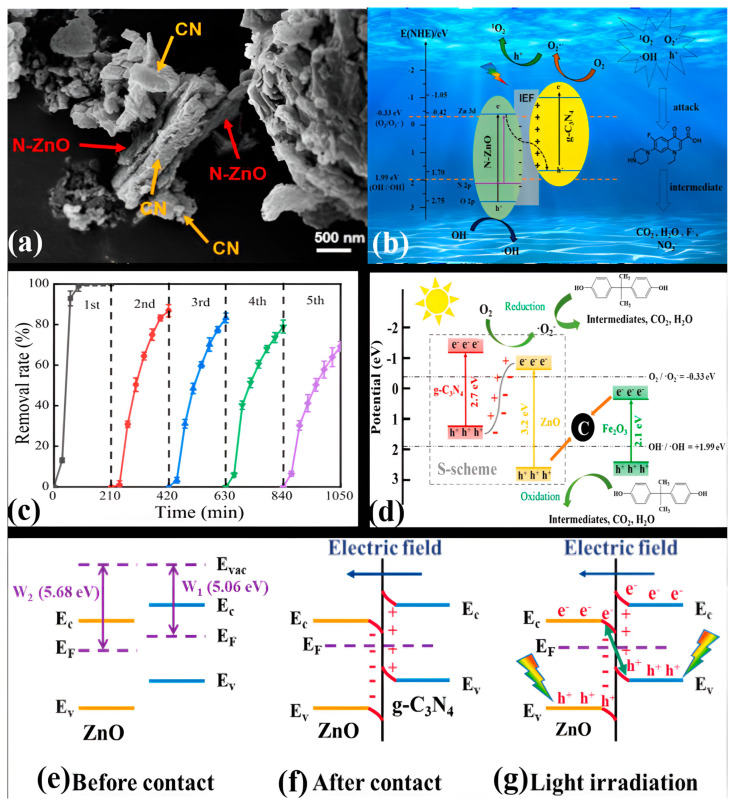
(**a**) SEM image of 15% NZCN and (**b**) the possible mechanism for NOR degradation in the 15% NZCN/vis system. (**c**) Removal rate of BPA by FZCCN-4 photocatalysts over five cycles. Condition: C_0_ (BPA) = 10 mg L^−1^; C_0_ (catalyst) = 0.8 g L^−1^. (**d**) Proposed photocatalytic mechanism in the FZCCN-4 photocatalytic system under visible-light irradiation. (**e**) Work function of ZnO (101) and g-C_3_N_4_ (001), (**f**) the internal electric field and band bending at the interface of ZnO@g-C_3_N_4_ after contact, and (**g**) transfer process of photogenerated carriers in S-scheme heterojunctions. Reprinted from Refs. [198,201,205] with permission from Elsevier.

To realize the advantages of S-scheme heterojunction photocatalysts (as mentioned in Section 5.2) over Z-scheme heterojunctions, several research efforts are being devoted to engineering and synthesizing g-C_3_N_4_/ZnO-based S-scheme heterojunction photocatalysts. Utilizing the advantages of an S-scheme heterojunction for the effective separation of photogenerated charge carriers and retaining the ability of a redox semiconductor, Liu et al. constructed a solar-driven g-C_3_N_4_/ZnO@PET (polyester fiber) composite by using a hydrothermal method [197]. The photocatalytic activity of the g-C_3_N_4_/ZnO@PET composite was assessed by degrading MB dye. It was found that the photocatalytic efficiency of the g-C_3_N_4_/ZnO@PET composite was significantly higher, including excellent recyclability for up to five consecutive cycles, than that of ZnO@PET and g-C_3_N_4_@PET, which was attributed to the S-scheme charge-transfer mechanism within the composite. In the recent past, Zhang et al. constructed 2D/2D N-doped ZnO/g-C_3_N_4_ (N-ZnO/CN) heterojunctions by calcining ZIF-L/g-C_3_N_4_ composites [198]. Different N-ZnO/CN composites were prepared by varying the mass ratios of ZIF-L and CN (5%, 15%, and 20%). The morphology study of 15% N-ZnO/CN by SEM analysis clearly showed that N-ZnO nanoparticles were wrapped by CN nanolayers. There was no sintering phenomenon that might be associated with the CN intercalation between N-ZnO (Figure 15a). The photocatalytic performance of the as-prepared photocatalysts was evaluated by the photodegradation of norfloxacin (NOR) under visible-light irradiation, where the degradation efficiency of the 15% N-ZnO/CN composite was higher compared to the others. The higher efficiency was attributed to the excellent visible-light-capturing capacity due to the presence of an intermediate energy level (N 2p defect state) in N-ZnO and the efficient separation of photogenerated charge carriers and their migration within the heterojunction.

The energy band structure and free-radical-trapping experiments demonstrated the charge-transfer mechanism in the 15% N-ZnO/CN composite. Since the composite was composed of two n-type semiconductors, an S-scheme charge-transfer mechanism was proposed (Figure 15b). When this composite was irradiated with visible light, photogenerated electrons and holes were generated in the CB and VB of N-ZnO and CN, respectively. The photogenerated electrons in the CB of N-ZnO migrated to the VB of CN and combined with the photogenerated holes. As a result, the photogenerated holes in the VB of N-ZnO and the photogenerated electrons in the CB of CN were effectively separated. Then, the holes in the VB of N-ZnO (VB position = +2.13 eV or +2.75 eV) reacted with OH^−^ to produce OH^•^ radicals (OH^−^/OH^•^ = +1.99 eV vs. NHE), while the electrons in the CB of CN (CB position = −1.05 eV) combined with O_2_ to produce O_2_^•−^ radicals (O_2_/O_2_^•−^ = −0.33 eV vs. NHE). Finally, these ROS effectively degraded NOR. However, holes and O_2_^•−^ radicals were the dominant species for the photocatalytic redox reaction. A novel S-scheme heterojunction photocatalyst was prepared by Kim’s group. Following the hydrothermal method, a g-C_3_N_4_- and rGO-combined Ag-deposited ZnO (g-C_3_N_4_/rGO/ZnO-Ag) heterostructure nanocomposite was prepared [199]. The photocatalytic activity of the as-prepared nanocomposite was evaluated using a mixture solution of Rh B and MB dyes under visible-light irradiation. Due to high light absorption ability and enhanced charge separation efficiency, the S-scheme g-C_3_N_4_/rGO/ZnO-Ag heterostructure nanocomposite exhibited improved photodegradation activity. Also, the rGO nanosheet could act as an electron mediator that could prevent the recombination of photogenerated electrons/holes, and Ag nanoparticles deposited on the ZnO surface could boost the visible-light absorption and increase the electron density on its surface via the SPR effect. Moreover, Ag nanoparticles acted as electron transportation vehicles toward ZnO, which could separate electron–hole pairs in g-C_3_N_4_ effectively.

Among the various efforts for improving the photocatalytic performance of the S-scheme system, Lee et al. reported a template material included in an S-scheme g-C_3_N_4_/ZnO heterojunction photocatalyst. They used zeolite imidazolate framework-8 (ZIF-8) as a template for g-C_3_N_4_ and a precursor of ZnO [200]. Another exemplary work was presented by Zhang’s group. A series of Fe_2_O_3_-ZnO@C/g-C_3_N_4_ (FZCCN) heterojunction photocatalysts were prepared following precipitation and the calcination process [201]. Based on the theoretical mass ratios of ZnO and g-C_3_N_4_, the different samples were named FZCCN-1, FZCCN-2, FZCCN-3, FZCCN-4, and FZCCN-5. For comparison, Fe_2_O_3_@C, ZnO@C, and Fe_2_O_3_-ZnO@C samples were also prepared under similar conditions. The photocatalytic activity of all samples was verified by degrading BPA under visible-light irradiation. Compared to other samples, FZCCN heterojunctions achieved higher removal rates. The FZCCN-4 heterojunction even exhibited a 100% BPA removal rate at 60 min, which was attributed to the enhanced visible-light absorption, effective charge separation and transfer through the heterojunction structure inside the composite, and the larger specific surface area of FZCCN-4. Reusability and stability tests of the FZCCN-4 heterojunction showed a decrease in the BPA removal rate from 100% to 69.3% at the end of the fifth cycle (Figure 15c). An Inductively Coupled Plasma Emission Spectrometer (ICP) showed the dissolution of metal oxides prepared in the synthesis process during the photodegradation reaction, which affected the degradation efficiency. However, the morphology and the surface functional groups of the FZCCN-4 photocatalyst remained the same.

On the basis of previously reported band positions and band structures, an S-scheme charge-transfer mechanism is proposed for FZCCCN-4 heterojunction (Figure 15d). The VB/CB positions of g-C_3_N_4_ are +1.58/−1.12 eV. The VB/CB potentials of ZnO and Fe_2_O_3_ are +2.70//−0.50 eV and +2.43/+0.33 eV, respectively. It is suggested that the C element doped in ZnO could act as an electron trapper and facilitate the movement of photogenerated electrons from the CB of Fe_2_O_3_ to the VB of ZnO to combine with photogenerated holes. Meanwhile, due to the formation of the S-scheme heterojunction, the photogenerated electrons in the CB of ZnO migrated to the VB of g-C_3_N_4_ to combine with photogenerated holes. Considering the redox potentials of OH^−^/OH^•^ (+1.99 eV) and O_2_/O_2_^•−^ (−0.33 eV), the formation of O_2_^•−^ radicals, rather than the formation of OH^•^ radicals, was possible in the photocatalytic reaction system. The ROS-trapping experiments confirmed that the O_2_^•−^ radical was the main active ROS for the degradation process. Another recent progress was reported by Khataee’s group, who synthesized a g-C_3_N_4_/ZnO S-scheme heterojunction composite using the calcination method [202]. The photocatalytic activity of the synthesized composite was tested for the photodegradation of crystal violet (CV) dye under UV-light irradiation. H_2_O_2_ was also added to the reaction system to promote the formation of OH^•^ radicals. To rectify the issues of the plasma system, a new hybrid system was recently reported by Feyzi et al. to remove pharmaceutical pollutants from effluents [203].

A cobalt-nanoparticle-deposited ZnO/g-C_3_N_4_ S-scheme heterojunction photocatalyst (ZnO/Co/g-C_3_N_4_) was synthesized using ultrasonic and sol–gel methods [204]. Various dyes were used to evaluate the photocatalytic activity of the ZnO/Co/g-C_3_N_4_ heterojunction in the presence of visible light and sunlight. The metallic Co nanoparticles not only facilitated the electron transport via the SPR effect but also increased the visible-light response of the photocatalyst. Ternary nanocomposites of ZnO, g-C_3_N_4_, and zeolite were prepared with different ratios. Different characterization techniques revealed a suitable specific surface area, crystallinity, and heterojunction formation in all nanocomposites. TC, a pharmaceutical pollutant, was used to investigate the photocatalytic activity of the prepared samples. All of the degradation experiments were carried out in a water falling film dielectric barrier discharge (WFFDBD) treatment system. To carry out the experiments, the prepared nanocomposites were loaded on a constructed plasma reactor provided with a water falling film facility. Among different samples, the sample with 60 wt% ZnO, 30 wt% g-C_3_N_4_, and 10 wt% zeolite (Zn60CN30-Z) showed higher TC degradation performance in the hybrid plasma reactor system (Zn60CN30-Z/WFFDBD) compared to the WFFDBD system alone. The higher performance of the Zn60CN30-Z/WFFDBD hybrid system was favored by the synergistic effect of the Zn60CN30-Z nanocomposite and WFFDBD system. Importantly, the creation of an S-scheme type heterojunction and the electric field generated between ZnO and g-C_3_N_4_ also helped to prevent the fast recombination of photogenerated charge carriers. A slight decrease of 7% in TC degradation performance after the fourth cycle suggests the satisfactory stability of the Zn60CN30-Z nanocomposite. Finally, scavenger addition confirmed the important role of holes and OH^•^ radicals in the degradation process.

For the alleviation of the recovery problem and secondary pollution of the photocatalyst, the fabrication of a photocatalytic membrane using a photocatalyst can have high application potential. Such a photocatalytic membrane can make full use of photocatalytically active sites and possess self-cleaning properties under continuous-operation conditions. Inspired by these advantages, Shi et al. prepared ZnO@g-C_3_N_4_ composite membranes via vacuum-assisted filtration, followed by in situ growth methods [205]. Different composite membranes were prepared via the in situ growth of ZnO crystals in the g-C_3_N_4_ membrane by adding different amounts of Zn(NO_3_)_2_. The prepared ZnO@g-C_3_N_4_ composite membranes were named CNZ-x (where x is the mass of Zn(NO_3_)_2_, i.e., 0.1, 0.15, 0.25, and 0.5 g). The MB dye removal performance of all composite membranes was evaluated in a dynamic recirculation filtration system under visible-light irradiation. Compared to all others, the CNZ-0.15 g sample showed higher MB dye removal efficiency and a water flux of 336.8 L• m^−2^ • bar^−1^ • h^−1^. It was concluded that sufficient adsorption sites on the composite and the formation of a heterojunction between ZnO and g-C_3_N_4_ could enhance the removal percentage of the dye. The formation of the heterojunction facilitated the separation of photogenerated electron–hole pairs, improving the photocatalytic activity and MB dye removal efficiency. However, the increased loading of ZnO occupied the adsorption sites and heterojunction structures, which weakened the photocatalytic activity. Also, CNZ-0.15 g showed higher self-cleaning performance and stability/reusability after three cycles. Finally, it is believed that the as-prepared photocatalytic membrane (CNZ-0.15 g) can find practical application in the continuous removal of organic pollutants.

Combined with the energy band arrangement, an S-scheme electron transfer mechanism was speculated for the ZnO/g-C_3_N_4_ heterojunction (Figure 15e–g). The calculated CB, VB, and work function of g-C_3_N_4_ are −0.92 eV, +1.78 eV, and 5.06 eV, respectively. Similarly, the calculated CB, VB, and work function of ZnO are −0.55 eV, +2.65 eV, and 5.68 eV, respectively. Due to its higher CB and VB and lower work function, g-C_3_N_4_ behaves as an RP. Contrastingly, ZnO behaves as an OP due to its lower CB and VB and higher work function. Upon contact, the Fermi levels of g-C_3_N_4_ and ZnO moved downward and upward, respectively, until they met at the same level. At the same time, electrons in g-C_3_N_4_ spontaneously transferred to ZnO, which formed depletion and accumulation layers at the interface. As a result, an electric field was formed at the g-C_3_N_4_-ZnO interface due to the accumulation of positive charges on g-C_3_N_4_ and negative charges on ZnO. At last, the g-C_3_N_4_ band bent upward due to electron consumption, and the ZnO band bent downward due to electron accumulation. Under visible-light irradiation, the photogenerated electrons in the CB of ZnO combined with the photogenerated holes in the VB of g-C_3_N_4_ due to band bending and the coulombic force of attraction. In addition, the photogenerated electrons that accumulated in the CB of g-C_3_N_4_ with a strong reduction ability reacted with dissolved oxygen to produce O_2_^•−^ radicals. On the other hand, the photogenerated holes that accumulated in the VB of ZnO with a strong oxidation ability reacted with water to produce OH^•^ radicals. In this way, the ROS produced from well-separated electrons/holes could degrade MB dye. Radical capture experiments confirmed that O_2_^•−^ and OH^•^ radicals were the main ROS in the photocatalytic degradation process.

Recently, a simple solution combustion approach was applied to design a ternary ZnO-g-C_3_N_4_-CuO heterojunction photocatalyst for degrading MB dye and Rh B dye under visible light [206]. Compared to ZnO, ZnO-CuO, and ZnO-g-C_3_N_4_, the ternary ZnO-g-C_3_N_4_-CuO heterojunction composite showed higher degradation performance, which is attributed to the dual S-scheme at the interface of the ZnO-g-C_3_N_4_-CuO heterojunction composite. Due to the formation of the dual S-scheme transfer mode, photogenerated electrons in the CBs of ZnO and CuO combined with the holes in the VB of g-C_3_N_4_, resulting in outstanding separation of photogenerated electrons in the CB of g-C_3_N_4_ and photogenerated holes in the VBs of ZnO and CuO. Furthermore, the photogenerated electrons and holes reacted with oxygen and water to generate O_2_^•−^ and OH^•^ radicals, respectively, which are responsible for the photodegradation of organic dyes. Studies on the photodegradation of organic pollutants by g-C_3_N_4_/ZnO-based double Z-scheme and S-scheme heterojunction photocatalysts are summarized in Table 5.

## 9. Conclusions and Future Perspective

In general, g-C_3_N_4_/ZnO-based Z-scheme and S-scheme heterojunction photocatalysts are more advantageous in the field of visible-light-assisted photocatalysis. Owing to its low synthesis cost, stable physiochemical properties, suitable band gap structure, and tunable properties using simple strategies, g-C_3_N_4_ has been explored as a metal-free and visible-light-responsive photocatalyst, but the photocatalytic performance of pristine g-C_3_N_4_ is poor due to the high recombination rate of photogenerated electron–hole pairs. Amongst the different metal oxide semiconductor photocatalysts, ZnO is receiving immense attention these days, as it is a self-driven semiconductor photocatalyst with unique and fascinating properties, like cost-effectiveness, environmental friendliness, and high quantum efficiency. However, the band edge of ZnO lies in the UV region, which makes it inactive under visible-light irradiation. Therefore, the formation of Z-scheme or S-scheme heterojunctions between g-C_3_N_4_ and ZnO makes the resultant photocatalysts preferred forms for photocatalytic applications. Hence, this review presents a comprehensive summary of various synthesis strategies of g-C_3_N_4_/ZnO-based Z-scheme and S-scheme heterojunction photocatalysts having high visible-light absorption properties, effective charge migration/separation ability, and enhanced photocatalytic activity with reusability. Also, strategies to improve the photodegradation efficiency of these heterojunctions via the formation of binary and ternary composites with or without metal/non-metal doping and codoping are discussed. Additionally, a discussion about the crystal structures and band structures of g-C_3_N_4_/ZnO is included, along with the charge-transfer mechanism within the heterojunction. Although g-C_3_N_4_/ZnO-based Z-scheme/S-scheme heterojunctions are emerging as photocatalysts for the removal of organic pollutants from wastewater, there are still many challenges that need to be addressed before widening their practical applications.

i. The majority of the reports have shown that g-C_3_N_4_ can be facilely synthesized by the thermal polymerization of nitrogen-rich precursors. But the resultant g-C_3_N_4_ with a bulky structure is disadvantageous to the photocatalytic efficiency due to a low surface area, limited surface reactive sites, and the inadequate utilization of visible light. These shortcomings can be alleviated by selecting appropriate g-C_3_N_4_ precursors, optimizing the reaction temperature and condensation period, applying an exfoliation-assisted strategy, and following template-free methods to obtain highly porous g-C_3_N_4_ and controlled morphology.
ii. Many studies in the literature have reported that g-C_3_N_4_ nanosheets act as anchoring sites for ZnO nanostructures or other components to form nanocomposites. But the contact of nanostructures on the surface of g-C_3_N_4_ and their uniform distribution without aggregation on the surface of g-C_3_N_4_ are very challenging. Consequently, an appropriate heterojunction interface between g-C_3_N_4_ and nanostructures might not be formed, which hinders the effective charge transport/separation. Therefore, functionalization of the g-C_3_N_4_ surface with specific functional groups could be the best alternative for strengthening the anchoring ability of g-C_3_N_4_ and enhancing the light absorption properties of heterojunctions.
iii. Photocatalytic activities of g-C_3_N_4_/ZnO-based heterojunction photocatalysts have thus far mainly been used for the photodegradation of organic contaminants in laboratory samples. Hence, further studies need to focus on real water samples (i.e., from the laboratory to the real field).
iv. As discussed in this review, the visible-light response, redox ability, electron–hole mobility, and surface dynamic heterostructure at the interface of g-C_3_N_4_/ZnO-based Z-scheme/S-scheme heterojunction photocatalysts can be increased by metal/non-metal doping or forming ternary composites with g-C_3_N_4_ and ZnO. In addition, MXenes (2D few-atom-thick layers of transition-metal carbides and nitrides) could be promising alternatives to form ternary composites. Cost-effective MXenes possessing high conductivity can function as electron sinks, which accelerate the migration of photogenerated charge carriers and their effective separation.
v. The charge-transfer mechanism is the key basis for understanding the likely reaction process occurring on the surface of a photocatalyst. However, in the case of g-C_3_N_4_/ZnO-based heterojunction systems, the charge-transfer mechanism seems inconsistent due to the variation in the reported CB/VB potentials of g-C_3_N_4_ and ZnO. Therefore, extensive studies should be focused on the DFT, radical-trapping tests, XPS analysis, etc., to understand the exact mechanism.

## Figures and Tables

**Table 1:** Photodegradation of organic pollutants using g-C_3_N_4_/ZnO-based all-solid-state Z-scheme heterojunction photocatalysts.

Photocatalysts/Dosage	Synthesis Method	Light Used	Organic Pollutant	Performance	Ref.
ZnO/Fe_2_O_3_/g-C_3_N_4_ heterojunction/ (25 mg in 50 mL)	Hydrothermal treatment, followed by low-temperature calcination	Visible and sunlight	Sulfamethazine (5 mg L^−1^)	100% in 8 h	[127]
3Dg-C_3_N_4_/ZnO@graphene aerogel (g-C_3_N_4_/ZnO@GA)30% heterojunction formed with 30 wt% g-C_3_N_4_/ (5 mg in 25 mL)	Hydrothermal self-assembly combined with freeze-drying	UV and visible light	Rh B, MO, MV, and MB dyes (20 mg L^−1^)	Rh B dye in 120 min UV light: 81% Visible light: 82.7% MO dye in 120 min UV light: 50% Visible light: 46.9%	[128]
ZnO/g-C_3_N_4_/RGO photocatalyst/ (25 mg in 50 mL)	Calcination	Visible light and UV light	Deoxynivalenol (10 ppm)	Visible light: 90% in 5 h UV light: 90% in 120 min	[129]
Ag_3_PO_4_/g-C_3_N_4_/ZnO ternary composite/ (0.6 g/L, 50 mL)	Ultrasound-assisted precipitation	Visible light and sunlight	Tetracycline hydrochloride (30 mg/L)	Visible light: 88.48% Sunlight: 89.95% in 120 min	[130]
ZnO/C/g-C_3_N_4_ composite ENFs (containing 0.25 wt% g-C_3_N_4_)/ (10 mg in 10 mL)	Electrospinning and annealing under N_2_ atmosphere	Simulated sunlight	MB dye (10^−5^ M)	91.8% in 120 min	[136]
Cu-doped ZnO/Cu/g-C_3_N_4_ heterostructure (g200: prepared by adding 0.02 g of prepared g-C_3_N_4_)/(0.4 g/L for MB and 0.5 g/L for Rh B dye)	Calcination–hydrothermal	Sunlight	MB dye and Rh B dye (0.01 g in 100 mL)	MB dye: 98% in 20 min Rh B dye: 99% in 60 min	[137]
P-laden biochar/ZnO/g-C_3_N_4_ core–shell composite [Pbi-ZnO-g-C_3_N_4_ (50)], in which mass% of g-C_3_N_4_ to Pbi-ZnO is 50)/ (1 g in 100 mL)	Thermal polymerization, copyrolysis, and annealing under N_2_ atmosphere	Simulated sunlight	Atrazine (10 mg/L)	85.3% in 260 min	[138]
Nanocomposite of (NiCo/ZnO/g-C_3_N_4_)/ (20 mg in 100 mL)	Thermal condensation, in situ hydrothermal treatment, ultrasonic decomposition	Visible light	Oxytetracycline and tetracycline (10 mg/L)	Oxytetracycline: 71.3% Tetracycline: 81.29% in 50 min	[139]
g-C_3_N_4_//ZnO/Fe_2_O_3_ ternary composite/ (50 mg in 50 mL, pH 11)	Calcination	Visible light	MB dye (30 mL/L)	94% in 120 min	[140]

**Table 2:** Photodegradation of organic pollutants using binary g-C_3_N_4_/ZnO-based direct Z-scheme heterojunction photocatalysts.

Photocatalysts/Dosage	Synthesis Method	Light Used	Organic Pollutant	Performance	Ref.
ZnO nano-triangle@g-C_3_N_4_ nanofoils (20%)/(1.25 g/L)	Sono-chemical impregnation	Solar light	Rh B dye (10 ppm)	100% in 60 min	[141]
g-C_3_N_4_ (10 wt%)/O-defective ZnO (OD-ZnO) nanorods/ (0.1 g/100 mL)	Solution conversion, heating, and ultrasonication	Visible light	4-CP (10^−4^ mol L^−1^)	95% in 60 min	[142]
2D/3D g-C_3_N_4_@ZnO heterostructure/(0.3 g/L)	Thermal atomic layer deposition	Simulated sunlight	Cephalexin (10 mg/L)	98.9% in 60 min	[143]
g-C_3_N_4_/ZnO composite@500 °C (CNZ-500)/(10 mg/L)	Thermal treatment	Visible light	MB dye (10 mg/L)	Rate constant: 2.88 × 10^−2^ min^−1^	[144]
ZnO/g-C_3_N_4_ composite (2 g/L)	One-step calcination	Visible light	MB dye (10 ppm)	98.83 in 60 min	[145]
ZnO/g-C_3_N_4_ nanocomposite (7 g-C_3_N_4_ /ZnO: 7 wt% of g-C_3_N_4_ relative to ZnO)/(0.1 g in 400 mL)	Electrostatic self-assembly combined with low-temperature precipitation	Simulated sunlight	MB dye (10 mL/L)	93% in 5 h	[146]
0D/2D g-C_3_N_4_ quantum dots/ZnO with oxygen vacancies [(CNQDs/OV-ZnO)]/ (10 mg in 80 mL)	Mixing and calcination	Visible light	MB dye and BPA (10 mL/L)	MB dye: 71% in 4 h and BPA: 61% in 12 h	[147]
Dicyanadiamine-derived core–shell composite of g-C_3_N_4_/ZnO (DCDA-CNZ)/ (10 mg in 50 mL)	Thermal polymerization	Visible light	MB dye (10 ppm)	Rate constant: 2.39 × 10^−2^ min^−1^	[148]
g-C_3_N_4_/ZnO nanocomposite/ (50 mg in 100 mL)	Calcination	Sunlight UV light	MB dye (1 × 10^−5^ mol/L)	Sunlight 100% UV light <100% (in 60 min)	[149]
g-C_3_N_4_/ZnO composite formed by 1:30 precursor mass ratio/ (100 mg in 100 mL)	Co-melting-recrystallization	Visible light	MO dye and levofloxacin (10 mg L^−1^)	MO dye: 62% in 240 min Levofloxacin: 66.7% in 210 min	[150]
g-C_3_N_4_/ZnO heterojunction/ (50 mg in 60 mL)	Thermal decomposition	Visible light	MO dye (2 × 10^−5^ M)	Rate constant: ~0.0117 min^−1^	[151]
g-C_3_N_4_/ZnO heterostructure/ (0.1 g in 50 mL)	Exfoliation process	Visible light	MG dye (10 ppm)	84.3% in 60 min	[152]
75 wt% ZnO/g-C_3_N_4_ nanocomposite/(0.1 g in 100 mL)	Pyrolysis and hydrothermal	Visible light	MB dye (50 mg/L)	98% in 120 min	[153]
ZnO-g-C_3_N_4_ nanocomposite/ (25 mg in 50 mL)	Sol–gel-assisted route	Visible light	Congo red (10 mg/L)	More than 80% in 30 min	[154]
ZnO doped with 20% g-C_3_N_4_ (ZnO-g-C_3_N_4_-20) nanosheet/ (50 mg in 50 mL)	Hydrothermal	Visible light	MO dye (20 mg.dm^−3^)	98.5% in 150 min	[155]
g-C_3_N_4_/ZnO composite/ (20 mg in 100 mL)	Hydrothermal	Solar irradiation	MB dye, Rh B dye and Ciprofloxacin (10 ppm)	MB dye: 100% in 50 min Rh B dye: 98% in 100 min Ciprofloxacin: 96% in 180 min	[156]
ZnO/g-C_3_N_4_ heterojunction (50-Zn/gCN)/ (25 mg in 250 mL)	Mixing, sonication, and thermal treatment	Simulated solar light	CFZ, and RB5 dye/(10 mg L^−1^)	CFZ: 78% RB5 dye: 95% (in 120 min)	[157]
g-C_3_N_4_/ZnO heterojunction/ (100 mg in 50 mL)	Impregnation and ultrasonic treatment	Visible light	Rh B dye (10 ppm)	75% in 250 min	[158]
ZnO-g-C_3_N_4_ heterostructure/ (40 mg in 100 mL)	Solution mixing	Visible light	MB dye (5 ppm)	91.5% in 120 min	[159]
40% ZnO-g-C_3_N_4_ lotus bud-like composite/ (100 mg in 250 mL)	One-pot pyrolysis	Visible light	Benzene (20 mg/L)	98.6% in 270 min	[160]
ZnO/g-C_3_N_4_ composite/ (50 mg in 50 mL, pH 9)	Calcination	Visible light	MB dye, MG dye, and MO dye (10 mg/L)	MB dye: 99.16% MG dye: 96.42% MO dye: 57.57% (in 180 min)	[161]
g-C_3_N_4_/ZnO composite/ (0.05 g in 150 cm^−3^)	Annealing	UV light	Acid orange 7 (25 mg in dm^−3^)	51.5% in 120 min	[162]

**Table 3:** Photodegradation of organic pollutants using ternary g-C_3_N_4_/ZnO-based direct Z-scheme heterojunction photocatalysts.

Photocatalysts/Dosage	Synthesis Method	Light Used	Organic Pollutant	Performance	Ref.
g-C_3_N_4_/AgBr/ZnO 30 ternary composite (30 is the wt. ratio)/ (0.04 g in 100 mL)	Sonication-assisted deposition technique	Visible light	MB dye (5 mg L^−1^)	96.3% in 80 min	[163]
Ag/g-C_3_N_4_/ZnO nanorod-nanocomposite (0.089 g/L)	Solvothermal, polycondensation, stirring	Visible light	Paracetamol, Cefalexin, and Amoxicillin (40 mg/L)	Paracetamol: 85.3% Cefalexin: 71.74% Amoxicillin: 41.36% in 180 min	[164]
α-Fe_2_O_3_ decorated g-C_3_N_4_/ZnO (g-C_3_N_4_/ZnO@α-Fe_2_O_3_) ternary nanocomposite/ (0.05 g in 100 mL)	Direct pyrolysis and sol–gel	Visible light	Tetrazine dye (10 mg L^−1^)	99.34% in 35 min	[165]
CdS@ZnO-g-C_3_N_4_ ternary nanocomposite/ (1 g L^−1^ at pH 6)	One-pot room-temperature ultrasonic route	UV light and visible light	Rh B dye (1 × 10^−5^ M)	UV light: 93.34% in 120 min Visible light: 90% in 180 min	[166]
Ag/Ag_2_O combined g-C_3_N_4_/ZnO (g-C_3_N_4_/ZnO-Ag_2_O) ternary composite/ (50 mg in 100 mL)	Calcination and hydrothermal	Visible light	MB dye (30 ppm) 4-CP (10 ppm)	MB dye: 96.5% 4-CP: 85.7% in 120 min	[167]
55% g-C_3_N_4_@Ag-ZnO hybrid nanocomposite/ (0.01 g in 100 mL)	Physical mixing	Solar light	MB dye (100 mg in 100 mL)	98% in 80 min	[168]
MoS_2_/g-C_3_N_4_/ZnO ternary nanocomposite/ (0.1 g in 50 mL)	Hydrothermal and exfoliation	Visible light	MG dye (10 ppm)	97% in 60 min	[169]
Mg-ZnO/g-C_3_N_4_@ZIF-8 multicomponent nanocomposite/((0.5 g/L) in presence of NaBH_4_ at pH 9)	Chemical precipitation	Visible light	Illicit drug (50 mg/L)	100% in 10 min	[170]
g-C_3_N_4_-ZnO/BiOBr heterojunction photocatalyst/ (0.05 g in 1 L)	Hydrothermal	UV light	MO dye (20 mg/L)	99.26% in 130 min	[171]

**Table 4:** Photodegradation of organic pollutants using metal/non-metal-doped g-C_3_N_4_/ZnO-based direct Z-scheme heterojunction photocatalysts.

Photocatalysts/Dosage	Synthesis Method	Light Used	Organic Pollutant	Performance	Ref.
Cu-doped ZnO/g-C_3_N_4_ photocatalyst/ (0.5 g in 500 mL)	Autoclave heating and calcination	Visible radiation	Atrazine (100 ppm)	90% in 180 min	[174]
Sr-ZnO/g-C_3_N_4_ heterojunction/(0.5 g in 80 mL)	One-pot facile method	UV-vis irradiation	Methylene green dye (10 mg/L)	96% in 20 min	[175]
g-C_3_N_4_/(Cd-ZnO) nanocomposite/ (0.01 g in 188 mL water and 12 mL dye solution)	Co-precipitation	Visible light	MB dye (10 mg in 100 mL water)	95% in 90 min	[176]
Ni/ZnO/g-C_3_N_4_ nanocomposite [3% Ni/ZnO,70% g-C_3_N_4_ (NiZG-70)]/ (200 mg in 200 mL)	Chemical co-precipitation	Sunlight	MB dye (10 mg L^−1^)	100% in 70 min	[177]
Cu-doped ZnO/g-C_3_N_4_ composite/ (100 mg in 200 mL)	Hydrothermal treatment followed by calcination	Visible light	CIP (5 mg/L)	95% in 360 min	[178]
Al/Ga-codoped ZnO/g-C_3_N_4_ heterojunction (AGZ/CN 560: where g-C_3_N_4_ was prepared at 560 °C)/(20 mg in 30 mL)	Thermal decomposition and single-phase dispersion	Visible light	MB dye (10 mg/L)	95.4% in 150 min	[179]
Ru-ZnO@g-C_3_N_4_ mesoporous nanocomposite/ (5 mg in 100 mL, pH 10)	Ultrasonic technique	UV light	MB dye (30 ppm)	92.2% in 60 min	[180]
Hybrid g-C_3_N_4_/ZnO-W/Co_(0.010)_ heterojunction/(0.05 mg)	Precipitation method	Visible light	MB dye (10 ppm)	90% in 90 min	[181]
C-doped g-C_3_N_4_ grafted on C, N-codoped ZnO (BT-CCN@ZnO) microflowers/	Bio-templated hydrothermal	Simulated solar irradiation	BPA	92.5% in 180 min	[182]
ZnO-embedded S-doped g-C_3_N_4_ (ZnO-SCN) heterojunction/ (50 mg in 100 mL)	Sol–gel-assisted calcination	Visible light	MB dye and Rh B dye (10 ppm)	93% in 80 min	[183]
ZnO-coupled F-doped g-C_3_N_4_ (Fe@g-C_3_N_4_/ZnO) heterojunction/(50 mg)	Simple wet-chemical	UV-vis and direct sunlight	Rh B dye (10 ppm)	(In 75 min) UV-vis light: 97% Direct sunlight: 98%	[184]
P, C-GCN/15 wt% SiO_2_/5 wt% ZnO (PC-GCN/15-SiO_2_/5-ZnO) heterogeneous nanocomposite/[100 mL (500 mg L^−1^)]	Calcination	LED 200 W	MB dye (20 mg L^−1^)	100% in 90 min	[185]
N-doped ZnO/g-C_3_N_4_ core–shell nanoplates with 5 wt% loaded g-C_3_N_4_ (CNZON5)/ (0.025 g in 100 mL)	Ultrasonic dispersion	Visible light	Rh B dye (5 mg L^−1^)	Rate constant: 0.0679 min^−1^	[186]
N-ZnO/g-C_3_N_4_ composite/ (0.1 g in 100 mL)	High-temperature calcination	Visible radiation	MB dye (20 mg/L)	95% in 90 min	[187]
ZnO/g-C_3_N_4_ with N dopant (nitrogen-rich ZnO/g-C_3_N_4_ composite)/(100 mg)	Rotation-evaporation and calcination route	Visible light	NO gas (600 ppb)	More than 87% in 6 min	[188]
C-doped ZnO@ g-C_3_N_4_ composite with ZnO loading 50% (Zn-50)/ (0.1 g in 50 mL)	Thermal treatment	Visible light	Methyl green dye (20 mg/L)	98% in 60 min	[189]
C, N-codoped ZnO modified B-doped g-C_3_N_4_ [CNZ/BCN (1:1)] nanocomposite/ (30 mg in 20 mL)	In situ calcination	Simulated solar light	CIP (20 ppm)	86.7% in 60 min	[190]

**Table 5:** Photodegradation of organic pollutants using g-C_3_N_4_/ZnO-based double Z-scheme and S-scheme heterojunction photocatalysts.

g-C_3_N_4_/ZnO-Based Double Z-Scheme Heterojunction Photocatalysts					
Photocatalysts/Dosage	Synthesis Method	Light Used	Organic Pollutant	Performance	Ref.
CuO/ZnO/g-C_3_N_4_ ternary heterostructure/ (0.04 g in 100 mL)	Solution combustion route	Visible light	MB dye (10 mg/L)	98% in 45 min	[191]
O-g-C_3_N_4_/Zn_2_SnO_4_N/ZnO heterojunction/ (50 mg in 50 mL)	UV-light irradiation	Visible light	Rh B dye (5 mg L^−1^)	96% in 60 min	[192]
CuO-ZnO@g-C_3_N_4_ nanocomposite/(0.2 g/L with trace amount of H_2_O_2_ (250 ppm))	Ultrasound-assisted hydrothermal	Visible light + ultrasonic wave	Dibenzothiophene (DBT) 250 ppm	99.1% in 60 min	[193]
CuO nanoparticles and ZnO nanorods co-anchored on g-C_3_N_4_ nanosheets (CZ@T-GCN)/ (0.9 g L^−1^ in 250 mL)	Isoelectric-point-mediated method	Simulated sunlight	AMOX (60 mg L^−1^)	100% in 120 min	[194]
g-C_3_N_4_/ZnO-NiFe_2_O_4_ (weight ratio of CN/ZnO: NiFe_2_O_4_ is 2:1) [CZN1]/(0.5 g/L)	Simple sonication–calcination strategy	Visible light	Levofloxacin: 30 Ofloxacin: 15 Ciprofloxacin: 10 (mg/L)	Levofloxacin: 90% Ofloxacin: 88% Ciprofloxacin: 82% (in 90 min)	[195]
[(O-g-C_3_N_4_)/ZnO-TiO_2_@HNTs]/ (1 g in 40 mL)	Calcination and sol–gel	UV light	Diclofenac (10 mg/L)	100% in 50 min	[196]
g-C_3_N_4_/ZnO-based S-scheme heterojunction photocatalysts					
ZnO/g-C_3_N_4_@PET composite/ (6 cm × 3 cm in 50 mL)	Hydrothermal	Visible light	MB dye (15 mg L^−1^)	92.5% in 120 min	[197]
2D/2D N-ZnO/g-C_3_N_4_ composite (15% NZCN)/(0.2 g L^−1^)	Calcination, ultrasonication, and self-assembly	Visible light	Norfloxacin (5 mg L^−1^)	96.4% in 90 min	[198]
g-C_3_N_4_/rGO/ZnO-Ag heterostructure nanocomposite/ (60 mg in 100 mL)	Hydrothermal	Visible light	Mixed dye (Rh B + MB) (40 ppm)	90.4% in 100 min	[199]
g-C_3_N_4_/ZnO-450 heterojunction composite/ (5 mg in 50 mL)	ZIF8 template	Visible light	AR1 (10 mg/L)	95% in 1 h	[200]
Fe_2_O_3_-ZnO@C/g-C_3_N_4_ (FZCCN-4) heterojunction/ (0.8 g in 250 mL)	Precipitation and calcination	Visible light	BPA (10 mg/L)	100% in 60 min	[201]
g-C_3_N_4_/ZnO heterojunction composite/(0.10 g in 100 mL)	Calcination	UV light	CV dye (10 mg/L)	95.9% in 120 min	[202]
ZnO/g-C_3_N_4_/zeolite nanocomposite	Hydrothermal	Plasma discharge	TC (50 ppm)	95.5% in 100 min	[203]
g-C_3_N_4_/Co/ZnO heterojunction nanocomposite/ (20 mg in 100 mL)	Ultrasonic and sol–gel	Visible light and Sunlight	MB, CV, Rh B dyes (15 ppm) Rh B dye (15 ppm)	MB dye: 96.3% CV dye: 74.5% Rh B dye: 75.4% in 80 min 91.5% in 80 min	[204]
ZnO@g-C_3_N_4_ composite membrane (size: 10.75 cm^−2^) (Water flux: 336.8 L• m^−2^ • bar^−1^ • h^−1^)	Vacuum-assisted filtration and in situ growth	Visible light	MB dye (5 mg/L)	94.4% in 150 min	[205]
g-C_3_N_4_-ZnO-CuO heterojunction photocatalyst/ (20 mg in 30 mL)	Simple solution combustion approach	Visible light	MB dye and Rh B dye (10 mg/L)	MB dye: 100% Rh B dye: 90% (in 35 min)	[206]

## Acronyms

0D	Zero-dimensional
2D	Two-dimensional
3D	Three-dimensional
4-CP	4-Chlorophenol
ALD	Atomic layer deposition
AMOX	Amoxicillin
BCN	B-doped g-C_3_N_4_
BPA	Bisphenol A
CB	Conduction band
CBM	Conduction band maxima
CFZ	Cefazolin
CIP	Ciprofloxacin
CNT	Carbon nanotube
CNZ	g-C_3_N_4_/ZnO composite
CZg	ZnO/CuO/g-C_3_N_4_ heterostructure
CZN	g-C_3_N_4_/ZnO/NiFe_2_O_4_ heterostructure
DCDA	Dicyandiamide
DFT	Density functional theory
ESR	Electron spin resonance
FQs	Fluoroquinolone
FZCCN	Fe_2_O_3_-ZnO@C/g-C_3_N_4_ heterojunction
GA	Graphene aerogel
HNTs	Halloysite nanotubes
HRTEM	High-resolution transmission electron microscopy
ICP	Inductive Couple Plasma Emission Spectrometer
KCC	KAUST catalysis center
KIT	Korea Advanced Institute of Science and Technology
LED	Light-emitting diode
LSPR	Localized surface-plasmon resonance
MG	Malachite green
MO	Methyl orange
MOF	Metal–organic framework
MV	Methyl violet
NHE	Normal hydrogen electrode
NiZG	Ni/ZnO/g-C_3_N_4_ composite
NOR	Norfloxacin
OD	Oxygen defect
OP	Oxidation photocatalyst
RP	Reduction photocatalyst
OV	Oxygen vacancies
pc- GCN	P, C-codoped g-C_3_N_4_
PET	Polyester fiber
Ppm	Parts per million
QDs	Quantum dots
RGO, rGO	Reduced graphene
RhB	Rhodamine B
ROS	Reactive oxygen species
SBA-15	Santa Barbara Amorphous-15
SEM	Scanning electron microscopy
SMZ^−^	Negatively charged sulfonamide
SPR	Surface-plasmon resonance
S-scheme	Step-scheme
TC	Tetracycline
TEM	Transmission electron microscopy
TGA	Thermogravimetric analysis
UV light	Ultraviolet light
VB	Valence band
VBM	Valence band maxima
WFFDBD	Water falling film dielectric barrier discharge
WLLI	White LED light irradiation
XPS	X-ray photoelectron spectroscopy
XRD	X-ray diffraction
ZIF-8	Zeolitic imidazolate framework-8
ZPC	Zeta potential charge

## References

[1] Motelica L., Oprea O.-C., Vasile B.-S., Ficai A., Ficai D., Andronescu E., Holban A.M. (2023). Antibacterial Activity of Solvothermal Obtained ZnO Nanoparticles with Different Morphology and Catalytic Activity Against a Dye Mixture: Methylene Blue, Rhodamine B and Methyl Orange. Int. J. Mol. Sci..

[2] Panthi G., Ranjit R., Kim H.Y., Mulmi D.D. (2018). Size dependent optical and antibacterial properties of Ag_3_PO_4_ synthesized by facile precipitation and colloidal approach in aqueous solution. Optik.

[3] Cheruiyot G.K., Wanyonyi W.C., Kiplimo J.J., Mania E.N. (2019). Adsorption of toxic crystal violet dye using coffee husks: Equilibrium, kinetics and thermodynamics study. Sci. Afr..

[4] Panthi G., Park M., Kim H.Y., Park S.J. (2014). Electrospun Ag-CoF doped PU nanofibers: Effective visible light catalyst for photodegradation of organic dyes. Macromol. Res..

[5] Panthi G., Barakat N.A.M., Park M., Kim H.Y., Park S.J. (2015). Fabrication of PdS/ZnS NPs doped PVAc hybrid electrospun nanofibers: Effective and reusable catalyst for dye photodegradation. J. Ind. Eng. Chem..

[6] Panthi G., Park M. (2021). Electrospun carbon nanofibers decorated with Ag_3_PO_4_ nanoparticles: Visible-light driven photocatalyst for the photodegradation of methylene blue. Photochem.

[7] Lyulyukin M., Kovalevskiy N., Bhukhtiyarov A., Kozlov D., Selishchev D. (2023). Kinetic aspects of benzene degradation over TiO_2_-N and composite Fe/Bi_2_WO_6_/TiO_2_-N photocatalysts under irradiation with visible light. Int. J. Mol. Sci..

[8] Panthi G., Park M. (2022). Approaches for enhancing the photocatalytic activities of barium titanate: A review. J. Energy Chem..

[9] Carey J., Lawrence J., Tosine H. (1976). Photodechlorination of PCB’s in the presence of titanium dioxide in aqueous solutions. Bull. Environ. Contam. Toxicol..

[10] Liu S., Tang Z.R., Sun Y., Colmenares J.C., Xu Y.J. (2015). One dimension-based spatially order architectures for solar energy conversion. Chem. Soc. Rev..

[11] Zhao Y., Jia X., Waterhouse G.I.N., Wu L.Z., Tung C.H., O’Hare D., Zhang T. (2016). Layered double hydroxide nanostructured photocatalysts for renewable energy production. Adv. Energy Mater..

[12] Huo H., Li Y., Wang S., Tan S., Li X., Yi S., Gao L. (2022). Construction of highly active Zn_3_In_2_O_6_ (110)/g-C_3_N_4_ system by low temperature solvothermal for efficient degradation of tetracycline under visible light. Int. J. Mol. Sci..

[13] Jiang L., Yuan X., Zeng G., Chen X., Wu Z., Liang J., Zhang J., Wang H., Wang H. (2017). Phosphorus- and sulfur-codoped g-C_3_N_4_: Facile preparation, mechanism insight, and application as efficient photocatalyst for tetracycline and methyl orange degradation under visible light irradiation. ACS Sustain. Chem. Eng..

[14] Fu J., Yu J., Jiang C., Cheng B. (2018). g-C_3_N_4_-Based heterostructured photocatalysts. Adv. Energy Mater..

[15] Panthi G., Kwon O.H., Kuk Y.S., Gyawali K.R., Park Y.W., Park M. (2020). Ternary composite of Co-Doped CdSe@electrospun carbon nanofibers: A novel reusable visible light-driven photocatalyst with enhanced performance. Catalysts.

[16] Zhurenok E.A., Vasilchenko D.B., Kozlova E.A. (2023). Comprehensive review on g-C_3_N_4_-based photocatalysts for the photocatalytic hydrogen production under visible light. Int. J. Mol. Sci..

[17] Pérez-Molina Á., Pastrana-Martinez L.M., Pérez-Poyatos L.T., Morales-Torres S., Maldonado-Hódar F.J. (2022). One-Pot Thermal Synthesis of g-C_3_N_4_/ZnO Composites for the Degradation of 5-Fluoruracil Cytostatic Drug Under UV-LED Irradiation. Nanomaterials.

[18] Rashtizadeh A., Delnavaz M., Samadi A., Heidarzadeh N. (2023). Photodegradation of POPs-containing wastewater using sunlight driven Ce-doped-ZnO/g-C_3_N_4_ photocatalyst: Optimization, and cost-efficiency analysis. Chem. Phys. Lett..

[19] Kroke E., Schwarz M., Horath-Bordon E., Kroll P., Noll B., Norman A.D. (2002). Tri-s-triazine derivatives. Part I. From trichloro-tri-s-triazine to graphitic C_3_N_4_ structures. New J. Chem..

[20] Zhang H., Zuo X., Tang H., Li G., Zhou Z. (2015). Origin of photoactivity in graphitic carbon nitride and strategies for enhancement of photocatalytic efficiency: Insights from first principles computations. Phys. Chem. Chem. Phys..

[21] Wang X., Blechert S., Antonietti M. (2012). Polymeric graphitic carbon nitride for heterogeneous photocatalysis. ACS Catal..

[22] Solis R.R., Quintana M.A., Martin-Lara M.A., Pérez A., Calero M., Muñoz-Batista M.J. (2022). Boosted activity of g-C_3_N_4_/UiO-66-NH_2_ heterostructures for the photocatalytic degradation of contaminants in water. Int. J. Mol. Sci..

[23] Xiao J., Liu X., Pan L., Shi C., Zheng X., Zou J. (2020). Heterogeneous photocatalytic organic transformation reactions using conjugated polymers-based materials. ACS Catal..

[24] Zhou Y., Wang Z., Huang L., Zaman S., Lei K., Yue Y., Li Z., You B., Xia B. (2021). Engineering 2D photocatalysts toward carbon dioxide reduction. Adv. Energy Mater..

[25] Liang Z., Shen R., Ng Y., Zhang P., Xiang Q., Li X. (2020). A review on 2D MoS_2_ cocatalysts in photocatalytic H_2_ production. J. Mater. Sci. Technol..

[26] Panthi G., Park M., Kim H.Y., Lee Y.S., Park S.J. (2015). Electrospun ZnO hybrid nanofibers for photodegradation of wastewater containing organic dyes: A review. J. Ind. Eng. Chem..

[27] Motelica L., Vasile B.-S., Ficai A., Surdu A.-V., Ficai D., Oprea O.-C., Andronescu E., Jinga D.C., Holban A.M. (2022). Influence of the Alcohols on the ZnO Synthesis and Its Proterties: The Photocatalytic and Antimicrobial Activities. Pharmaceutics.

[28] Shim M., McDaniel H., Oh N. (2011). Prospects for strained type-II nanorod heterostructures. J. Phys. Chem. Lett..

[29] Xu C., Ravi Anusuyadevi P., Aymonier C., Luque R., Marre S. (2019). Nanostructured materials for photocatalysis. Chem. Soc. Rev..

[30] Zhao Z., Sun Y., Dong F. (2015). Graphitic carbon nitride-based nanocomposites: A review. Nanoscale.

[31] Xing W., Tu W., Han Z., Hui Y., Meng Q., Chen G. (2018). Template-induced high-crystalline g-C_3_N_4_ nanosheets for enhanced photocatalytic H2 evolution. ACS Energy Lett..

[32] Wang X., Maeda K., Thomas A., Takanabe K., Xin G., Carlsson J.M., Domen K., Antonietti M. (2009). A metal-free polymeric photocatalyst for hydrogen production from water under visible light. Nat. Mater..

[33] Teter D.M., Hemley R.J. (1996). Low-compressibility carbon nitrides. Science.

[34] Maeda K., Wang X., Nishihara Y., Lu D., Antonietti M., Domen K. (2009). Photocatalytic Activities of graphitic carbon nitride powder for water reduction and oxidation under visible light. J. Phys. Chem. C.

[35] Ajiboye T.O., Kuvarega A.T., Onwudiwe D.C. (2020). Graphitic carbon nitride-based catalysts and their applications: A review. Nano-Struct. Nano-Objects.

[36] Malina B., Sansores L.E. (1999). Electronic structure of six phases of C_3_N_4_: A theoretical approach. Mod. Phys. Lett. B.

[37] Martin D.J., Reardon P.J.T., Moniz S.J.A., Tang J. (2014). Visible light-driven pure water splitting by a nature-inspired organic semiconductor-based system. J. Am. Chem. Soc..

[38] Yan S.C., Li Z.S., Zou Z.G. (2009). Photodegradation Performance of g-C_3_N_4_ Fabricated by Directly Heating Melamine. Langmuir.

[39] Li G.S., Lian Z.C., Wang W.C., Zhang D.Q., Li H.X. (2016). Nanotube-confinement induced size-controllable g-C_3_N_4_ quantum dots modified single-crystalline TiO_2_ nanotube arrays for stable synergetic photoelectrocatalysis. Nano Energy.

[40] Shi L., Wang T., Zhang H., Chang K., Ye J. (2015). Electrostatic self-assembly of nanosized carbon nitride nanosheet onto a zirconium metal-organic framework for enhanced photocatalytic CO_2_ reduction. Adv. Funct. Mater..

[41] Wang X., Maeda K., Chen X., Takanabe K., Domen K., Hou Y., Fu X., Antonietti M. (2009). Polymer Semiconductors for Artificial Photosynthesis: Hydrogen Evolution by Mesoporous Graphitic Carbon Nitride with Visible Light. J. Am. Chem. Soc..

[42] Tong Z., Yang D., Li Z., Nan Y., Ding F., Shen Y., Jiang Z. (2017). Thylakoid-inspired multi-shell g-C_3_N_4_ nanocapsules with enhanced visible-light harvesting and electron transfer properties for high-efficiency photocatalysis. ACS Nano.

[43] Shiraishi Y., Kanazawa S., Sugano Y., Tsukamoto D., Sakamoto H., Ichikawa S., Hirai T. (2014). Highly selective production of hydrogen peroxide on graphitic carbon nitride (g-C_3_N_4_) photocatalyst activated by visible light. ACS Catal..

[44] Bai X., Yan S., Wang J., Wang L., Jiang W., Wu S., Sun C., Zhu Y.A. (2014). Simple and Efficient strategy for the Synthesis of a chemically tailored g-C_3_N_4_ material. J. Mater. Chem. A.

[45] Liang Q.H., Li Z., Huang Z.H., Kang F.Y., Yang Q.H. (2015). Holey graphitic carbon nitride nanosheets with carbon vacancies for highly improved photocatalytic hydrogen production. Adv. Funct. Mater..

[46] Liu G., Wang T., Zhang H., Meng X., Hao D., Chang K., Li P., Kako T., Ye J. (2015). Nature-inspired environmental phosphorylation boosts photocatalytic H_2_ production over carbon nitride nanosheets under visible-light irradiation. Angew. Chem. Int. Ed..

[47] Ong W.J., Tan L.L., Chai S.P., Yong S.T. (2015). Graphene oxide as a structure-directing agent for the two-dimensional interface engineering of sandwich-like graphene-g-C_3_N_4_ hybrid nanostructures with enhanced visible-light photoreduction of CO_2_ to methane. Chem. Commun..

[48] Zhang Y., Liu J., Wu G., Chen W. (2012). Porous graphitic carbon nitride synthesized via direct polymerization of urea for efficient sunlight-driven photocatalytic hydrogen production. Nanoscale.

[49] Liu J., Li W., Duan L., Li X., Ji L., Geng Z., Huang K., Lu L., Zhou L., Liu Z. (2015). A graphene-like oxygenated carbon nitride material for improved cycle-life lithium/sulfur batteries. Nano Lett..

[50] Zhang G., Zhang J., Zhang M., Wang X. (2012). Polycondensation of thiourea into carbon nitride semiconductors as visible light photocatalysts. J. Mater. Chem..

[51] Xiao J., Xie Y., Nawaz F., Wang Y., Du P., Cao H. (2016). Dramatic coupling of visible light with ozone on honeycomb-like porous g-C_3_N_4_ towards superior oxidation of water pollutants. Appl. Catal. B.

[52] Wang K., Li Q., Liu B., Cheng B., Ho W., Yu J. (2015). Sulfur-doped g-C_3_N_4_ with enhanced photocatalytic CO_2_-reduction performance. Appl. Catal. B.

[53] Xu J., Wu H.T., Wang X., Xue B., Li Y.X., Cao Y. (2013). A new and environmentally benign precursor for the synthesis of mesoporous g-C_3_N_4_ with tunable surface area. Chem. Chem. Phys..

[54] Chung Y.J., Lee B.I., Ko J.W., Park C.B. (2016). Photoactive g-C_3_N_4_ nanosheets for light-induced suppression of alzheimer’s beta-amyloid aggregation and toxicity. Adv. Healthc. Mater..

[55] Shan W., Hu Y., Bai Z., Zheng M., Wei C. (2016). In situ preparation of g-C_3_N_4_/bismuth-based oxide nanocomposites with enhanced photocatalytic activity. Appl. Catal. B.

[56] Long B.H., Lin J.L., Wang X.C. (2014). Thermally-induced desulfurization and conversion of guanidine thiocyanate into graphitic carbon nitride catalysts for hydrogen photosynthesis. J. Mater. Chem. A.

[57] Yuan S., Zhang Q., Xu B., Liu S., Wang J., Xie J., Zhang M., Ohno T. (2017). A new precursor to synthesize g-C_3_N_4_ with superior visible light absorption for photocatalytic application. Catal. Sci. Technol..

[58] Hao Q., Jia G., Wei W., Vinu A., Wang Y., Arandiyan H., Ni B.-J. (2020). Graphitic carbon nitride with different dimensionalities for energy and environmental applications. Nano Res..

[59] Wang S., Li J., Li Q., Bai X., Wang J. (2020). Metal single-atom coordinated graphitic carbon nitride as an efficient catalyst for CO oxidation. Nanoscale.

[60] Thomas A., Fischer A., Goettmann F., Antonietti M., Müller J.O., Schlögl R., Carlsson J.M. (2008). Graphitic carbon nitride materials: Variation of structure and morphology and their use as metal-free catalysts. J. Mater. Chem..

[61] Lotsch B.V., Schnick W. (2005). Thermal conversion of guanylurea dicyanamide into graphitic carbon nitride via prototype CNx precursors. Chem. Mater..

[62] Shi L.L., Wang L., Ma F., Sun J. (2014). Polycondensation of guanidine hydrochloride into a graphitic carbon nitride semiconductor with a large surface area as a visible light photocatalyst. J. Catal. Sci. Technol..

[63] Cui L., Liu Y., Fang X., Yin C., Li S., Sun D., Kang S. (2018). Scalable and clean exfoliation of graphitic carbon nitride in NaClO solution: Enriched surface-active sites for enhanced photocatalytic H_2_ evolution. Green Chem..

[64] Jun Y.S., Lee E.Z., Wang X., Hong W.H., Stucky G.D., Thomas A. (2013). From melamine-cyanuric acid supramolecular aggregates to carbon nitride hollow spheres. Adv. Funct. Mater..

[65] Shalom M., Inal S., Fettkenhauer C., Neher D., Antonietti M. (2013). Antonietti, Improving carbon nitride photocatalysis by supramolecular preorganization of monomers. J. Am. Chem. Soc..

[66] Yang J., Zhang X.Q., Xie C., Long J., Wang Y., Wei L., Yang X. (2021). Preparation of g-C_3_N_4_ with high specific surface area and photocatalytic stability. J. Electron. Mater..

[67] Wen J., Xie J., Chen X., Li X. (2017). A review on g-C_3_N_4_-based photocatalysts. Appl. Surf. Sci..

[68] Ong W.J., Tan L.L., Ng Y.H., Yong S.T., Chai S.P. (2016). Graphitic carbon nitride (g-C_3_N_4_)-based photocatalysts for artificial photosynthesis and environmental remediation: Are we a step closer to achieving sustainability?. Chem. Rev..

[69] Yan H. (2012). Soft-templating synthesis of mesoporous graphitic carbon nitride with enhanced photocatalytic H_2_ evolution under visible light. Chem. Commun..

[70] Pandiaraj S., Aiyappa H.B., Banerjee R., Kurungot S. (2014). Post modification of MOF derived carbon via g-C_3_N_4_ entrapment for an efficient metal-free oxygen reduction reaction. Chem. Commun..

[71] Gibot P., Schnell F., Spitzer D. (2016). Enhancement of the graphitic carbon nitride surface properties from calcium salts as templates. Micropor. Mesopor. Mater..

[72] Chen X., Shi R., Chen Q., Zhang Z., Jiang W., Zhu Y., Zhang T. (2019). Three-dimensional porous g-C_3_N_4_ for highly efficient photocatalytic overall water splitting. Nano Energy.

[73] Sun J., Zhang J., Zhang M., Antonietti M., Fu X., Wang X. (2012). Bioinspired hollow semiconductor nanospheres as photosynthetic nanoparticles. Nat. Commun..

[74] Huang J., Antonietti M., Liu J. (2014). Bio-inspired carbon nitride mesoporous spheres for artificial photosynthesis: Photocatalytic cofactor regeneration for sustainable enzymatic synthesis. J. Mater. Chem. A.

[75] Zhang J., Zhang M., Yang C., Wang X. (2014). Nanospherical carbon nitride frameworks with sharp edges accelerating charge collection and Separation at a soft photocatalytic interface. Adv. Mater..

[76] Gu Q., Liao Y., Yin L., Long J., Wang X., Xue C. (2015). Template-free synthesis of porous graphitic carbon nitride microspheres for enhanced photocatalytic hydrogen generation with high stability. Appl. Catal. B.

[77] Cao S., Low J., Yu J., Jaroniec M. (2015). Polymeric photocatalysts based on graphitic carbon nitride. Adv. Mater..

[78] Cui Y., Tang Y., Wang X. (2015). Template-free synthesis of graphitic carbon nitride hollow spheres for photocatalytic degradation of organic pollutants. Mater. Lett..

[79] Ma T.Y., Tang Y., Dai S., Qiao S.Z. (2014). Proton-functionalized two-dimensional graphitic carbon nitride nanosheet: An excellent metal-/label-free biosensing platform. Small.

[80] Xiao Y., Tian G., Li W., Xie Y., Jiang B., Tian C., Zhao D., Fu H. (2019). Molecule self-assembly synthesis of porous few-layer carbon nitride for highly efficient photoredox catalysis. J. Am. Chem. Soc..

[81] Deng S., Yang Z., Lv G., Zhu Y., Li H., Wang F., Zhang X. (2019). WO_3_ nanosheets/g-C_3_N_4_ nanosheets’ nanocomposite as an effective photocatalyst for degradation of rhodamine B. Appl. Phys. A.

[82] Zhang J.H., Hou Y.J., Wang S.J., Zhu X., Zhu C.Y., Wang Z., Li C.J., Jiang J.J., Wang H.P., Pan M. (2018). A facile method for scalable synthesis of ultrathin g-C_3_N_4_ nanosheets for efficient hydrogen production. J. Mater. Chem. A.

[83] Zhang X., Xie X., Wang H., Zhang J., Pan B., Xie Y. (2013). Enhanced photoresponsive ultrathin graphitic-phase C_3_N_4_ nanosheets for bioimaging. J. Am. Soc..

[84] Yang S., Gong Y., Zhang J., Zhan L., Ma L., Fang Z., Vajtai R., Wang X., Ajayan P.M. (2013). Exfoliated graphitic carbon nitride nanosheets as efficient catalysts for hydrogen evolution under visible light. Adv. Mater..

[85] Niu P., Zhang L., Liu G., Cheng H.M. (2012). Graphene-like carbon nitride nanosheets for improved photocatalytic activities. Adv. Funct. Mater..

[86] Liu J., Wang H., Chen Z.P., Moehwald H., Fiechter S., van de Krol R., Wen L., Jiang L., Antonietti M. (2015). Microcontact-printing-assisted access of graphitic carbon nitride films with favorable textures toward photoelectrochemical application. Adv. Mater..

[87] Jia L., Wang H., Dhawale D., Anand C., Wahab M.A., Ji Q., Ariga K., Vinu A. (2014). Highly ordered macro-mesoporous carbon nitride film for selective detection of acidic/basic molecules. Chem. Commun..

[88] Liu J., Huang J., Dontsova D., Antonietti M. (2013). Facile synthesis of carbon nitride micro-/nanoclusters with photocatalytic activity for hydrogen evolution. RSC Adv..

[89] Zheng Y., Lin L., Ye X., Guo F., Wang X. (2014). Helical graphitic carbon nitrides with photocatalytic and optical activities. Angew. Chem. Int. Ed..

[90] Li X.H., Zhang J., Chen X., Fischer A., Thomas A., Antonietti M., Wang X. (2011). Condensed graphitic carbon nitride nanorods by nanoconfinement: Promotion of crystallinity on photocatalytic conversion. Chem. Mater..

[91] Wang S., Li C., Wang T., Zhang P., Li A., Gong J. (2014). Controllable synthesis of nanotube-type graphitic C_3_N_4_ and their visible-light photocatalytic and fluorescent properties. J. Mater. Chem. A.

[92] He F., Chen G., Miao J., Wang Z., Su D., Liu S., Cai W., Zhang L., Hao S., Liu B. (2016). Sulfur-mediated self-templating synthesis of tapered C-PAN/g-C_3_N_4_ composite nanotubes toward efficient photocatalytic H_2_ evolution. ACS Energy Lett..

[93] Tong Z., Yang D., Sun Y., Nan Y., Jiang Z. (2016). Tubular g-C_3_N_4_ isotype heterojunction: Enhanced visible-light photocatalytic activity through cooperative manipulation of oriented electron and hole transfer. Small.

[94] Tahir M., Cao C., Mahmood N., Butt F.K., Mahmood A., Idrees F., Hussain S., Tanveer M., Ali Z., Aslam I. (2013). Multifunctional g-C_3_N_4_ nanofibers: A template-free fabrication and enhanced optical, electrochemical, and photocatalyst properties. ACS Appl. Mater. Interfaces.

[95] Groenewolt M., Antonietti M. (2005). Synthesis of g-C_3_N_4_ nanoparticles in mesoporous silica host matrices. Adv. Mater..

[96] Barman S., Sadhukhan M. (2012). Facile bulk production of highly blue fluorescent graphitic carbon nitride quantum dots and their application as highly selective and sensitive sensors for the detection of mercuric and iodide ions in aqueous media. J. Mater. Chem..

[97] Cao X., Ma J., Lin Y., Yao B., Li F., Weng W., Lin X. (2015). A facile microwave-assisted fabrication of fluorescent carbon nitride quantum dots and their application in the detection of mercury ions. Spectrochim. Acta A.

[98] Zhou J., Yang Y., Zhang C.-y. (2013). A low-temperature solid-phase method to synthesize highly fluorescent carbon nitride dots with tunable emission. Chem. Commun..

[99] Zhang S., Li J., Zeng M., Xu J., Wang X., Hu W. (2014). Polymer nanodots of graphitic carbon nitride as effective fluorescent probes for the detection of Fe^3+^ and Cu^2+^ ions. Nanoscale.

[100] Lu Y.C., Chen J., Wang A.J., Bao N., Feng J.J., Wang W., Shao L. (2015). Facile synthesis of oxygen and sulfur co-doped graphitic carbon nitride fluorescent quantum dots and their application for mercury (II) detection and bioimaging. J. Mater. Chem. C.

[101] Wang W., Jimmy C.Y., Shen Z., Chan D.K., Gu T. (2014). g-C_3_N_4_ quantum dots: Direct synthesis, upconversion properties and photocatalytic application. Chem. Commun..

[102] Zhang X.D., Wang H.X., Wang H., Zhang Q., Xie J.F., Tian Y.P., Wang J., Xie Y. (2014). Single-layered graphitic-C_3_N_4_ quantum dots for two-photon fluorescence imaging of cellular nucleus. Adv. Mater..

[103] Tong H., Ouyang S., Bi Y., Umezawa N., Oshikiri M., Ye J. (2012). Nanophotocatalytic materials: Possibilities and challenges. Adv Mater..

[104] Li H., Zhou Y., Tu W., Ye J., Zou Z. (2015). State-of-the-art progress in diverse heterostructured photocatalysts toward promoting photocatalytic performance. Adv. Funct. Mater..

[105] Bard A.J. (1997). Photoelectrochemistry and heterogeneous photo-catalysis at semiconductors. J. Photochem..

[106] Feng J., Ran X., Wang L., Xiao B., Lei L., Zhu J., Liu Z., Xi X., Feng G., Dai Z. (2022). The synergistic effect of adsorption-photocatalysis for removal of organic pollutants on mesoporous Cu_2_V_2_O_7_/Cu_3_V_2_O_8_/g-C_3_N_4_ heterojunction. Int. J. Mol. Sci..

[107] Wang L., Huang G., Zhang L., Lian R., Huang J., She H., Liu C., Wang Q. (2022). Construction of TiO_2_-covalent organic framework Z-scheme hybrid through coordination bond for photocatalytic CO_2_ conversion. J. Energy Chem..

[108] Maeda K. (2013). Z-scheme water splitting using two different semiconductor photocatalysts. ACS Catal..

[109] Zhou P., Yu J., Jaroniec M. (2014). All solid-state Z-scheme photocatalytic system. Adv. Mater..

[110] Tada H., Mitsui T., Kiyonaga T., Akita T., Tanaka K. (2006). All-solid-state Z-scheme in CdS-Au-TiO_2_ three-component nanojunction system. Nat. Mater..

[111] Li H., Yu H., Quan X., Chen S., Zhang Y. (2016). Transfer process of WO_3_-metal-gC_3_N_4_ (metal = Cu, Ag, Au). ACS Appl. Mater. Interfaces.

[112] Li H., Tu W., Zhou Y., Zou Z. (2016). Z-scheme photocatalytic system for promoting photocatalytic performance: Recent progress and future challenges. Adv. Sci..

[113] Li X., Yan X., Lu X., Zou S., Li Z., Yao C., Ni C. (2018). Photo-assisted selective catalytic reduction of NO by Z-scheme natural clay based photocatalyst: Insight into the effective graphene coupling. J. Catal..

[114] Iwase A., Ng Y.H., Ishiguro Y., Kudo A., Amal R. (2011). Reduced graphene oxide as a solid-state electron mediator in Z-scheme photocatalytic water splitting under visible light. J. Am. Chem. Soc..

[115] Yu J., Wang S., Low J., Xiao W. (2013). Enhanced photocatalytic performance of direct Z-scheme g-C_3_N_4_-TiO_2_ photocatalysts for the decomposition of formaldehyde in air. Phys. Chem. Chem. Phys..

[116] Bai S., Jiang J., Zhang Q., Xiong Y. (2015). Steering charge kinetics in photocatalysis: Interaction of materials synthesis, characterization technique and theoretical simulations. Chem. Soc. Rev..

[117] Liu J., Cheng B., Yu J. (2016). A new understanding of the photocatalytic mechanism of the direct Z-scheme g-C_3_N_4_/TiO_2_ heterostructure. Phys. Chem. Chem. Phys..

[118] Liang Y.C., You S.Y., Chen B.Y. (2022). Crystal design and photoactivity of TiO_2_ nanorod template decorated with nanostructured Bi_2_S_3_ visible light sensitizer. Int. J. Mol. Sci..

[119] Zhang X., Wang X., Chai J., Xue S., Wang R., Jiang L., Wang J., Zhang Z., Dionysiou D.D. (2020). Construction of novel symmetric double Z-scheme BiFeO_3_/CuBi_2_O_4_/BaTiO_3_ photocatalyst with enhanced solar-light driven photocatalytic performance for degradation of norfloxacin. Appl. Catal. B Environ..

[120] Xu Q., Zhang L., Cheng B., Fan J., Yu J. (2020). S-scheme heterojunction photocatalyst. Chem.

[121] Li Y., Zhou M., Cheng B., Shao Y. (2020). Recent advances in g-C_3_N_4_ based heterojunction photocatalysts. J. Mater. Sci. Technol..

[122] Fu J., Xu Q., Low J., Jiang C., Yu J. (2019). Ultrathin 2D/2D WO_3_/g-C_3_N_4_ step scheme H_2_-production photocatalyst. Appl. Catal. B.

[123] Zhang K., Zhou M., Yu C., Yang K., Li X., Dai W., Guan J., Shu Q., Huang W. (2020). Construction of S-scheme g-C_3_N_4_ heterostructures for enhancing photocatalytic disposal of pollutants and electrocatalytic hydrogen evolution. Dyes Pigments.

[124] Zhang X., Zhang Y., Jia X., Zhang N., Xia R., Zhang X., Wang Z., Yu M. (2021). In situ fabrication of a novel S-scheme heterojunction photocatalysts Bi_2_O_3_/P-C_3_N_4_ to enhance levofloxacin removal from water. Sep. Purif. Technol..

[125] Mei F., Dai K., Zhang J., Li W., Liang C. (2019). Construction of Ag SPR-promoted step scheme porous g-C_3_N_4_/Ag_3_VO_4_ heterojunction for improving photocatalytic activity. Appl. Surf. Sci..

[126] Jia X., Han Q., Zheng M., Bi H. (2019). One-pot milling route to fabricate step-scheme AgI/I-BiOAc photocatalyst: Energy band structure optimized by the formation of solid solution. Appl. Surf. Sci..

[127] Di G., Zhu Z., Zhang H., Zhu J., Qiu Y., Yin D., Küppers S. (2018). Visible-light degradation of sulfonamides by Z-scheme ZnO/g-C_3_N_4_ heterojunctions with amorphous Fe_2_O_3_ as electron mediator. J. Colloid Interf. Sci..

[128] Zhang J.Y., Mei J.Y., Yi S.S., Guan X.X. (2019). Constructing of Z-scheme 3D g-C_3_N_4_-ZnO@graphene aerogel heterojunctions for high-efficient adsorption and photodegradation of organic pollutants. Appl. Surf. Sci..

[129] Bai X., Li H., Zhang Z., Zhang X., Wang C., Xu J., Zhu Y. (2019). Carbon nitride nested-tube with graphene as dual electron mediator in Z-scheme photocatalytic deoxynivalenol degradation. Catal. Sci. Technol..

[130] Zhu P., Hu M., Duan M., Xie L., Zhao M. (2020). High visible light response Z-scheme Ag_3_PO_4_/g-C_3_N_4_/ZnO composite photocatalyst for efficient degradation of tetracycline hydrochloride: Preparation, properties and mechanism. J. Alloys Compd..

[131] Panthi G., Barakat N.A.M., Hazma A.M., Unnithan A.R., Motlak M., Khalil K.A., Shin Y.S., Kim H.Y. (2012). Polyaniline-poly(vinyl acetate) electrospun nanofibers mats as novel organic semiconductor material. Sci. Adv. Mater..

[132] Panthi G., Barakat N.A.M., Unnithan A.R., Al-Deyab S.S., Pant B., Nam K.T., Kim H.Y. (2012). Influence of gelatin on the wettability and mechanical properties of nylon-6 electrospun nanofibers: Novel mats for biomedical applications. J. Nanoeng. Nanomanuf..

[133] Panthi G., Ranjit R., Khadka S., Gyawali K.R., Kim H.Y., Park M. (2020). Characterization and antibacterial activity of rice grain-shaped ZnS nanoparticles immobilized inside the polymer electrospun nanofibers. Adv. Compos. Hybrid Mater..

[134] Panthi G., Barakat N.A.M., Al-Deyab S.S., El-Newehy M., Pandeya D.R., Kim H.Y. (2013). Interior synthesizing of ZnO nanoflakes inside nylon-6 electrospun nanofibers. J. Appl. Polym. Sci..

[135] Panthi G., Park S.J., Chung H.J., Park M., Kim H.Y. (2017). Silver nanoparticles decorated Mn_2_O_3_ hybrid nanofibers via electrospinning; towards the development of new bactericides with synergistic effect. Chem. Phys..

[136] Naseri A., Samadi M., Pourjavadi A., Ramakrishna S., Moshfegh A.J. (2021). Enhanced photocatalytic activity of ZnO/g-C_3_N_4_ nanofibers constituting carbonaceous species under simulated sunlight for organic dye removal. Ceram. Int..

[137] Bajiri M.A., Hezam A., Namratha K., Al-Maswari B.M., BhojyaNaik H.S., Byrappa K., Al-Zaqri N., Alsalme A., Alasmari R. (2021). Non-noble metallic Cu with three different roles in a Cu doped ZnO/Cu/g-C_3_N_4_ heterostructure for enhanced Z-scheme photocatalytic activity. New J. Chem..

[138] An X., Wang H., Dong C., Jian P., Wu Z., Yu B. (2022). Core-shell P-laden biochar/ZnO/g-C_3_N_4_ composite for enhanced photocatalytic degradation of atrazine and improved P slow-release performance. J. Colloid Interface Sci..

[139] Wu J., Hu H., Qian H., Li J., Yang R., Qu L. (2022). NiCo/ZnO/g-C_3_N_4_ Z-scheme heterojunction nanoparticles with enhanced photocatalytic degradation oxytetracycline. Diam. Relat. Mater..

[140] Shanthini K., Manivannan V., Govindaraju K.M., Collins Arun Prakash V., Lekshmi G.S., Govindan R. (2022). Fabrication of highly efficient g-C_3_N_4_/ZnO/Fe_2_O_3_ ternary composite with enhanced photocatalytic activity under visible light irradiation. J. Mater. Sci. Mater. Electron..

[141] Vignesh K., Kang S., Kwak B.S., Kang M. (2015). Meso-porous ZnO nano-triangles @ graphitic-C_3_N_4_ nano-foils: Fabrication and Recyclable photocatalytic activity. Sep. Purif. Technol..

[142] Wang J., Xia Y., Zhao H., Wang G., Xiang L., Xu J., Komarneni S. (2017). Oxygen defects-mediated Z-scheme charge separation in g-C_3_N_4_/ZnO photocatalysts for enhanced visible-light degradation of 4-chlorophenol and hydrogen evolution. Appl. Catal. B Environ..

[143] Li N., Tian Y., Zhao J., Zhang J., Zuo W., Kong L., Cui H. (2018). Z-scheme 2D/3D g-C_3_N_4_@ZnO with enhanced photocatalytic activity for cephalexin oxidation under solar light. Chem. Eng. J..

[144] Jung H., Pham T.T., Shin E.W. (2018). Interactions between ZnO nanoparticles and amorphous g-C_3_N_4_ nanosheets in thermal formation of g-C_3_N_4_/ZnO composite materials: The annealing temperature effect. Appl. Surf. Sci..

[145] Murugesan P., Girichandran N., Narayanan S., Manickam M. (2018). Structural, optical and photocatalytic properties of visible light driven zinc oxide hybridized two-dimensional p-conjugated polymeric gC_3_N_4_ composite. Opt. Mater..

[146] Tan X., Wang X., Hang H., Zhang D., Zhang N., Xiao Z., Tao H. (2019). Self-assembly method assisted synthesis of g-C_3_N_4_/ZnO heterostructure nanocomposites with enhanced photocatalytic performance. Opt. Mater..

[147] Fang Q., Li B., Li Y.Y., Huang W.Q., Peng W., Fan X., Huang G.F. (2019). 0D/2D Z-scheme heterojunctions of g-C_3_N_4_ quantum dots/ZnO nanosheets as a highly efficient visible-light photocatalyst. Adv. Powder Technol..

[148] Jung H., Pham T.T., Shin E.W. (2019). Effect of g-C_3_N_4_ precursors on the morphological structures of g-C_3_N_4_/ZnO composite photocatalysts. J. Alloys Compd..

[149] Zhang S., Su C., Ren H., Li M., Zhu L., Ge S., Wang M., Zhang Z., Li L., Cao X. (2019). In-situ fabrication of g-C_3_N_4_/ZnO nanocomposites for photocatalytic degradation of methylene blue: Synthesis procedure does matter. Nanomaterials.

[150] Guo X., Duan J., Li C., Zhang Z., Wang W. (2020). Highly efficient Z-scheme g-C_3_N_4_/ZnO photocatalysts constructed by co-melting-recrystallizing mixed precursors for wastewater treatment. J. Mater. Sci..

[151] Nandi P., Das D. (2020). Synthesis of cost-effective g-C_3_N_4_/ZnO heterostructure photocatalyst for methyl orange (MO) dye degradation. AIP Conf. Proc..

[152] Ramachandra M., Devi Kalathiparambil Rajendra Pai S., Resnik Jaleel U.C.J., Pinheiro D. (2020). Improved photocatalytic activity of g-C_3_N_4_/ZnO: A Potential direct Z-scheme nanocomposite. ChemistrySelect.

[153] Ngullie R.C., Alaswad S.O., Bhuvaneswari K., Shanmugam P., Pazhanivel T., Arunachalam P. (2020). Synthesis and characterization of efficient ZnO/g-C_3_N_4_ nanocomposites photocatalyst for photocatalytic degradation of methylene blue. Coatings.

[154] Shemeena M., Binitha N.N. (2020). Visible light active ZnO-g-C_3_N_4_ photocatalyst for dye pollutant degradation. Mater. Today Proc..

[155] Du Y., Yang C., Han H., Zhao Q., Jiang T. (2022). One-pot preparation of binary photocatalyst ZnO/g-C_3_N_4_ nanosheets with enhanced photocatalytic activity in dye degradation. ChemistrySelect.

[156] Gayathri1 K., Teja1 Y.N., Prakash R.M., Hossain M.S., Alsalme A., Sundaravadive E., Sakar M. (2022). In situ-grown ZnO particles on g-C_3_N_4_ layers: A direct Z-scheme-driven photocatalyst for the degradation of dye and pharmaceutical pollutants under solar irradiation. J. Mater. Sci. Mater. Electron..

[157] Brasileiro I.L.O., Madeira V.S., Lopes-Moriyama A.L., Ramalho M.L.R.A. (2023). Addition of g-C_3_N_4_ to ZnO and ZnFe_2_O_4_ to improve photocatalytic degradation of emerging organic pollutants. Ceram. Int..

[158] Silva F.F., Silva R.B., Silva T.R., Macedo D.A., Su B. (2023). Boosting the photocatalytic activity of g-C_3_N_4_/ZnO heterojunctions through optimal control of mass ratio. Solid State Sci..

[159] Girish Y.R., Udayabhanu, Byrappa N.M., Alnaggar G., Hezam A., Nagaraju G., Pramoda K., Byrappa K. (2023). Rapid and facile synthesis of Z-scheme ZnO/g-C_3_N_4_ heterostructure as efficient visible light-driven photocatalysts for dye degradation and hydrogen evolution reaction. J. Hazard. Mater. Adv..

[160] Mamari S.A., Khudaish E., Kim Y., Khraisheh M., Selvaraj R. (2023). Lotus-bud like hexagonal ZnO/g-C_3_N_4_ composites for the photodegradation of benzene present in aqueous solution. Inorg. Chem. Commun..

[161] An H., Huong L.M., Dat N.M., Hai N.D., Cong C.Q., Nam N.T.H., Tai L.T., Thi D.N.M., Nghi H.B., Huyen N.T.T. (2023). Photocatalytic degradation of organic dyes using zinc oxide-decorated graphitic carbon nitride composite under visible light. Diam. Relat. Mater..

[162] Skuta R., Kostura B., Ritz M., Foniok K., Pavlovský J., Matýsek D. (2023). Comparing the photocatalytic performance of GO/ZnO and g-C_3_N_4_/ZnO composites prepared using metallurgical waste as a source of zinc. Inorg. Chem. Commun..

[163] Azimi E.B., Badiei A., Sadr M.H. (2018). Dramatic visible photocatalytic performance of g-C_3_N_4_-based nanocomposite due to the synergistic effect of AgBr and ZnO semiconductors. J. Phys. Chem. Solids.

[164] Thang N.Q., Sabbah A., Chen L.C., Chen K.H., Thi C.M., Viet P.V. (2021). Highly efficient photocatalytic degradation of commercial drugs for pharmaceutical wastewater treatment prospects: A case study of Ag/g-C_3_N_4_/ZnO nanocomposite materials. Chemosphere.

[165] Balu S., Velmurugan S., Palanisamy S., Chen S.W., Velusamy V., Yang T.C.K., El-Shafey E.I. (2019). Synthesis of α-Fe_2_O_3_ decorated g-C_3_N_4_/ZnO ternary Z-scheme photocatalyst for degradation of tartrazine dye in aqueous media. J. Taiwan Inst. Chem. Eng..

[166] Hashem E.M., Hamza M.A., El-Shazly A.N., El-Rahman S.A.A., El-Tanany E.M., Mohamed R.T., Allam N.K. (2021). Novel Z-Scheme/Type-II CdS@ZnO/g-C_3_N_4_ ternary nanocomposites for the durable photodegradation of organics: Kinetic and mechanistic insights. Chemosphere.

[167] Vignesh S., Eniya P., Srinivasan M., Sundar J.K., Li H., Jayavel S., Pandiaraman M., Manthrammel M.A., Shkir M., Palanivel B. (2021). Fabrication of Ag/Ag_2_O incorporated graphitic carbon nitride based ZnO nanocomposite for enhanced Z-scheme photocatalytic performance of various organic pollutants and bacterial disinfection. J. Environ. Chem. Eng..

[168] Sher M., Khan S.A., Shahid S., Javed M., Qamar M.A., Chinnathambi A., Almoallim H.S. (2021). Synthesis of novel ternary hybrid g-C_3_N_4_@Ag-ZnO nanocomposite with Z-scheme enhanced solar light-driven methylene blue degradation and antibacterial activities. J. Environ. Chem. Eng..

[169] Madhushree R., UC J.R.J., Pinheiro D., Renuka N.K., Kr S.D., Park J., Manickam S., Choi M.Y. (2022). Architecture of visible-light induced Z-scheme MoS_2_/g-C_3_N_4_/ZnO ternary photocatalysts for malachite green dye degradation. Environ. Res..

[170] Asadi A., Daglioglu N., Hasani T., Farhadian N. (2022). Construction of Mg-doped ZnO/g-C_3_N_4_@ZIF-8 multi-component catalyst with superior catalytic performance for the degradation of illicit drug under visible light. Colloids Surf. A Physiochem. Eng. Asp..

[171] Nguyen T.X.Q., Chen S.S., Pasawan M., Chang H.M. (2023). Enhanced photocatalytic activity of g-C3N4-n-p type flower like ZnO/BiOBr heterojunction for hexavalent chromium and dye wastewater degradation. Environ. Technol. Innov..

[172] Panthi G., Hassan M., Kuk Y.S., Kim J.Y., Chung H.J., Hong S.T., Park M. (2020). Enhanced antibacterial property of sulfate-doped Ag_3_PO_4_ nanoparticles supported on PAN electrospun nanofibers. Molecules.

[173] Pascariu P., Cojocaru C., Samolia P., Romanitan C. (2023). Nd-doped ZnO nanostructures with enhanced photocatalytic performance for environmental protection. Int. J. Mol. Sci..

[174] Truc N.T.T., Duc D.S., Thuan D.V., Tahtamouni T.A., Pham T.D., Hanh N.T., Tran D.T., Nguyen M.V., Dang N.M., Chi N.T.P.L. (2019). The advanced photocatalytic degradation of atrazine by direct *Z*-scheme Cu doped ZnO/g-C_3_N_4_. Appl. Surf. Sci..

[175] Neena D., Humayun M., Bhattacharyya D., Fu D. (2020). Hierarchical Sr-ZnO/g-C_3_N_4_ heterojunction with enhanced photocatalytic activities. J. Photochem. Photobiol. A Chem..

[176] Sher M., Javed M., Shahid S., Iqbal S., Qamar M.A., Bahadur A., Qayyum M.A. (2021). The controlled synthesis of g-C_3_N_4_/Cd-doped ZnO nanocomposites as potential photocatalysts for the disinfection and degradation of organic pollutants under visible light irradiation. RSC Adv..

[177] Qamar M.A., Shahid S., Javed M., Iqbal S., Sher M., Bahadur A., AL-Anazy M.M., Laref A., Li D. (2021). Designing of highly active g-C_3_N_4_/Ni-ZnO photocatalyst nanocomposite for the disinfection and degradation of the organic dye under sunlight radiations. Colloids Surf. A Physiochem. Eng. Asp..

[178] Shen J.H., Chiang T.H., Tsai C.K., Jiang Z.W., Horng J.J. (2022). Mechanistic insights into hydroxyl radical formation of Cu-doped ZnO/g-C_3_N_4_ composite photocatalysis for enhanced degradation of ciprofloxacin under visible light: Efficiency, kinetics, products identification and toxicity evaluation. J. Environ. Chem. Eng..

[179] Luo Q., Sun Y., Lv X., Huang L., Fang L., Wang R. (2022). Creation of direct Z-scheme Al/Ga co-doping biphasic ZnO/g-C_3_N_4_ heterojunction for the sunlight-driven photocatalytic degradations of methylene blue. J. Sol-Gel Sci. Technol..

[180] Albadri A.E.A.E., Aissa M.A.B., Modwi A., Saleh S.M. (2023). Synthesis of mesoporous Ru-ZnO@g-C_3_N_4_ nanoparticles and their photocatalytic activity for methylene blue degradation. Water.

[181] Malik M., Ibrahim S.M., Nazir M.A., Tahir A.A., Tufail M.K., Shah S.S.A., Anum A., Wattoo M.A., Rehman A. (2023). Engineering of a hybrid g-C_3_N_4_/ZnO-W/Co_x_ heterojunction photocatalyst for the removal of methylene blue dye. Catalysts.

[182] Mohamed M.A., Zain M.F.M., Minggu L.J., Kassim M.B., Jaafar J., Amin N.A.S., Mastuli M.S., Wu H., Wong R.J., Ng Y.H. (2019). Bio-inspired hierarchical hetero-architectures of in-situ C-doped g-C_3_N_4_ grafted on C, N co-doped ZnO micro-flowers with booming solar photocatalytic activity. J. Ind. Eng. Chem..

[183] Kalisamy P., Lallimathi M., Suryamathi M., Palanivel B., Venkatachalam M. (2020). ZnO-embedded S-doped g-C_3_N_4_ heterojunction: Mediator-free Z-scheme mechanism for enhanced charge separation and photocatalytic degradation. RSC Adv..

[184] Kalisamy P., Hossain M.S., Macadangdang R.R., Madhubala V., Palanivel B., Venkatachalam M., Massoud E.E.S., Sreedevi G. (2022). ZnO coupled F-doped g-C_3_N_4_: Z-scheme heterojunction for visible-light driven photocatalytic degradation reaction. Inorg. Chem. Commun..

[185] Sareshkeh A.T., Some-Saraee R.B., Rasoulifard M.H., Seyed-Dorraji M.S., Hosseini S.F. (2022). P and C co-modified g-C_3_N_4_/SiO_2_/ZnO Z-scheme based heterogeneous nanocomposite as a highly boosted visible-light-driven photocatalytic system. J. Alloys Compd..

[186] Kumar S., Baruah A., Tonda S., Kumar B., Shanker V., Sreedhar B. (2014). Cost-effective and eco-friendly synthesis of novel and stable N-doped ZnO/g-C_3_N_4_ core-shell nanoplates with excellent visible-light responsive photocatalysis. Nanoscale.

[187] Liu Y., Liu H., Zhou H., Li T., Zhang L. (2019). A Z-scheme mechanism of N-ZnO/g-C_3_N_4_ for enhanced H_2_ evolution and photocatalytic degradation. Appl. Surf. Sci..

[188] Liu X., Liu L., Yao Z., Yang Z., Xu H. (2020). Enhanced visible-light-driven photocatalytic hydrogen evolution and NO photo-oxidation capacity of ZnO/g-C_3_N_4_ with N dopant. Colloids Surf. A.

[189] Alhanash A.M., Al-Namshah K.S., Mohamed S.K., Hamdy M.S. (2019). One-pot synthesis of the visible light sensitive C-doped ZnO@gC_3_N_4_ for high photocatalytic activity through Z-scheme mechanism. Optik.

[190] Behera P., Ray A., Tripathy S.P., Acharya L., Subudhi S., Parida K. (2023). ZIF-8 derived porous C, N co-doped ZnO modified B-g-C_3_N_4_: A Z-Scheme charge dynamics approach operative towards photocatalytic hydrogen evolution and ciprofloxacin degradation. J. Photochem. Photobiol. A.

[191] Bajiri M.A., Hezam A., Namratha K., Viswanath R., Drmosh Q.A., Naik H.S.B., Byrappa K. (2019). CuO/ZnO/g-C_3_N_4_ heterostructures as efficient visible light-driven photocatalysts. J. Environ. Chem. Eng..

[192] Wang M., Tan G., Ren H., Xia A., Liu Y. (2019). Direct double Z-scheme O-g-C_3_N_4_/Zn_2_SnO_4_N/ZnO ternary heterojunction photocatalyst with enhanced visible photocatalytic activity. Appl. Surf. Sci..

[193] Yaghoot-Nezhad A., Moradi M., Rostami M., Danaee I., Khosravi-Nikou M.R. (2020). Dual Z-scheme CuO-ZnO@Graphitic carbon nitride ternary nanocomposite with improved visible light-induced catalytic activity for ultrasound-assisted photocatalytic desulfurization. Energy Fuels.

[194] Moradi M., Hasanvandian F., Isari A.A., Hayati F., Kakavandi B., Setayesh S.R. (2021). CuO and ZnO co-anchored on g-C_3_N_4_ nanosheets as an affordable double Z-scheme nanocomposite for photocatalytic decontamination of amoxicillin. Appl. Catal. B Environ..

[195] Garg T., Renu, Kaur J., Kaur P., Nitansh, Kumar V., Tikoo K., Kaushik A., Singhal S. (2022). An innovative Z-scheme g-C_3_N_4_/ZnO/NiFe_2_O_4_ heterostructure for the concomitant photocatalytic removal and real-time monitoring of noxious fluoroquinolones. Chem. Eng. J..

[196] Aghababaei N., Abdouss M., Hosseini-Monfared H., Ghanbari F. (2023). Photocatalytic degradation of diclofenac using a novel double Z-scheme catalyst (O-g-C_3_N_4_/ZnO/TiO_2_@halloysite nanotubes): Degradation mechanism, identification of by-products and environmental implementation. J. Water Process. Eng..

[197] Liu X.Y., Li J. (2021). S-scheme heterojunction ZnO/g-C_3_N_4_ shielding polyester fiber composites for the degradation of MB. Semicond. Sci. Technol..

[198] Zhang C., Jia M., Xu Z., Xiong W., Yang Z., Cao J., Peng H., Xu H., Xiang Y., Jing Y. (2021). Constructing 2D/2D N-ZnO/g-C_3_N_4_ S-scheme heterojunction: Efficient photocatalytic performance for norfloxacin degradation. Chem. Eng. J..

[199] Elavarasan N., Vignesh S., Srinivasan M., Venkatesh G., Palanisamy G., Ramasamy P., Palanivel B., Al-Enizi A.M., Ubaidullah M., Reddy V.R.M. (2022). Synergistic S-scheme mechanism insights of g-C_3_N_4_ and rGO combined ZnO-Ag heterostructure nanocomposite for efficient photocatalytic and anticancer activities. J. Alloys Compd..

[200] Lee J.T., Lee S.W., Wey M.Y. (2022). S-scheme g-C_3_N_4_/ZnO heterojunction photocatalyst with enhanced photodegradation of azo dye. J. Taiwan Inst. Chem. Eng..

[201] Chai H., Yang C., Xu P., Wang P., Qu J., Zhang G. (2022). Enhanced visible-light photocatalytic activity with Fe_2_O_3_-ZnO@C/g-C_3_N_4_ heterojunction: Characterization, kinetics, and mechanisms. J. Clean. Prod..

[202] Sert B., Bilici Z., Ocakoglu K., Dizge N., Rad T.S., Khataee A. (2023). Preparation of S-scheme g-C_3_N_4_/ZnO heterojunction composite for highly efficient photocatalytic destruction of refractory organic pollutant. Catalysts.

[203] Feyzi L., Rahemi N., Allahyari S. (2022). Efficient degradation of tetracycline in aqueous solution using a coupled S-scheme ZnO/g-C_3_N_4_/zeolite P supported catalyst with water falling film plasma reactor. Process Saf. Environ. Prot..

[204] Leelavathi H., Muralidharan R., Abirami N., Tamizharasan S., Sankeetha S., Kumarasamy A., Arulmozhi R. (2023). Construction of step-scheme g-C_3_N_4_/Co/ZnO heterojunction photocatalyst for aerobic photocatalytic degradation of synthetic wastewater. Colloids Surf. A Physicochem. Eng. Asp..

[205] Shi S., Jia M., Li M., Zhou S., Zhao Y., Zhong J., Dai D., Qiu J. (2023). ZnO@g-C_3_N_4_ S-scheme photocatalytic membrane with visible-light response and enhanced water treatment performance. Colloids Surf. A Physicochem. Eng. Asp..

[206] Ahmad I., Shukrullah S., Naz M.Y., Bhatti H.N. (2023). Dual S-scheme ZnO-g-C_3_N_4_-CuO heterosystem: A photocatalyst for H_2_ evolution and wastewater treatment. React. Chem. Eng..

